# Rapid Classification of Sarcomas Using Methylation Fingerprint: A Pilot Study

**DOI:** 10.3390/cancers15164168

**Published:** 2023-08-18

**Authors:** Aviel Iluz, Myriam Maoz, Nir Lavi, Hanna Charbit, Omer Or, Noam Olshinka, Jonathan Abraham Demma, Mohammad Adileh, Marc Wygoda, Philip Blumenfeld, Masha Gliner-Ron, Yusef Azraq, Joshua Moss, Tamar Peretz, Amir Eden, Aviad Zick, Iris Lavon

**Affiliations:** 1 Leslie and Michael Gaffin Center for Neuro-Oncology, Hadassah Medical Center and Faculty of Medicine, The Hebrew University of Jerusalem, Jerusalem 9190501, Israel; 2 Agnes Ginges Center for Human Neurogenetics, Department of Neurology, Hadassah Medical Center and Faculty of Medicine, The Hebrew University of Jerusalem, Jerusalem 9190501, Israel; 3 Oncology Department, Sharett Institute of Oncology, Hadassah Medical Organization and Faculty of Medicine, The Hebrew University of Jerusalem, Jerusalem 9190501, Israel; 4 Department of Military Medicine and “Tzameret”, Faculty of Medicine, The Hebrew University of Jerusalem, Jerusalem 9190501, Israel; 5 Orthopedic Department, Hadassah Medical Organization and Faculty of Medicine, The Hebrew University of Jerusalem, Jerusalem 9190501, Israel; 6 Surgical Department, Hadassah Medical Organization and Faculty of Medicine, The Hebrew University of Jerusalem, Jerusalem 9190501, Israel; 7 Radiotherapy Institute, Sharett Institute of Oncology, Hadassah Medical Organization and Faculty of Medicine, The Hebrew University of Jerusalem, Jerusalem 9190501, Israel; 8 Radiology Department, Hadassah Medical Organization and Faculty of Medicine, The Hebrew University of Jerusalem, Jerusalem 9190501, Israel; 9 Department of Genetics, The Hebrew University of Jerusalem, Jerusalem 9190501, Israel

**Keywords:** sarcoma, nanopore, methylation, copy-number, classification, machine learning

## Abstract

**Simple Summary:**

Sarcomas encompass a diverse range of cancers, resulting in intricate classification that contributes to treatment delays. The aim of this pilot study, conducted within a specific subset of sarcoma types, is to demonstrates the feasibility of methylation and copy-number variation data obtained from low-coverage whole-genome sequencing using Oxford Nanopore for rapid point-of-care sarcoma classification. Oxford Nanopore sequencers are relatively affordable for laboratories, unlike other technologies used in previous studies for methylation-based sarcoma classification. Our findings indicate that this method attained an overall correct classification rate of 78%. This study could serve as the foundation for a rapid point-of-care sarcoma classification test, facilitating timely and efficient care across diverse clinical settings.

**Abstract:**

Sarcoma classification is challenging and can lead to treatment delays. Previous studies used DNA aberrations and machine-learning classifiers based on methylation profiles for diagnosis. We aimed to classify sarcomas by analyzing methylation signatures obtained from low-coverage whole-genome sequencing, which also identifies copy-number alterations. DNA was extracted from 23 suspected sarcoma samples and sequenced on an Oxford Nanopore sequencer. The methylation-based classifier, applied in the nanoDx pipeline, was customized using a reference set based on processed Illumina-based methylation data. Classification analysis utilized the Random Forest algorithm and t-distributed stochastic neighbor embedding, while copy-number alterations were detected using a designated R package. Out of the 23 samples encompassing a restricted range of sarcoma types, 20 were successfully sequenced, but two did not contain tumor tissue, according to the pathologist. Among the 18 tumor samples, 14 were classified as reported in the pathology results. Four classifications were discordant with the pathological report, with one compatible and three showing discrepancies. Improving tissue handling, DNA extraction methods, and detecting point mutations and translocations could enhance accuracy. We envision that rapid, accurate, point-of-care sarcoma classification using nanopore sequencing could be achieved through additional validation in a diverse tumor cohort and the integration of methylation-based classification and other DNA aberrations.

## 1. Introduction

Sarcoma is a cancer that originates from connective tissue [1]. Sarcomas are classified based on tissue and cell type and are typically divided into two major groups: bone sarcomas and soft-tissue sarcomas (STS) [2].

Sarcoma often presents as a painless mass that grows over months or years. Some types are more likely to affect children, while others affect mainly adults. Sarcomas can occur anywhere in the body, but the most common types occur in the arms, legs, and abdomen [3]. Generally, the cancer grade refers to its aggressiveness and the likelihood of spreading to other body parts. Low-grade sarcomas have a better prognosis than higher-grade sarcomas and are usually treated surgically, although sometimes radiation therapy or chemotherapy are used. Intermediate- and high-grade sarcomas are more frequently treated with surgery, chemotherapy, and radiation therapy. The treatment varies according to the exact type of sarcoma [4]. 

Diagnosis of bone sarcomas and soft-tissue sarcomas begins with a history, physical examination, and imaging studies [5]. Definitive diagnosis requires a tumor biopsy with extensive pathological review [4]. There is high inter-observer variability among pathologists. Using current pathological methods, up to 80–85% of sarcoma cases are classified, while the remaining cases remain unclassified [6]. Institutions with access to fluorescence in situ hybridization (FISH), Sanger sequencing, massively parallel DNA sequencing, and methylation-based arrays can gain a more accurate diagnosis by detecting point mutations, translocations, copy-number alterations [6,7], and methylation patterns [8]. 

Copy-number alterations in sarcomas are relatively uncommon, except for MDM2 amplification. The MDM2 gene is located on chromosome 12q13-15 and encodes the MDM2 protein. MDM2 amplification involves the presence of multiple copies of the MDM2 gene, which leads to elevated levels of MDM2 protein expression [9,10]. This amplification has been associated with heightened MDM2 protein expression and is linked to the process of de-differentiation in liposarcomas [11]. In de-differentiated liposarcoma, MDM2 is amplified in all tumors while in other tumors such as extraskeletal osteosarcoma MDM2 amplification is found in about 40% of the tumors. 

The identification of MDM2 amplification employs techniques such as fluorescence in situ hybridization (FISH) and immunohistochemistry (IHC) to detect MDM2 overexpression, serving as the gold standard methods [12]. 

The DNA of normal and tumor cell types in the body carries unique methylation marks correlating with its gene-expression profile, representing a fundamental aspect of tissue identity [13]. Numerous independent studies have shown that most central nervous system tumor types can be reliably identified based on their epigenetic DNA methylation. This layer of molecular information in neuropathological practice has increased accuracy and reduced the error rate in classifying CNS tumors [14]. A similar tool was developed for 54 histological types of sarcomas [8]. Most studies have used Illumina-based arrays for DNA methylation analysis, more specifically, the HumanMethylation450 with 485K CpGs or the MethylationEPIC with 850K CpGs. These arrays are based on DNA that undergoes bisulfite treatment that introduces specific changes in the DNA sequence that depend on the methylation status of individual cytosine residues and thus yield single-nucleotide resolution information about the methylation status [15]. 

The Oxford Nanopore sequencer can directly detect methylated base pairs (bp) without bisulfite modification. The sequencing of methylated bp is achieved by differentiating between the ionic current changes produced by unmethylated cytosine vs. 5-methylated cytosine [16]. Bisulfite-converted sequencing, which is the basis for Illumina Array, is a widely used method for detecting DNA methylation. Nonetheless, this approach has drawbacks including DNA degradation, limited specificity, and the production of short reads with low sequence diversity. In comparison, nanopore sequencing technology enables the direct detection of base modifications in native DNA, without requiring harsh chemical treatment as in bisulfite sequencing [17]. Moreover, nanopore technology allows the sequencing of longer DNA fragments up to about 100 kbp, allowing tumor classification based on methylation patterns and chromosomal aberrations [18]. It has been demonstrated that accurate and reliable CNS tumor classification can be performed based on methylation signatures gained by nanopore sequencing. Studies have shown that nanopore sequencing of low-coverage whole-genome sequencing (lcWGS) yielding a minimum set of 1000 random CpG sites chosen from the 450K sites, is sufficient for reliable brain tumor classification [19,20].

In this study, we investigated the utility of nanopore sequencing in classifying sarcomas. We successfully implemented and customized a nanopore-based nanoDx pipeline [19] to classify a restricted range of sarcoma types. The pipeline employs machine-learning algorithms for methylation-based classification. In addition, we utilize copy-number alteration to validate the classification of specific sarcoma types. 

## 2. Materials and Methods

### 2.1. Patients and DNA Isolation

23 Patients diagnosed with sarcoma between 2018 to 2023 who signed an informed consent form (0346-12) participated in this study. Surgically resected masses were freshly frozen, and a pathological report is available for all tumors with a molecular profile using Oncomine comprehensive panel (Thermo Fisher Scientific, Waltham, MA, USA) for some samples. Per the manufacturer’s protocol, we extracted tumor DNA using a DNeasy blood and tissue kit (Qiagen, Hilden, Germany). DNA was quantified by Qubit (Thermo Fisher Scientific) or QuantiFluor (Promega, Madison, WI, USA) assays and quality controlled (260/280 ratio) (NanoDrop, Thermo Fisher Scientific). 

### 2.2. Nanopore WGS

Between 200 and 400 ng of genomic tumor DNA of each sample is used for library preparation with barcode labeling using the Rapid Barcoding Kit (SQK-RBK004, Oxford Nanopore Technologies, Oxford, UK) according to the manufacturer’s instructions. Low-coverage whole-genome sequencing (lcWGS) is performed on a Minion Mk1C device (OS ubuntu 18.04) using an R9.4.1 flow cell (FLO-MIN106D, Oxford Nanopore Technologies). Sequencing was performed until the recommended 100M bps (per correspondence with the nanoDx pipeline developer [19]). Output FAST5 files containing the raw signal data were generated by the manufacturer’s software MinKNOW (v.22.12.5) and the equivalent FASTQ files. They were all transferred to high-performance computing (HPC) clusters for further analysis. 

### 2.3. Data Analysis Pipeline

FAST5 and FASTQ files of the assigned barcode were processed on the HPC using the nanoDx pipeline (v.5.0.1) that uses snakemake [19] v5.4.0 workflow [19,20]. This pipeline was initially developed for nanopore methylation-based classification and used the Heidelberg reference cohort of brain tumor methylation profiles of CpG sites probed by Illumina BeadChip 450K array (Illumina, Cambridge, UK) [14]. The nanoDx pipeline for brain tumor classification converts the methylation data from the 450K CpG sites to match the nanopore methylation data type for analysis. We adapted the pipeline to use sarcoma tumor methylation profiles obtained by the same Illumina platform from the cohort of 1077 sarcomas tumors [8]. We downloaded the beta-value processed data from GEO (GSE140686) and adapted it to the requirement of the pipeline code as a sarcoma reference set.

#### 2.3.1. Classification

1. Methylation-based Random Forest Classification

The processing of the FAST5 files calls the methylation status of genome-wide CpG sites of each sample using nanopolish software (v.0.13.2) [18]. The nanopolish software assigns a binary value of 1 or 0 to each detected CpG site, indicating methylation or unmethylation, respectively. This assignment is made through statistical analysis of the methylation detection algorithm [18]. The methylation frequency per site is then calculated by the fraction of reads classified as methylated. Given the fundamental differences between the Illumina Array-based methylation beta values and the nanopore methylation frequency values of the CpG sites, both data sources are subjected to binarization using a cutoff value of 0.6, consistent with previous nanoDx implementations [19,20] and are compatible with the nanopore methylation data type format. This enables the classification of each CpG site as either methylated (>0.6) or unmethylated. Subsequently, an ad-hoc Random Forest [20] classifier is trained using the most variable maximum of 100,000 overlapping sites within the sarcoma reference set.

**Figure 1:**
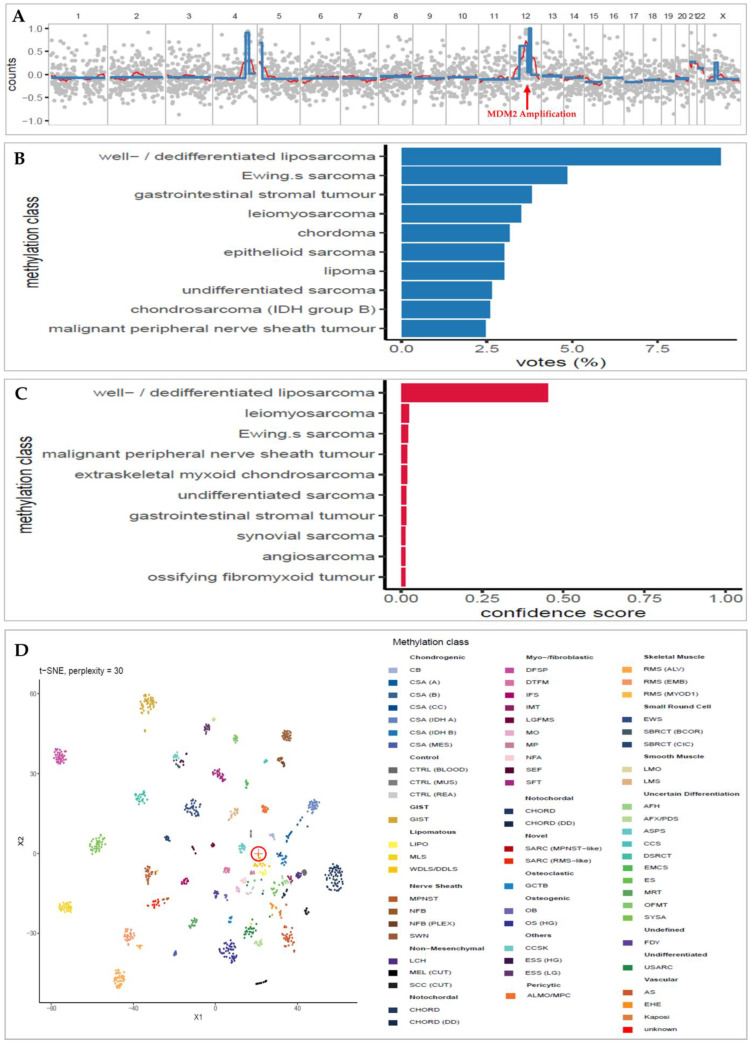
Extracts from an example of the final nanoDx analysis report for sample SARC-09 of a female patient with retroperitoneal MDM2 amplified well-differentiated liposarcoma. (**A**): Copy-number profile. The x-axis is the chromosomal location, and the y-axis is the read counts (log2 transformation). The MDM2 amplification is shown in chromosome 12. (**B**): Bar plot of the Random Forest classification voting results. The category (y) axis shows this sample’s 10 most frequent methylation classification votes and the percentage voting rate in the x-axis. (**C**): Bar plot of the confidence score of the voted Random Forest classification (in B). The most confident classification of this sample is “well/dedifferentiated liposarcoma” (WDLS/DDLS), with a confidence score of 0.45. This confidence score validates the top classification in the voting results (B) as a correct one. (**D**): t-SNE plot shows the clustering of the methylation pattern of the specific sample (circled cross sign) among the other methylation patterns of the sarcomas in the reference set. It shows that the sample clusters very close to the WDLS/DDLS group (dark yellow), as classified by the Random Forest classifier. Abbreviation: AFH, angiomatoid fibrous histiocytoma; AFX/PDS, atypical fibroxanthoma/pleomorphic dermal sarcoma; ALMO/MPC, angioleiomyoma/myopericytoma; AS, angiosarcoma; ASPS, alveolar soft part sarcoma; CB, chondroblastoma; CCS, clear cell sarcoma of soft parts; CCSK, clear cell sarcoma of the kidney; CHORD, chordoma; CHORD (DD), chordoma (dedifferentiated); CSA (A), chondrosarcoma (group A); CSA (B), chondrosarcoma (group B); CSA (CC), chondrosarcoma (clear cell); CSA (IDH A), chondrosarcoma (IDH group A); CSA (IDH B), chondrosarcoma (IDH group B); CSA [25], chondrosarcoma (mesenchymal); CTRL (BLOOD), control (blood); CTRL [26], control (muscle tissue); CTRL (REA), control (reactive tissue); DFSP, dermatofibrosarcoma protuberans; DSRCT, desmoplastic small round cell tumor; DTFM, desmoid-type fibromatosis; EHE, epithelioid hemangioendothelioma; EMCS, extraskeletal myxoid chondrosarcoma; ES, epithelioid sarcoma; ESS (HG), endometrial stromal sarcoma (high grade); ESS [27], endometrial stromal sarcoma (low grade); EWS, Ewing’s sarcoma; FDY, fibrous dysplasia; GCTB, giant cell tumor of bone; GIST, gastrointestinal stromal tumor; IFS, infantile fibrosarcoma; IMT, inflammatory myofibroblastic tumor; Kaposi, Kaposi sarcoma; LCH, Langerhans cell histiocytosis; LGFMS, low-grade fibromyxoid sarcoma; LIPO, lipoma; LMO, leiomyoma; LMS, leiomyosarcoma; MEL (CUT), melanoma (cutaneous); MLS, myxoid liposarcoma; MO, myositis ossificans; MP, myositis proliferans; MPNST, malignant peripheral nerve sheath tumor; MRT, malignant rhabdoid tumor; NFA, nodular fasciitis; NFB, neurofibroma; NFB (PLEX), neurofibroma (plexiform); OB, osteoblastoma; OFMT, ossifying fibromyxoid tumor; OS (HG), osteosarcoma (high grade); RMS [4], rhabdomyosarcoma (alveolar); RMS [28], rhabdomyosarcoma (embryonal); RMS (MYOD1), rhabdomyosarcoma (MYOD1); SARC (MPNST-like), sarcoma (MPNST-like); SARC (RMS-like), sarcoma (RMS-like); SBRCT (BCOR), small blue round cell tumor with BCOR alteration; SBRCT [29], small blue round cell tumor with CIC alteration; SCC (CUT), squamous cell carcinoma (cutaneous); SEF, sclerosing epithelioid fibrosarcoma; SFT, solitary fibrous tumor; SWN, schwannoma; SYSA, synovial sarcoma; USARC, undifferentiated sarcoma; WDLS/DDLS, well/dedifferentiated liposarcoma; t-SNE, t-distributed stochastic neighbor embedding.

The Random Forest classifier is built in Python using the RandomForestClassifier function from the scikit-learn package v.1.0.2 [21]. The classifier is then used to predict the methylation class of each sample. The Random Forest-estimated class probabilities are rescaled to be more accurately interpreted as confidence levels or “confidence scores” by the CalibratedClassifierCV function from the scikit-learn package in Python, as previously described [20]. Based on previous research conducted on CNS tumors, a confidence score greater than the threshold value of 0.15 is regarded as a reliable classification (see Discussion Section 4) [20] (Figure 1B,C). The sarcoma reference set was generated in the ‘HDF5’ binary data format, adhering to the pipeline’s specifications, using R/Bioconductor and the rhdf5 package [22].

2. Unsupervised Clustering

Additional unsupervised clustering analysis using t-SNE (t-distributed stochastic neighbor embedding) was performed on the 50,000 most variable CpG sites, and a final plot was generated using the R package Rtsne [23] (Figure 1D). Yet, t-SNE plots are meant for visual quality control, not classification. It can help validate the Random Forest classification results but must be interpreted carefully (per correspondence with the nanoDx pipeline developer [19]).

#### 2.3.2. Copy-Number Analysis

Briefly (as of [19,20]), FASTQ files are aligned to the hg19 human reference genome (minimap2 v2.15) [24] for the generation of copy-number profile (Figure 1A) which is generated from the same sequencing run using R/Bioconductor and the QDNAseq package [21]. Reads with a minimum mapping quality of 20 were sorted into 1000 kbp bins and analyzed using public data from a single flow cell sequencing run (FAF04090) generated with NA12878 reference DNA [22] for pseudo-germline subtraction. The circular binary segmentation method, implemented in the PSCBS R package, was utilized for the analysis. Change points were accepted based on an alpha value < 0.05. Arm-level copy-number calls were made by calculating the segment length weighted mean log ratio per chromosome arm.

#### 2.3.3. Reporting

All the analysis and classification results are reported in a PDF file. Extracts from a typical PDF report are depicted in Figure 1. The full report format of all cases is shown in File S1.

## 3. Results

### 3.1. DNA Extracted from Sarcoma Surgical Samples Are Successfully Utilized for Nanopore Sequencing

Out of the 23 samples, 20 were successfully run using nanopore and met the minimum sequencing coverage required to be processed by the nanoDx pipeline (see Methods). These include 18 tumors representing a limited range of 11 pathologically identified sarcoma types and 2 masses with no tumor; hence, we included in the statistical analysis the 18 tumor samples (Table 1). The included samples had a mean read length of 3966 bp (range 1310–7078), the mean number of CpG sites covered is 27594 (range 6539–100,000, Table S1), and the mean coverage is 0.53X (range 0.03X–6.4X).

The 2 non-tumor samples (not shown in Table 1) were analyzed in the pipeline. They have a mean read length of 1262 and 5769 bp, several CpG sites covered 7436 and 20,895, and coverage of 0.04X and 0.08X, respectively (Table S1).

### 3.2. Low-Overage DNA Methylation Successfully Classifies Sarcoma

The classification of sarcoma samples using the nanoDx Random Forest exhibited a concordance rate of 78% (14/18) with the pathological report (Table 1; File S1). The median confidence score of the Random Forest voting of the concordant classifications is 0.18 (range 0.08–0.88), with half above the threshold (0.15) considered the correct classification of CNS tumors. The Random Forest mean voting rate of the most confident concordant classifications is 11.23% (4.4–27.6%, Table 1).

Of the 18 samples analyzed, 4 (22%) exhibit discordant classification with the pathology report. One (SARC-07) is classified as a Ewing sarcoma (EWS) with a confidence score of 0.91 and a Random Forest voting rate of 30%. According to the pathologist, it is a small round blue cell tumor with no *EWS* translocation detected using FISH. 

The two Myxofibrosarcomas samples (SARC-12, SARC-22) were not classified correctly, as well as the chondrosarcoma sample (SARC-13). In these three samples, the confidence score is 0.04 to 0.07.

The two non-tumor samples were discordantly classified as undifferentiated sarcoma (USARC) and malignant peripheral nerve sheath tumor (MPNST) with confidence scores of 0.03 and 0.05, respectively (Table S1; File S1). 

### 3.3. t-SNE Unsupervised Clustering Matches Concordant Classifications

Based on the methylation fingerprint, out of the 14 cases that exhibited concordant classification with the pathology report, 12 (86%) were also clustered by the t-SNE analysis in agreement with the Random Forest classification (Table 1). Of the 4 discordant Random Forest classifications, 2 were not clustered by t-SNE analysis in agreement with the classification. They both had a low-confidence score of 0.04. 

Notably, there is a discordant case (SARC-07) in which the final pathological report disagreed with the Random Forest classification. Nevertheless, there is an agreement between the t-SNE clustering and the Random Forest classification. This case achieved the highest confidence score of 0.91 and the highest Random Forest voting rate of 30%.

All the cases in the cohort where the t-SNE clustering disagreed with the Random Forest classification had low-confidence scores (range 0.04–0.10). 

The t-SNE clustering also disagreed with the classification of the 2 non-tumor samples (excluded from the cohort) that also achieved low-confidence scores (0.03, 0.05).

### 3.4. Copy-Number Analysis Detects Typical Sarcoma Alteration

In 5 samples (28%), copy-number analysis identified MDM2 amplification, as depicted in Figure 1A and Table 1. All samples with MDM2 amplifications were classified under the methylation class of ‘well/dedifferentiated liposarcoma’ (WDLS/DDLS), characterized by MDM2 amplification [7]. In these instances, the MDM2 amplification validates the classification results. In one well-differentiated liposarcoma sarcoma, MDM2 amplification is not identified. The analysis of copy-number variations did not produce any particular modifications linked to alternative subtypes of sarcoma. 

## 4. Discussion

This pilot study presents several key findings. First, in a cohort of 23 surgically resected sarcoma tumors, 20 were successfully sequenced using an Oxford Nanopore device with low-coverage whole-genome sequencing (lcWGS). Out of the 20 samples, 2 did not contain tumor tissue, and 18 tumors were included in the study. The 18 tumors included a limited range of 11 pathologically identified sarcoma types. Among the 18 tumors, 14 were classified in agreement with the pathological report based on their methylation fingerprints. Copy-number alterations were also detected from the same sequencing data and were used to validate the classification. Specifically, MDM2 amplification is successfully identified in five out of six liposarcomas [7].

Our results were accomplished by tailoring the nanoDx classification pipeline specifically for sarcoma tumors [19,20]. We achieved a significant concordant classification rate of 78% (14/18) for the restricted sarcoma types by making a single effective adjustment to the pipeline incorporating our in-house-built sarcoma reference set. This reference set is generated using Illumina Array data [8]. Although Illumina Array has limitations, as discussed in the Introduction section [17], it currently stands as our sole data source for constructing the machine-learning sarcoma training set for methylation-based.

It’s worth noting that no changes were made to parameters related to the training of the Random Forest, such as the minimum number of CpG sites required for training or other hyperparameters [20]. The successful implementation of nanopore methylation-based classification using the nanoDx pipeline in this study for sarcomas indicates its potential applicability to other cancer types. It implies that low-pass methylation data obtained through nanopore sequencing might be meaningful and adequate to achieve a satisfactory classification rate using the Random Forest classification approach in other cancers. This potentially can be achieved by making similar adaptations to the current pipeline. However, as Koelsche and von Deimling pointed out, applying a methylation-based approach in hematopoietic tumors may present greater challenges than CNS or mesenchymal-derived tumors. This is primarily due to the already well-established classification system in hematopoietic tumors, which heavily relies on specific mutational events. Since individual mutations do not influence cellular methylation patterns in most cases, further evidence is needed to demonstrate their contribution to the existing classification system of hematopoietic tumors [30].

In CNS nanopore methylation-based classification, a confidence score is implemented. A platform-specific threshold is determined to ensure a more precise interpretation of the classifier results in a clinical context. The classification above a confidence score of 0.15 is considered reliable [20]. This study’s median confidence score for the concordant cases is 0.18 (range 0.08–0.87), comparable to the confidence score observed in CNS classification. However, a nanopore-specific confidence score threshold for accurate interpretation has not yet been determined for sarcomas. Establishing such a threshold will help ensure a precise interpretation of the nanopore methylation-based classification results in sarcoma cases. 

Factors such as low tumor cell content and DNA quality can influence confidence score values [20]. The current nanopore sequencing method is primarily optimized for fresh tissue samples from biopsies or collected during surgical procedures [20,30]. Using only fresh tissues is a limitation that restricts the ability to select sample regions with high tumor purity. Consequently, this can result in a methylation pattern that significantly deviates from that of cancerous tissue leading to a non-valid classification that is indicated by a low-confidence score. Furthermore, the possibility that the sample belongs to unknown sarcoma entities or different tumor types not represented in the Random Forest classifier training set [8,23] can also contribute to lower confidence scores and potentially discordant classifications.

Genomic alterations detected by copy-number analysis can help achieve more reliable classification, particularly in low-confidence score cases. In our results, six cases with low-confidence scores (range 0.08–0.14) were also classified in agreement with the t-SNE clustering analysis and the pathology report. In three of them, which are classified as liposarcoma (WDLS/DDLS), we identified the MDM2 amplification. This emphasizes the added value of the copy-number profile in validating the methylation-based classification results, mainly when a low-confidence value is achieved. Thus, as pointed out in [8,30], developing additional classifiers combining methylation patterns with other molecular parameters such as sequencing data, proteomic signatures, and histology might increase diagnostic accuracy.

In four cases (22%), the pathology report disagrees with the Random Forest classifications. Three of these cases achieved the lowest confidence score in the cohort (range 0.04–0.07). Moreover, the two non-tumor samples excluded from the cohort achieved a similar low-confidence score (0.03–0.05). Overall, all the cases that achieved a confidence score below 0.08 disagreed with the Random Forest classifications. This might imply that a confidence score lower than 0.08 indicates a non-valid classification in sarcoma. Still, this hypothesis should be tested in further research.

Particular attention should be focused on a specific instance of discordant classification referred to as SARC-07. In this case, the EWS class obtained a confidence score of 0.91. Notably, this class’s Random Forest voting rate was remarkably high, at 30%, the highest among the entire cohort. Furthermore, the t-SNE clustering result also aligned with the assigned classification. The pathology report for SARC-07 classifies it as a small round blue cell sarcoma (SRBCS) without *EWS* translocations through FISH analysis. EWS, which frequently manifests as SRBCS, typically involves the prototypical translocation of the *ESWR1* gene with genes from the ETS family [31]. An additional SRBCS type, based on distinct pathology, molecular analysis, and clinical observations indicating a highly aggressive clinical course [32,33], is a distinct entity termed “*CIC* rearranged sarcoma” [34]. Given these findings, we suspect SARC-07 might be a case of *CIC* rearranged sarcoma, warranting molecular reassessment. Further investigation could potentially result in the reclassification of this case, leading to its inclusion among the concordant cases.

The findings of this pilot study should be interpreted with caution, considering the following limitations:

First, the hyperparameters of the Random Forest machine-learning algorithm, such as the minimum number of CpG sites required for model training and confidence score threshold, were determined based on the analysis of CNS tumor methylation data obtained by nanopore sequencing [19,20]. These hyperparameters were not adjusted or explicitly recalibrated to analyze sarcoma nanopore methylation data. Consequently, there is potential to enhance the classification process by rescaling these parameters specifically for sarcoma data. 

Second, this study’s limited size and diversity of the sarcoma cohort do not adequately represent the wide range of sarcoma types. Therefore, it is impossible to definitively claim that this customized classification pipeline is suitable for reliably classifying all sarcoma subtypes based on nanopore lcWGS methylation data. 

Last, it is essential to note that the current reference set used in this study comprised 62 sarcoma methylation classes [8]. The analysis of additional sarcoma samples will contribute to further improvement of this tool [30].

However, it is essential to underscore that achieving a more accurate sarcoma classification can be facilitated by integrating supplementary layers of sarcoma-related data into the statistical analysis. These layers involve transcriptomic and proteomic analyses and consider pathological features and metabolic characteristics. Additionally, incorporating supplementary genomic and molecular data, such as copy-number alterations and point mutations, should be explored in conjunction with the current methylation data.

Despite these limitations, the classification concordance rate is significant, relying only on nanopore methylation data and minimal pipeline adaptations for sarcomas. 

## 5. Conclusions

Previous studies have established the validity of methylation-based classification using Illumina Array methylation data, particularly in sarcomas and CNS tumors [8,14]. Illumina Array has limitations such as GC bias, time-consuming procedures, and reliance on central high-volume laboratories. In contrast, nanopore sequencing devices offer compact, rapid, and accessible technology that detects methylation patterns, point mutations, translocations, and copy-number alterations [16,18]. In the future, we expect that upcoming studies involving nanopore methylation data could provide more suitable and accurate information, potentially presenting an alternative to the currently employed Illumina-based methylation data.

In this study, we successfully customized the nanopore methylation-based classification pipeline for a restricted range of 11 pathologically identified sarcoma tumor types. This highlights its potential for aiding in the timely diagnosis of sarcoma. However, further validation is necessary across a broader range of tumors and in different centers, along with appropriate statistical refinement tailored for sarcomas. A more elaborate classifier that combines methylation patterns with sarcoma-specific CNA, translocations, point mutations, and additional multi-omic data layers is expected to increase accuracy further. 

With these advancements, this method can potentially add to sarcoma diagnosis, providing accurate classification in a faster, point-of-care manner. Furthermore, rapid detection of methylation patterns, copy-number alterations, and translocation might be used in the future to plan patient-specific cell-free DNA biomarkers and shed light on sarcoma biology.

## Figures and Tables

**Table 1:** Results of the comparisons conducted between the nanoDx methylation-based classification and pathology diagnosis for the study samples (n = 18). See Table S1 for more details.

Sample	Max Calibrated Meth. Class	Pathology	Pathology Meth. Class	Concordance	Max Confidence Score	Mean Read Length	Mean Coverage	MDM2 Ampl.	t-SNE Agreed Cluster
**SARC-01**	WDLS/DDLS	Well differentiated liposarcoma	WDLS/DDLS	C	0.09	5192	0.43	Y	Y
**SARC-02**	MLS	myxoid liposarcoma	MLS	C	0.30	5038	6.4		Y
**SARC-03**	LMS	leiomyosarcoma	LMS	C	0.21	5679	1.78		Y
**SARC-04**	USARC	Undifferentiated small round spindle cell sarcoma	USARC	C	0.08	1957	0.08		
**SARC-05**	EMCS	Extraskeletal myxoid chondrosarcoma	EMCS	C	0.88	4537	0.11		Y
**SARC-06**	WDLS/DDLS	Dedifferentiated liposarcoma	WDLS/DDLS	C	0.08	1310	0.05	Y	Y
**SARC-08**	WDLS/DDLS	Well differentiated liposarcoma	WDLS/DDLS	C	0.10	5121	0.03		
**SARC-09**	WDLS/DDLS	Highly suspicious for well differentiated liposarcoma	WDLS/DDLS	C	0.45	5564	0.07	Y	Y
**SARC-10**	EWS	Ewing´s sarcoma	EWS	C	0.14	3081	0.06		Y
**SARC-11**	WDLS/DDLS	Well differentiated liposarcoma	WDLS/DDLS	C	0.15	5244	0.04	Y	Y
**SARC-17**	MLS	Myxoid liposarcoma	MLS	C	0.36	5900	0.06		Y
**SARC-18**	CSA (A)	Chondrosarcoma	CSA (A)	C	0.53	2638	0.05		Y
**SARC-19**	WDLS/DDLS	Well differentiated liposarcoma	WDLS/DDLS	C	0.14	7078	0.05	Y	Y
**SARC-21**	SYSA	synovial sarcoma	SYSA	C	0.23	2623	0.04		Y
**SARC-13**	MPNST	chondrosarcoma	CHORD	D	0.04	1774	0.06		
**SARC-12**	WDLS/DDLS	Myxofibrosarcoma	USARC	D	0.07	3072	0.06		Y
**SARC-22**	AFH	Myxofibrosarcoma	USARC	D	0.04	2749	0.04		
**SARC-07**	EWS	Unclassified spindle-round cell sarcoma	SRBCS	D	0.91	2830	0.08		Y

Abbreviations: AFH, angiomatoid fibrous histiocytoma; CHORD, chordoma; CSA (A), chondrosarcoma (group A); EMCS, extraskeletal myxoid chondrosarcoma; EWS, Ewing’s sarcoma; LMS, leiomyosarcoma; MLS, myxoid liposarcoma; MPNST, malignant peripheral nerve sheath tumor; SFT, solitary fibrous tumor; SYSA, synovial sarcoma; USARC, undifferentiated sarcoma; WDLS/DDLS, well/dedifferentiated liposarcoma; Meth, methylation; Ampl, amplification; Y, yes; C, concordant; D, discordant.

## Data Availability

Sarcoma reference set data (.h5) will be available upon reasonable request.

## Supplementary Materials

The following supporting information can be downloaded at: https://www.mdpi.com/article/10.3390/cancers15164168/s1, File S1: Final nanoDx reports of all 23 cases, including an extract of the pathology microscopic report; Table S1: Supplemental data including additional sequencing and classification information.
