# Supplementary material for: Rapid Classification of Sarcomas Using Methylation Fingerprint: A Pilot Study
Source: Cancers (Basel). 2023 Aug 18;15(16):4168. doi: 10.3390/cancers15164168 (PMC10453223; doi:10.3390/cancers15164168)
Supplement: Supplementary file 1 [file cancers-15-04168-s001.zip › File S1.pdf]

Nanopore sequencing report

SARC-01

Quality control metrics

Barcode statistics

```
Adapters detected in 260581 of 264830 reads
         RBK004 260581: |  ################### |  98.40 %
           none   4249: |                      |   1.60 %
Barcodes detected in 260581 of 264830 adapters
      barcode01 260581: |  ################### |  98.40 %
           none   4249: |                      |   1.60 %
Demultiplexing finished in 153.38s
```

Read statistics

```
General summary:
Average percent identity:             91.5
Fraction of bases aligned:              1.0
Mean read length:                  5,191.8
Mean read quality:                    11.8
Median percent identity:              92.6
Median read length:                3,211.0
Median read quality:                  11.9
Number of reads:                 265,095.0
Read length N50:                   9,537.0
STDEV read length:                 5,752.7
Total bases:               1,376,327,224.0
Total bases aligned:       1,325,176,798.0
Number, percentage and megabases of reads above quality cutoffs
>Q5:    265003 (100.0%) 1376.3Mb
```

Mean genome coverage is 0.43X.

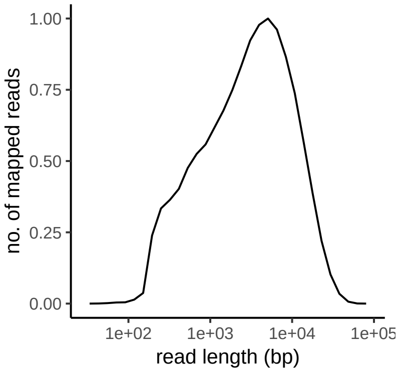

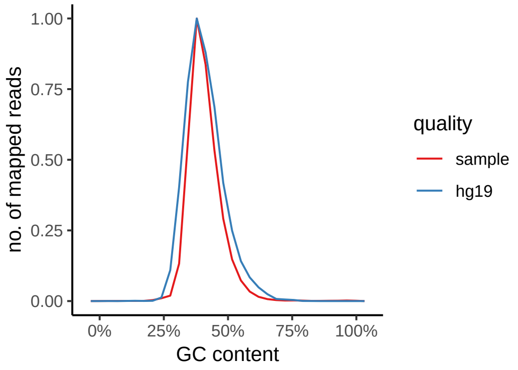

Copy number profile

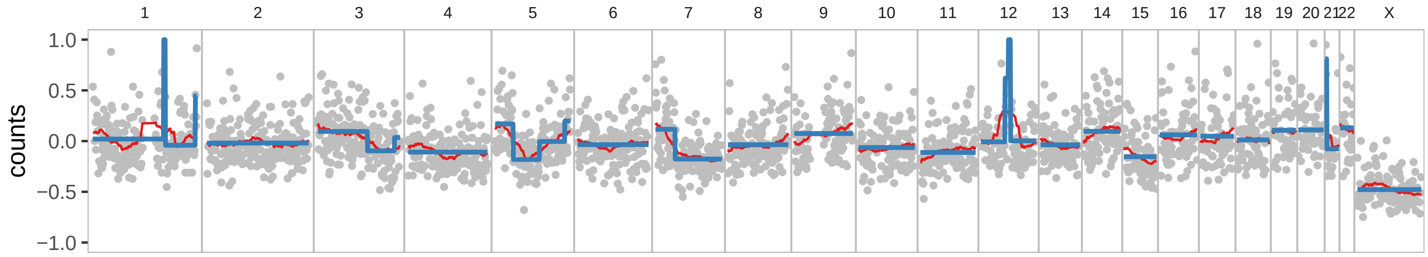

Methylation based classification

Methylation-based classification is based on **97587** CpG sites (overlap of sites covered in this sample and the training set). The out-of-bag error of this ad-hoc classifier is **2%**. Using this classifier, the sample has been classified as **well- / dedifferentiated liposarcoma**. This prediction has a confidence score of **0.09**. The raw and calibrated score distribution for the Top 10 entities is given below.

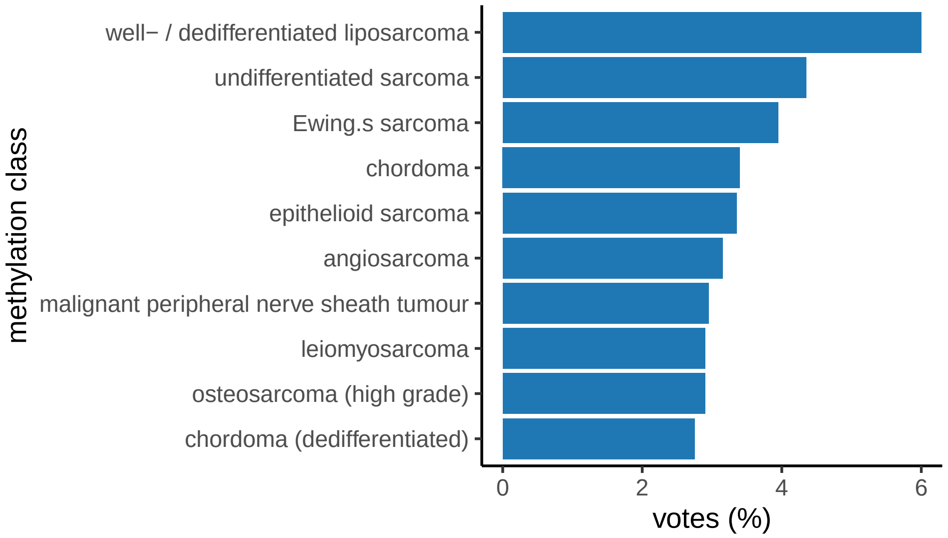

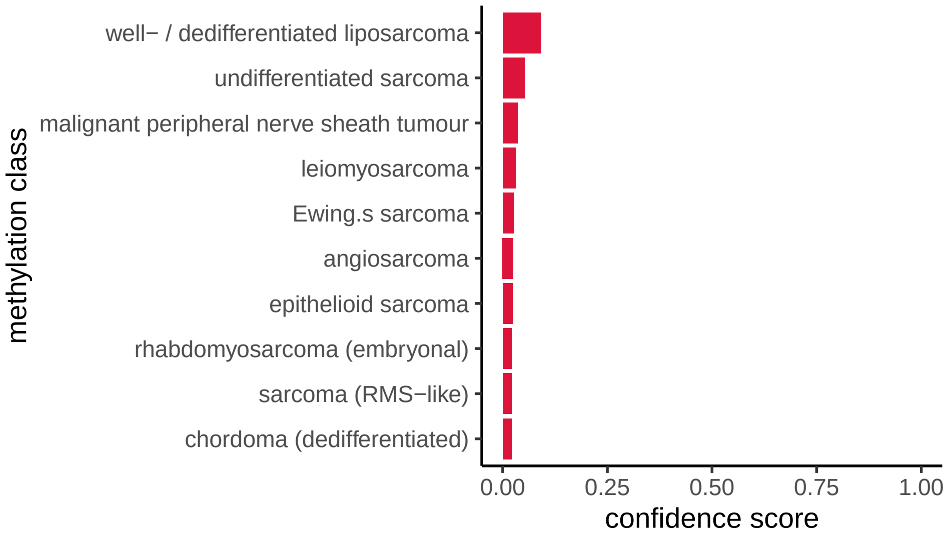

t-SNE plot

t-SNE plots are meant for visual quality control, not classification, and must be interpreted with care.

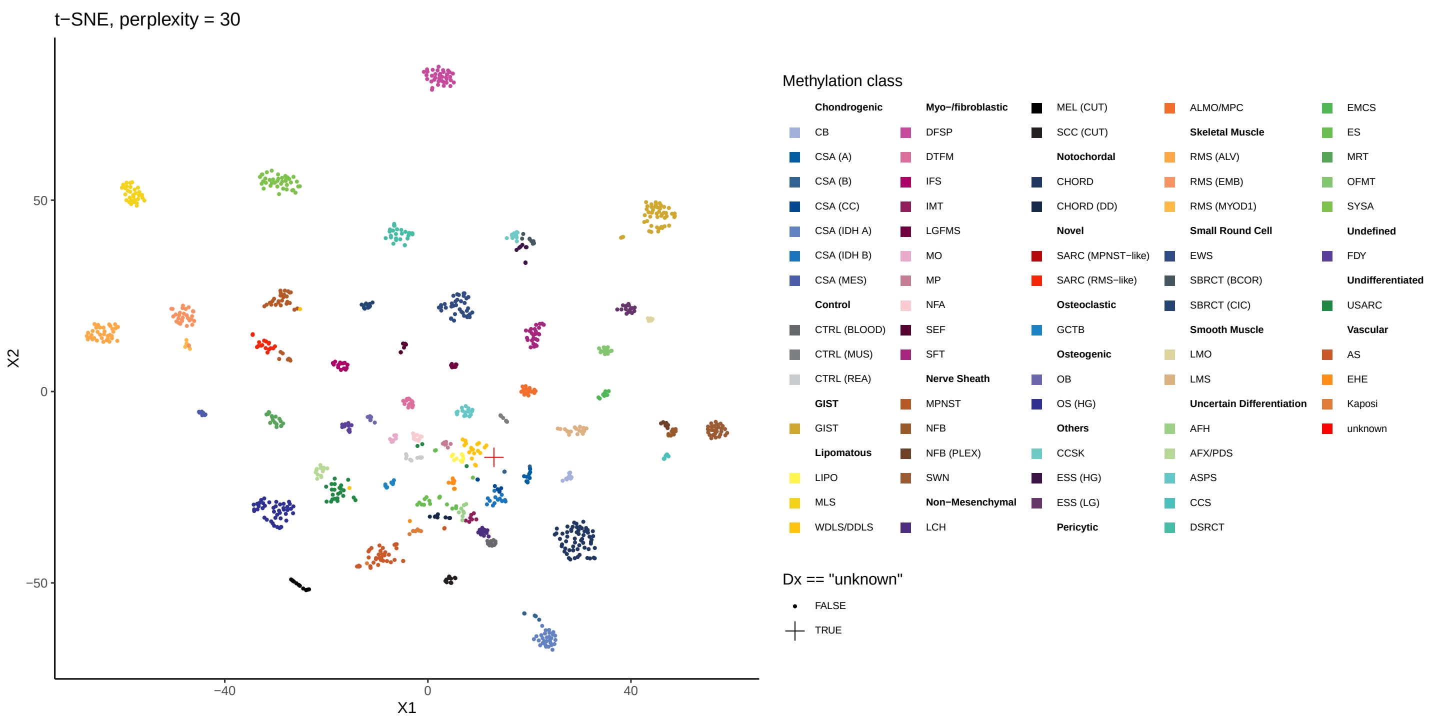

<u>**PATHOLOGY MICROSCOPIC**</u>

Dedifferentiated liposarcoma, low-grade dedifferentiated component.
The well differentiated component comprises most (90%) of the viable lesion and is of the sclerosing and inflammatory types.
There is extensive tumor necrosis comprising 60% of the lesion (post treatment?)
Surgical margins:

- The posterior surgical margin (sample 1) is involved by the lesion.
- The surgical margins through the mass (sample 2) are involved.
- Liver segment 7 (samples 3 and 4)- the liver capsule is involved by the tumor, but no parenchymal invasion was identified. The surgical margins through the liver are free.
- The paravertebral surgical margin (saample 5) is involved.
MDM2 innumostain - positive.

Fragments of liver tissue without significant pathological changes.

Nanopore sequencing report

SARC-02

Quality control metrics

Barcode statistics

```
Adapters detected in 10 of 3744800 reads
  RAB204/RAB214     10: |                      |   0.00 %
           none 3744778: |  ################### | 100.00 %
Barcodes detected in 10 of 3744800 adapters
           BC02      2: |                      |   0.00 %
           BC03      1: |                      |   0.00 %
           BC08      2: |                      |   0.00 %
           BC10      1: |                      |   0.00 %
           BC11      1: |                      |   0.00 %
           BC18      1: |                      |   0.00 %
           BC19      1: |                      |   0.00 %
           BC21      1: |                      |   0.00 %
           none 3744778: |  ################### | 100.00 %
12 reads were skipped due to the min. length filter.
Demultiplexing finished in 4317.50s
```

Read statistics

```
General summary:
Average percent identity:             91.7
Fraction of bases aligned:             1.0
Mean read length:                  5,037.7
Mean read quality:                    12.4
Median percent identity:              93.4
Median read length:                3,054.0
Median read quality:                  12.5
Number of reads:               4,033,554.0
Read length N50:                   9,628.0
STDEV read length:                 5,336.9
Total bases:              20,319,787,102.0
Total bases aligned:      19,590,566,843.0
Number, percentage and megabases of reads above quality cutoffs
>Q5:    4032608 (100.0%) 20319.7Mb
```

Mean genome coverage is 6.40X.

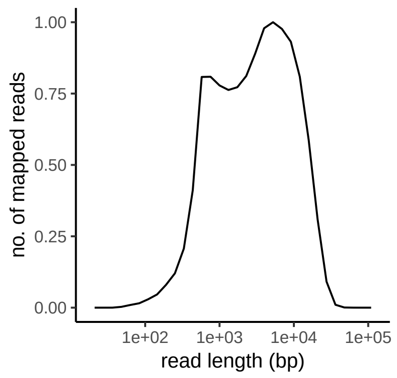

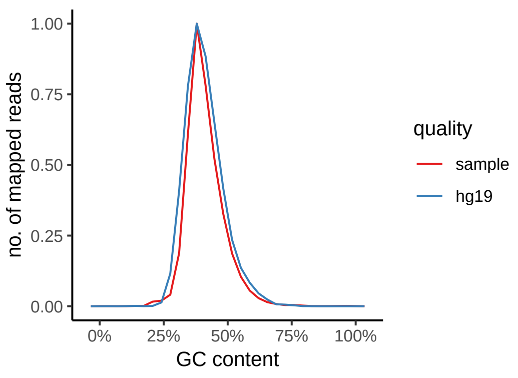

Copy number profile

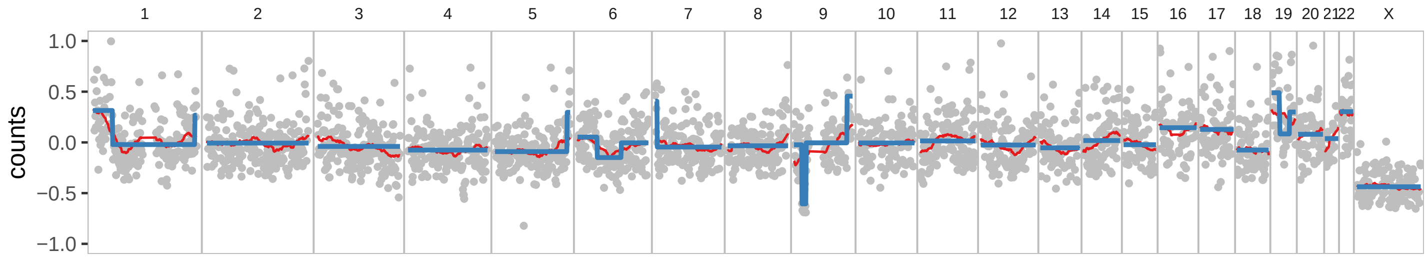

Methylation based classification

Methylation-based classification is based on **100000** CpG sites (overlap of sites covered in this sample and the training set). The out-of-bag error of this ad-hoc classifier is **1.9%**. Using this classifier, the sample has been classified as **myxoid liposarcoma**. This prediction has a confidence score of **0.3**. The raw and calibrated score distribution for the Top 10 entities is given below.

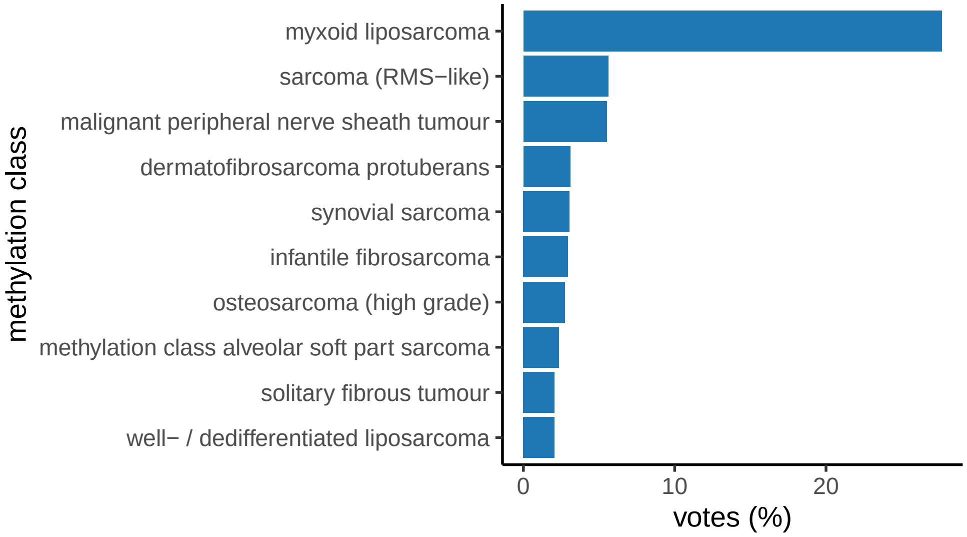

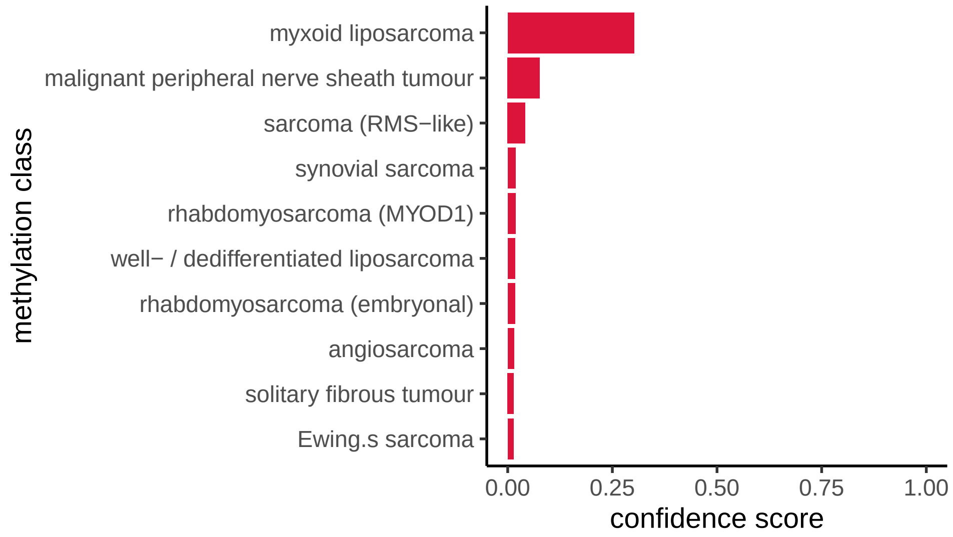

t-SNE plot

t-SNE plots are meant for visual quality control, not classification, and must be interpreted with care.

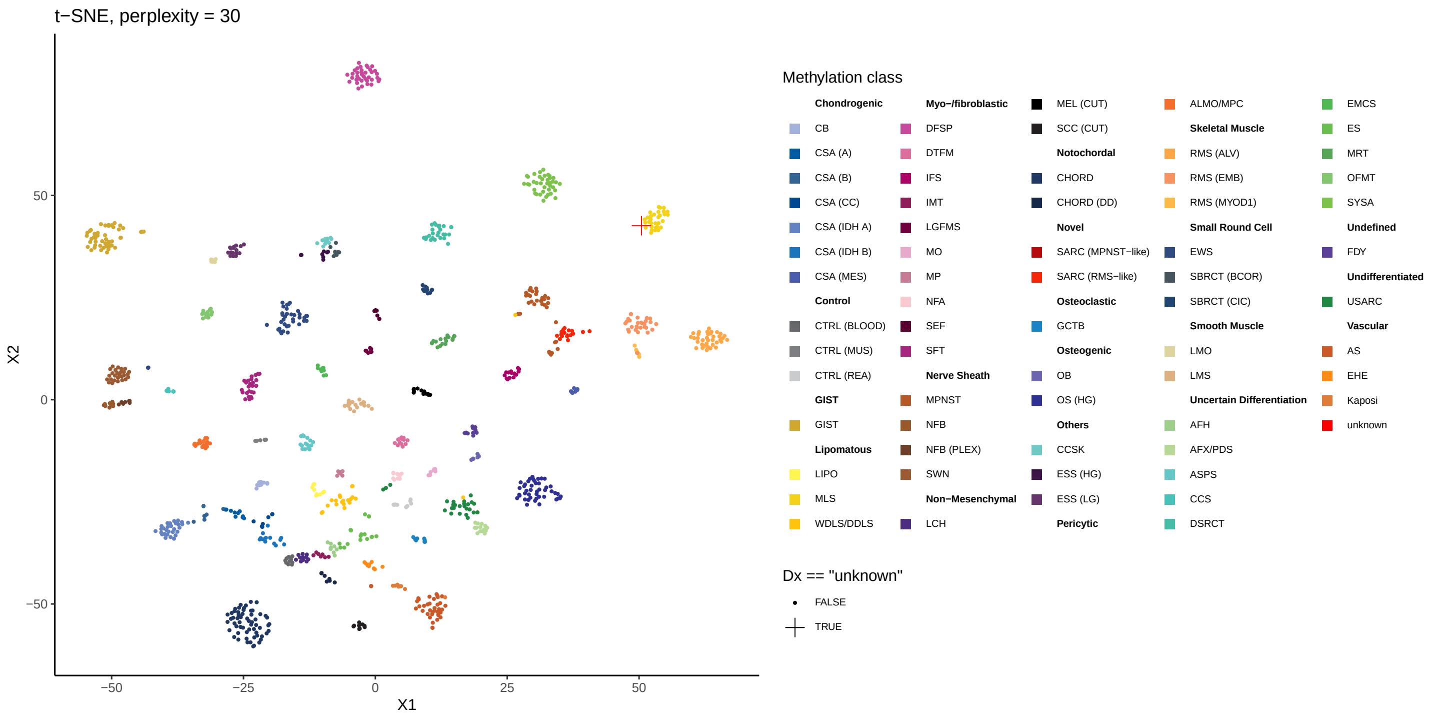

<u>**PATHOLOGY MICROSCOPIC**</u>

Lung, wedge resection:

- Metastatic myxoid liposarcoma. Round cell component <5%.

Nanopore sequencing report

SARC-03

Quality control metrics

Barcode statistics

```
Adapters detected in 4 of 909377 reads
  RAB204/RAB214      4: |                     |   0.00 %
           none 909370: |  ################## | 100.00 %
Barcodes detected in 4 of 909377 adapters
           BC02      1: |                     |   0.00 %
           BC06      1: |                     |   0.00 %
           BC12      1: |                     |   0.00 %
           BC13      1: |                     |   0.00 %
           none 909370: |  ################## | 100.00 %
3 reads were skipped due to the min. length filter.
Demultiplexing finished in 1067.95s
```

Read statistics

```
General summary:
Average percent identity:             92.1
Fraction of bases aligned:             0.9
Mean read length:                  5,678.9
Mean read quality:                    12.2
Median percent identity:              94.0
Median read length:                3,098.0
Median read quality:                  12.6
Number of reads:               1,082,033.0
Read length N50:                  11,191.0
STDEV read length:                 6,802.0
Total bases:               6,144,731,716.0
Total bases aligned:       5,440,923,675.0
Number, percentage and megabases of reads above quality cutoffs
>Q5:    1081285 (99.9%) 6144.6Mb
```

Mean genome coverage is 1.78X.

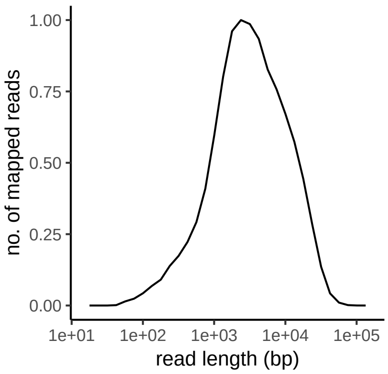

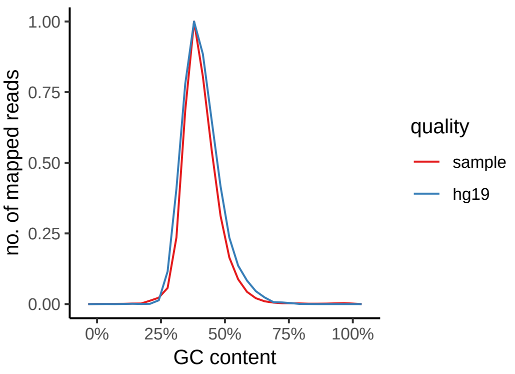

Copy number profile

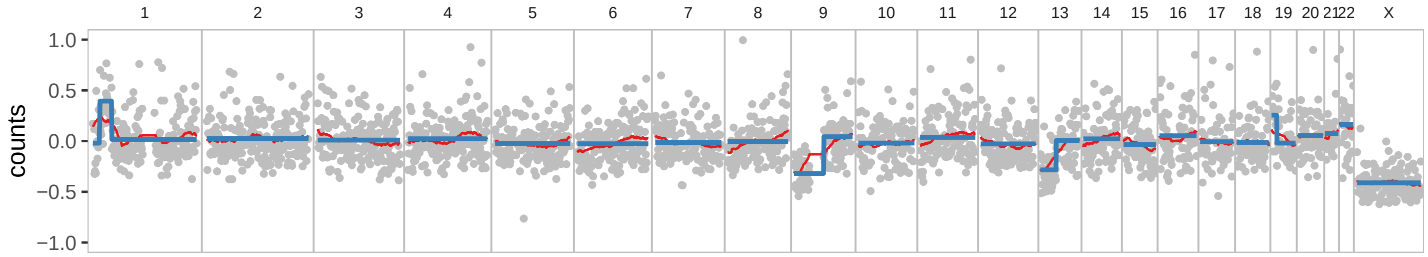

Methylation based classification

Methylation-based classification is based on **100000** CpG sites (overlap of sites covered in this sample and the training set). The out-of-bag error of this ad-hoc classifier is **1.4%.** Using this classifier, the sample has been classified as **leiomyosarcoma**. This prediction has a confidence score of **0.21**. The raw and calibrated score distribution for the Top 10 entities is given below.

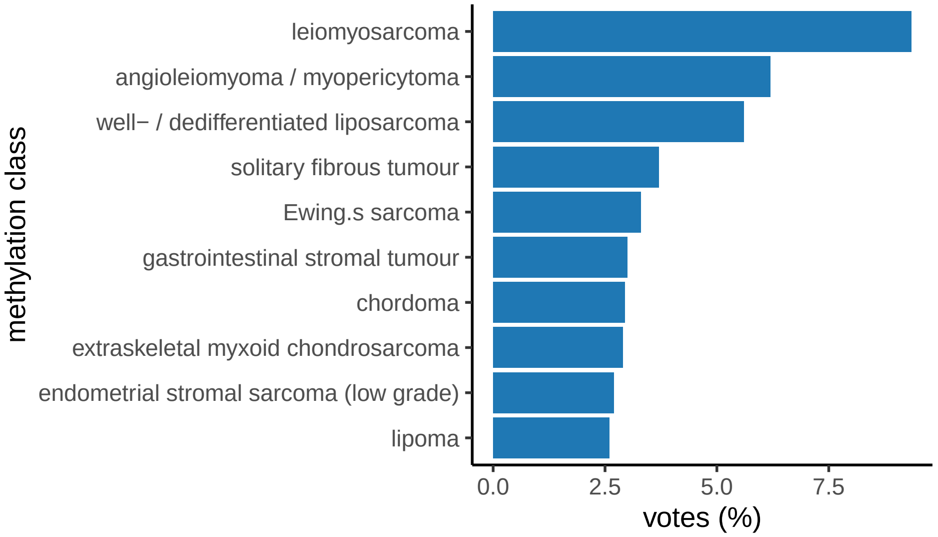

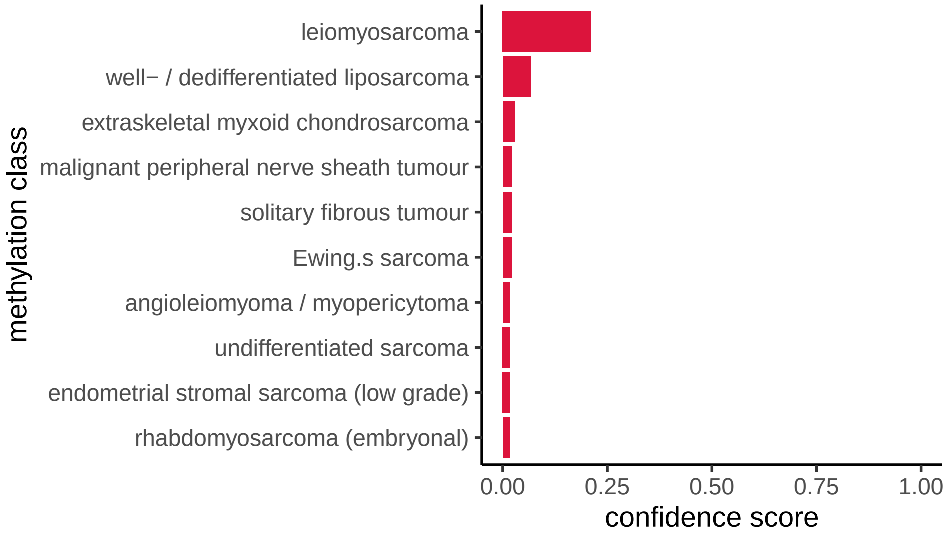

t-SNE plot

t-SNE plots are meant for visual quality control, not classification, and must be interpreted with care.

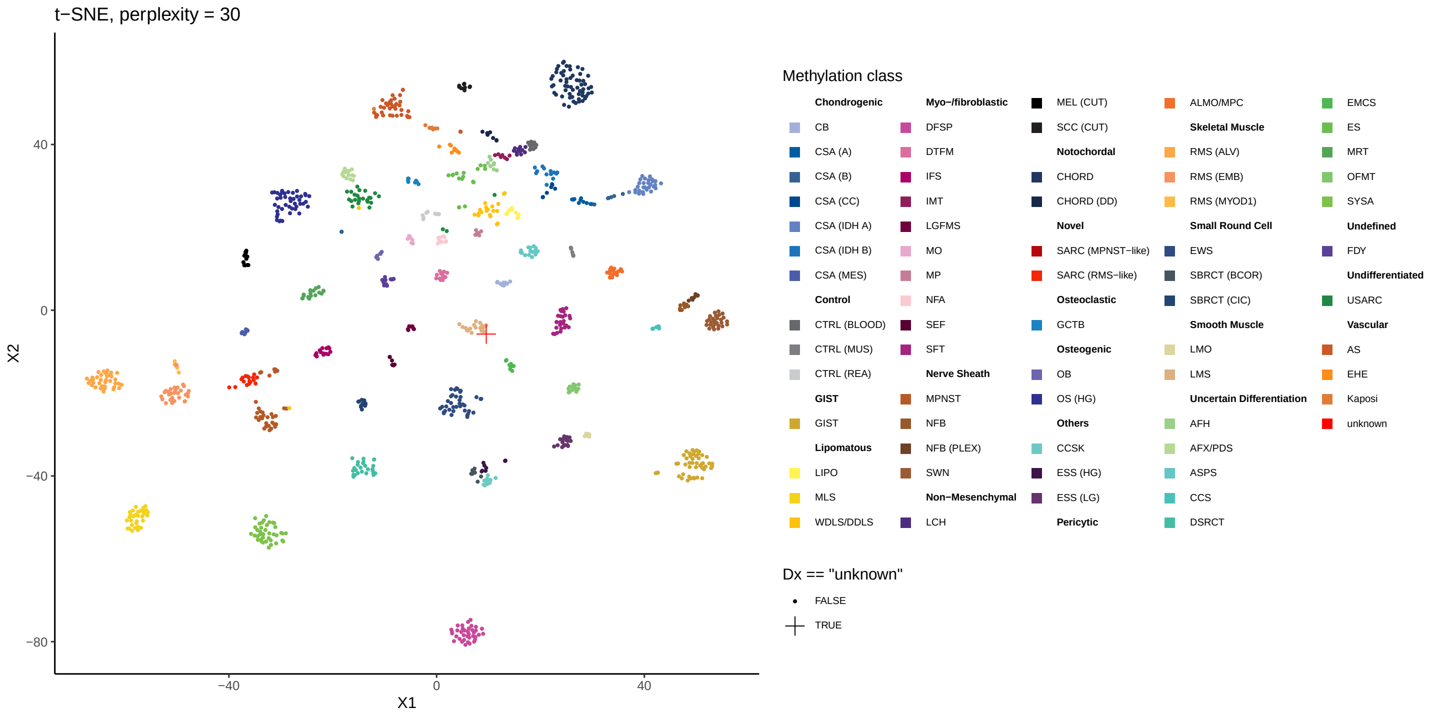

PATHOLOGY MICROSCOPIC

Soft tissue, thigh, left, proximal, needle biopsy:
Leiomyosarcoma, FNCLCC grade 2.
Severe atypia and pleomorphism are present.
Single cell necrosis identified.
Mitoses - 2/10 HPF.
Immunostains - actin and desmin positive.

Nanopore sequencing report

SARC-04

Quality control metrics

Barcode statistics

```
Adapters detected in 130656 of 131664 reads
         RBK004 130656: |  ################### |  99.23 %
           none   1007: |                      |   0.76 %
Barcodes detected in 130656 of 131664 adapters
      barcode01 130656: |  ################### |  99.23 %
           none   1007: |                      |   0.76 %
1 reads were skipped due to the min. length filter.
Demultiplexing finished in 72.48s
```

Read statistics

```
General summary:
Average percent identity:          93.4
Fraction of bases aligned:           0.9
Mean read length:               1,956.6
Mean read quality:                 12.5
Median percent identity:           94.4
Median read length:               961.0
Median read quality:               12.6
Number of reads:              126,977.0
Read length N50:                4,032.0
STDEV read length:              2,462.6
Total bases:              248,437,551.0
Total bases aligned:      231,679,879.0
Number, percentage and megabases of reads above quality cutoffs
>Q5:    126959 (100.0%) 248.4Mb
```

Mean genome coverage is 0.08X.

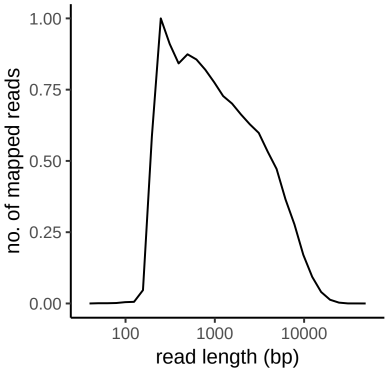

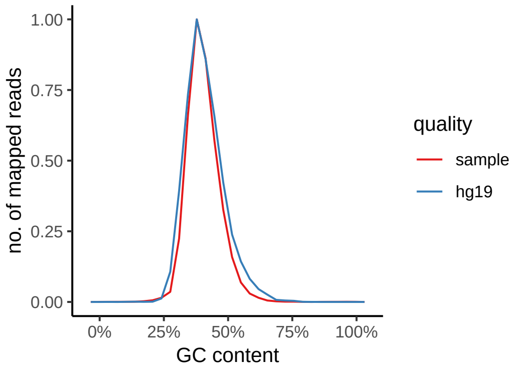

Copy number profile

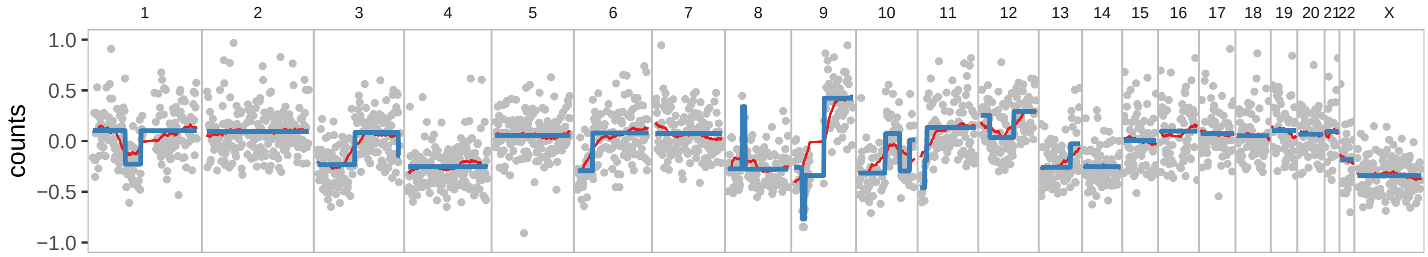

Methylation based classification

Methylation-based classification is based on **18136** CpG sites (overlap of sites covered in this sample and the training set). The out-of-bag error of this ad-hoc classifier is **2.1%**. Using this classifier, the sample has been classified as **undifferentiated sarcoma**. This prediction has a confidence score of **0.08**. The raw and calibrated score distribution for the Top 10 entities is given below.

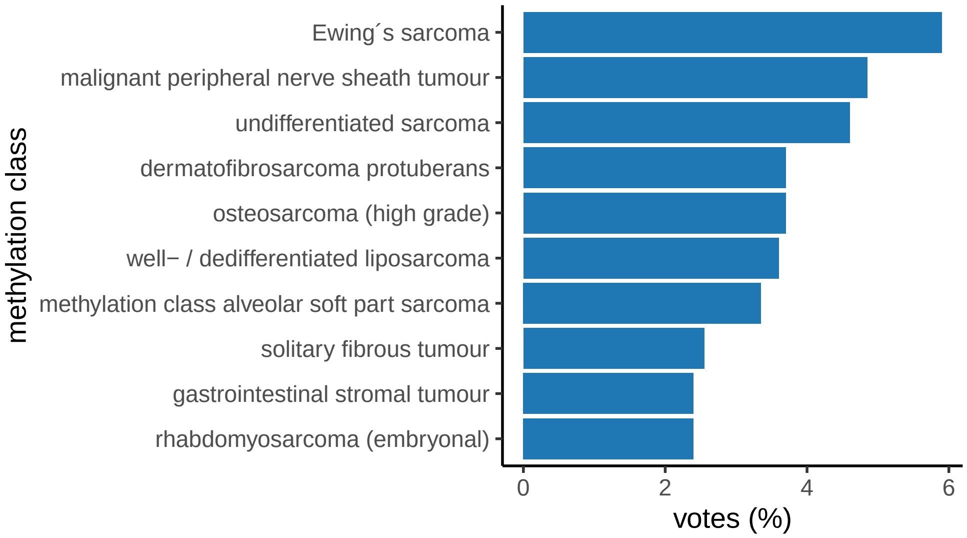

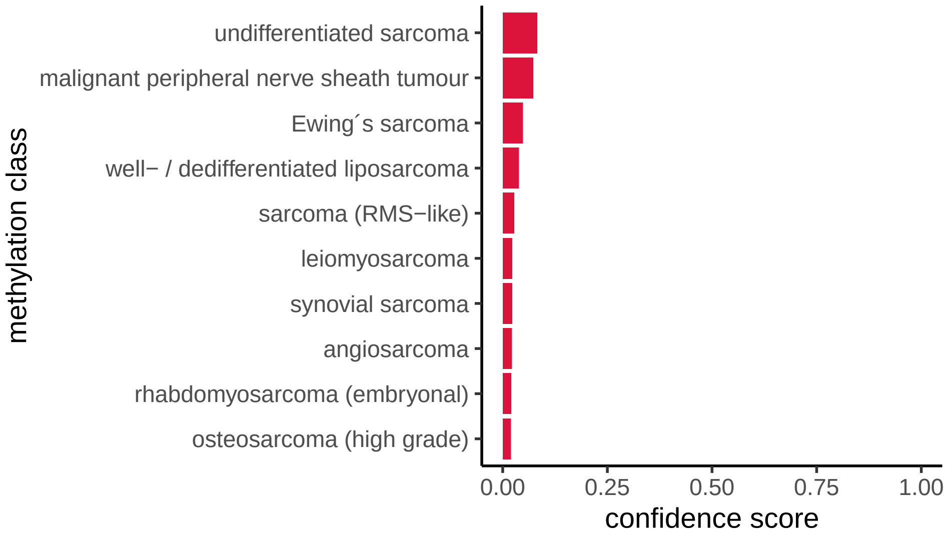

t-SNE plot

t-SNE plots are meant for visual quality control, not classification, and must be interpreted with care.

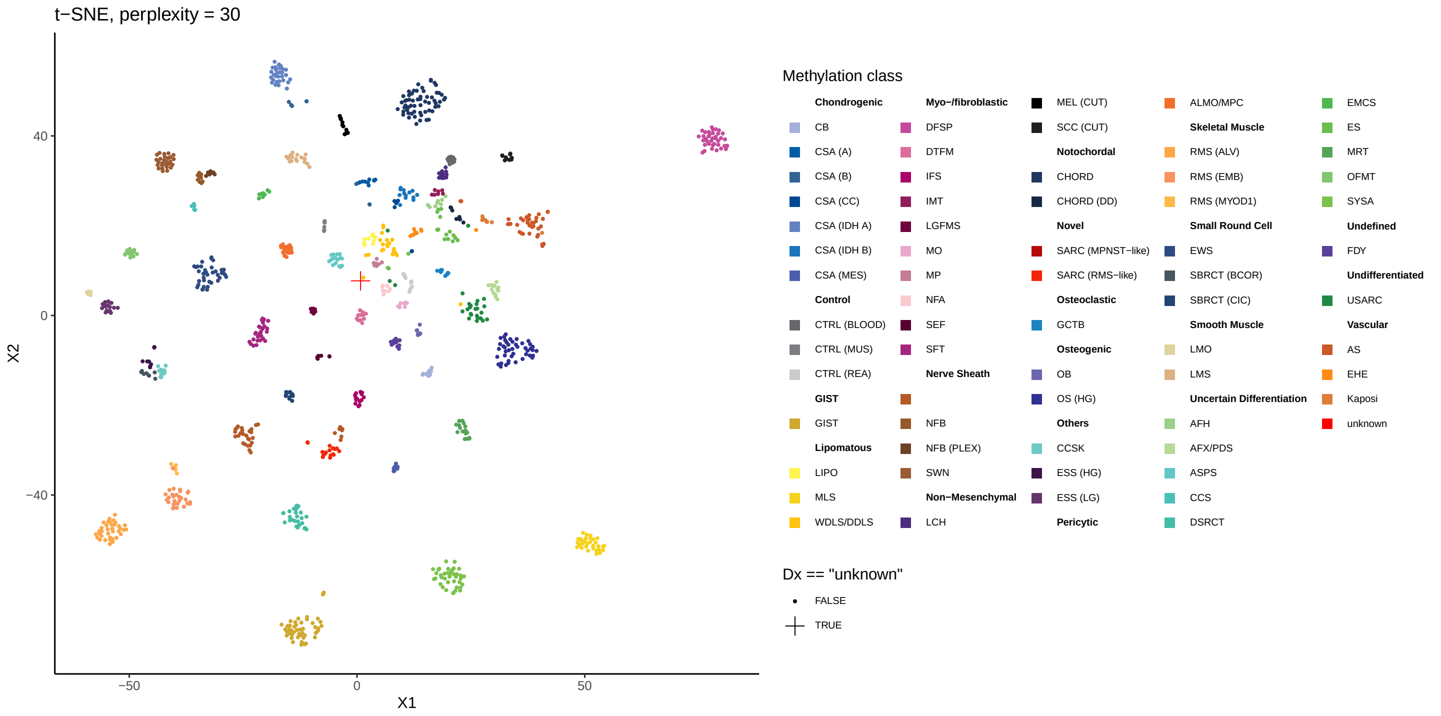

PATHOLOGY MICROSCOPIC

Left leg, disarticulation at the hip:

- Undifferentiated small round spindle cell sarcoma.

- The tumor is 32cm in diameter; lymphovascular invasion is not observed.

- Necrosis > 50%.

- Mitoses: 31/10 HPF.

- Numerous thrombosed vascular spaces are present within the lesion.

- The proximal margins (muscle, soft tissue, vessels and cartilage) are free.

(consistent)and Fli-1 (weak diffuse) positive, while negative for MUC4, CD31, Chromogranin, S100 and CD68.

MPNST

Note: The immunophenotype of the tumor probably presents the dedifferentiation post chemotherapy treatment.

Fluorescence in situ hybridization analysis of the FUS gene test result (evaluated by Prof. Eli Pikarsky):

In most of the nuclei of the tumor cells (>95%) the probe signals appear as two adjacent signals or fused yellow, which is interpreted as no evidence for split signals .
This result suggests that there is no indication for translocation associated with the FUS gene in the specimen.Notes:

Nanopore sequencing report

SARC-05

Quality control metrics

Barcode statistics

```
Adapters detected in 79943 of 80451 reads
         RBK004  79943: |  ################### |  99.37 %
           none    504: |                      |   0.63 %
Barcodes detected in 79943 of 80451 adapters
      barcode02  79943: |  ################### |  99.37 %
           none    504: |                      |   0.63 %
4 reads were skipped due to the min. length filter.
Demultiplexing finished in 59.18s
```

Read statistics

```
General summary:
Average percent identity:           92.9
Fraction of bases aligned:            1.0
Mean read length:                4,537.0
Mean read quality:                  12.5
Median percent identity:            94.1
Median read length:              1,658.5
Median read quality:                12.6
Number of reads:                78,072.0
Read length N50:                11,579.0
STDEV read length:               6,505.7
Total bases:               354,211,164.0
Total bases aligned:       340,155,607.0
Number, percentage and megabases of reads above quality cutoffs
>Q5:    78053 (100.0%) 354.2Mb
```

Mean genome coverage is 0.11X.

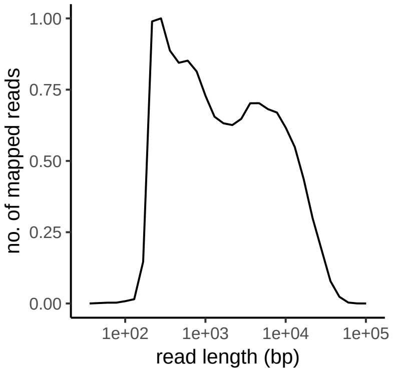

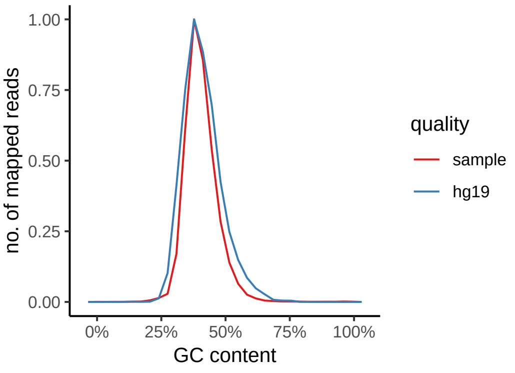

Copy number profile

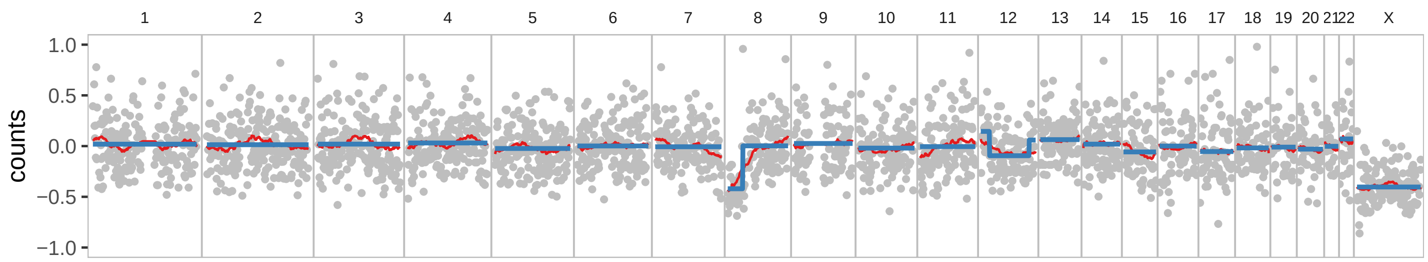

Methylation based classification

Methylation-based classification is based on **26439** CpG sites (overlap of sites covered in this sample and the training set). The out-of-bag error of this ad-hoc classifier is **2.3%**. Using this classifier, the sample has been classified as **extraskeletal myxoid chondrosarcoma**. This prediction has a confidence score of **0.88**. The raw and calibrated score distribution for the Top 10 entities is given below.

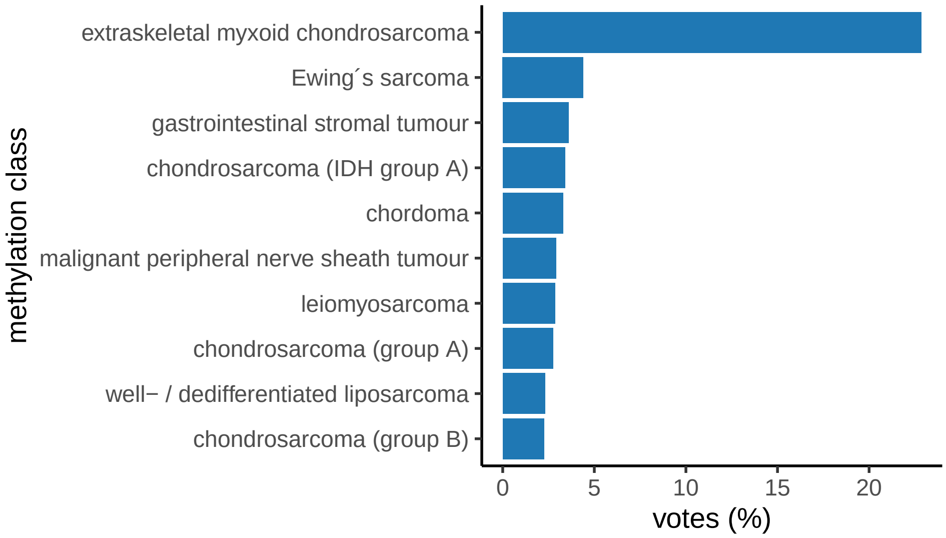

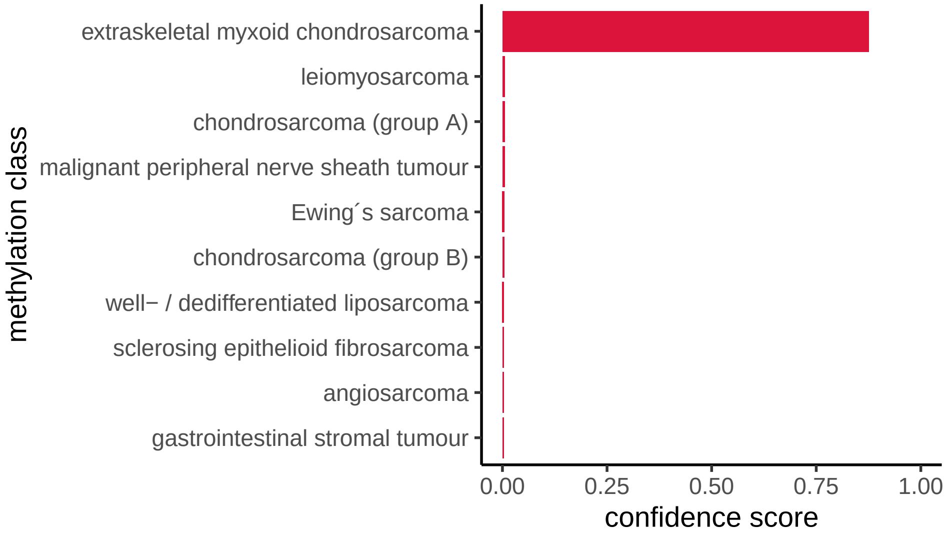

t-SNE plot

t-SNE plots are meant for visual quality control, not classification, and must be interpreted with care.

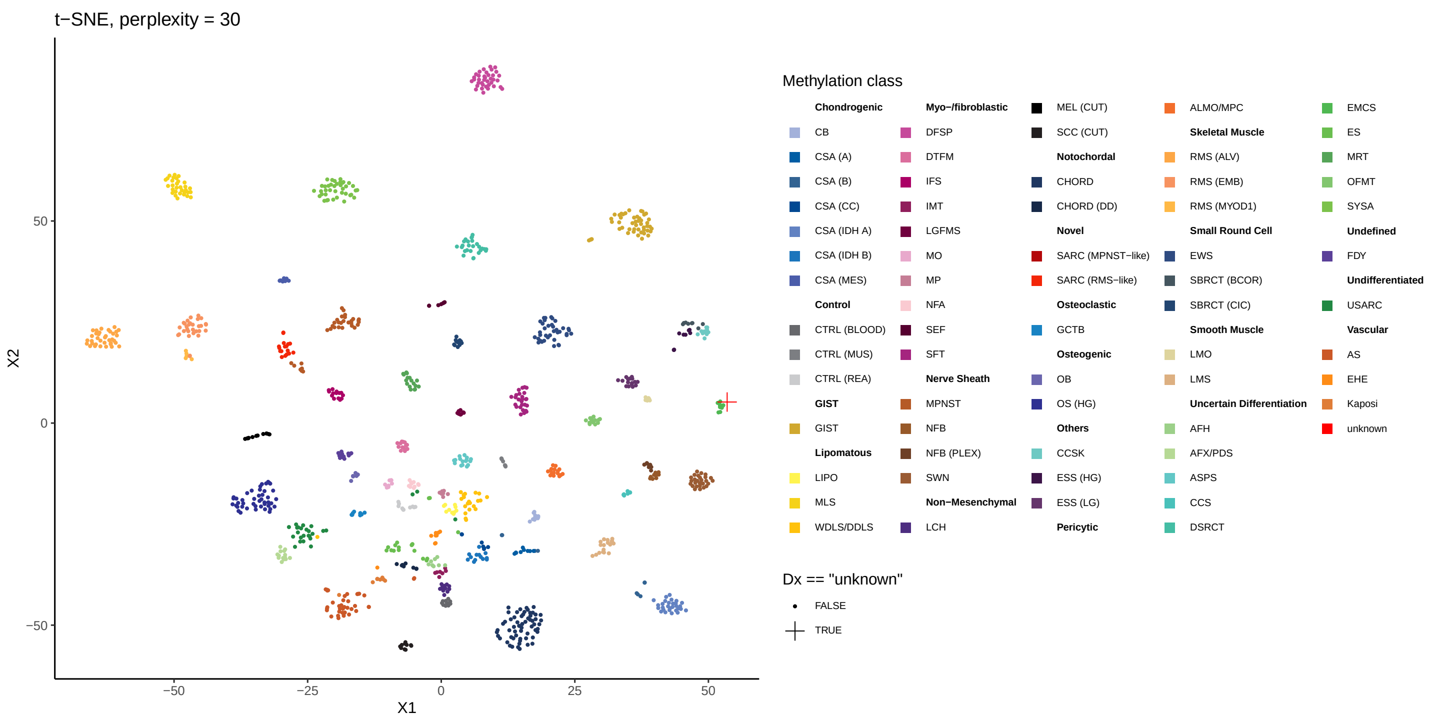

PATHOLOGY MICROSCOPIC

LT leg; Above knee amputation:
Extraskeletal myxoid chondrosarcoma.
The lesion involves massively the lower half of the leg.
The surgical margins through the soft tissue are free.

Nanopore sequencing report

SARC-06

Quality control metrics

Barcode statistics

```
Adapters detected in 150416 of 151731 reads
         RBK004 150416: |  ################### |  99.13 %
           none   1313: |                      |   0.87 %
Barcodes detected in 150416 of 151731 adapters
      barcode03 150416: |  ################### |  99.13 %
           none   1313: |                      |   0.87 %
2 reads were skipped due to the min. length filter.
Demultiplexing finished in 82.63s
```

Read statistics

```
General summary:
Average percent identity:            93.4
Fraction of bases aligned:             0.9
Mean read length:                 1,310.3
Mean read quality:                   11.9
Median percent identity:             94.2
Median read length:                 419.0
Median read quality:                 11.9
Number of reads:                134,682.0
Read length N50:                  4,927.0
STDEV read length:                2,828.7
Total bases:                176,470,678.0
Total bases aligned:        159,273,513.0
Number, percentage and megabases of reads above quality cutoffs
>Q5:    134670 (100.0%) 176.5Mb
```

Mean genome coverage is 0.05X.

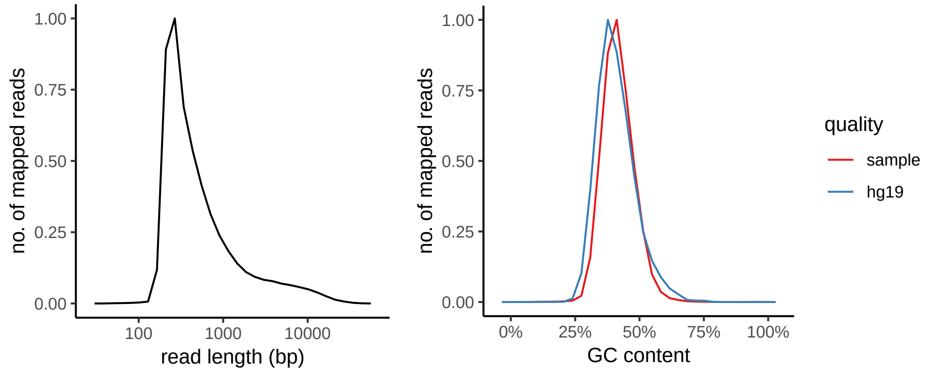

Copy number profile

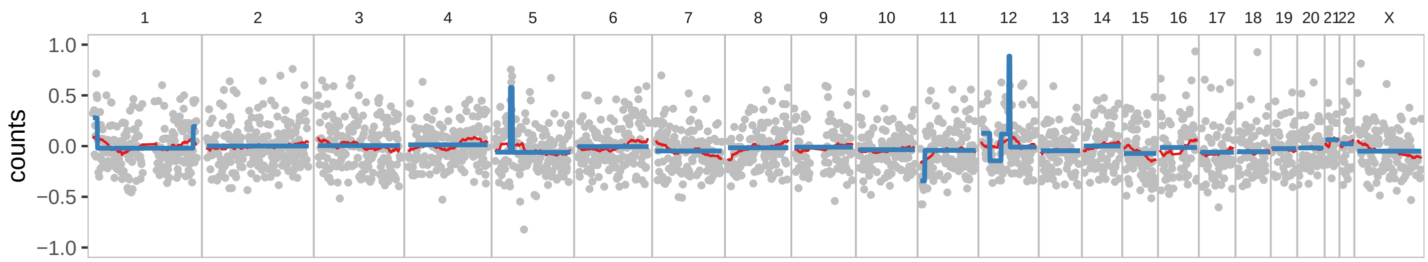

Methylation based classification

Methylation-based classification is based on **11564** CpG sites (overlap of sites covered in this sample and the training set). The out-of-bag error of this ad-hoc classifier is **2%**. Using this classifier, the sample has been classified as **well- / dedifferentiated liposarcoma**. This prediction has a confidence score of **0.08**. The raw and calibrated score distribution for the Top 10 entities is given below.

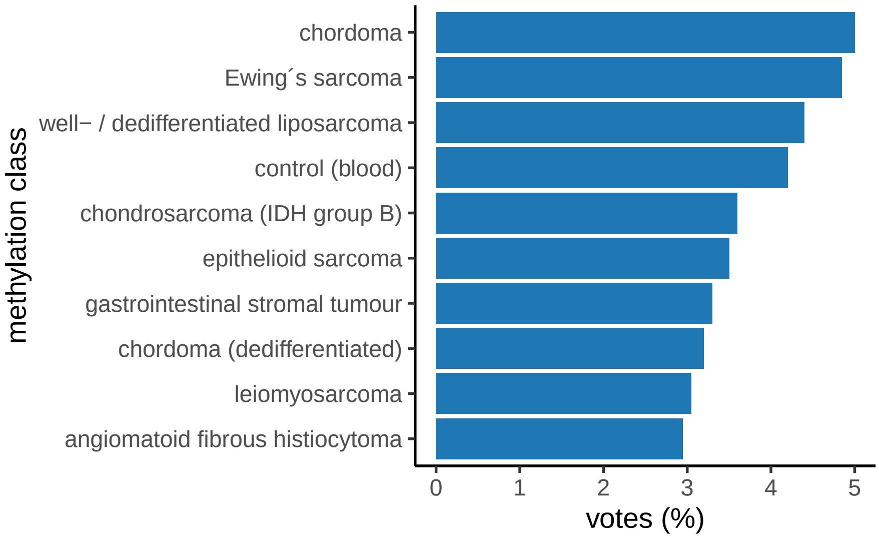

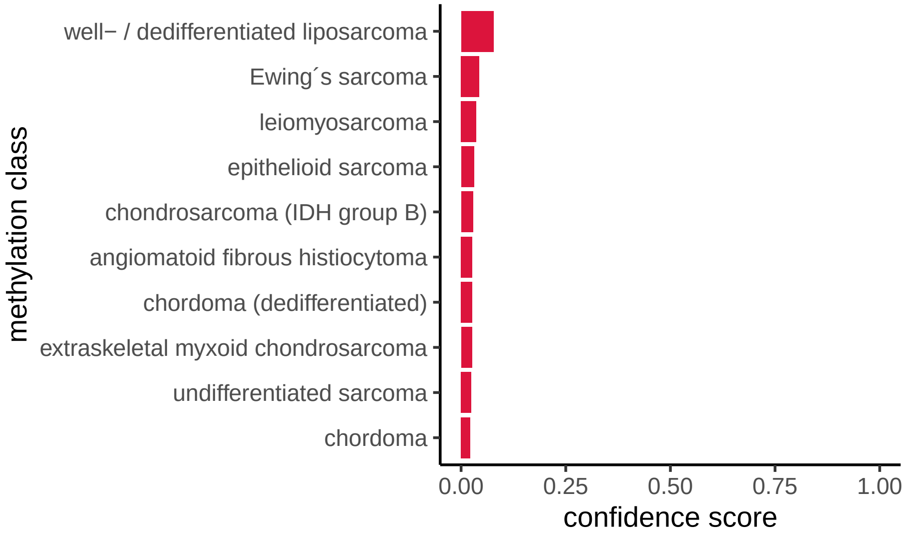

t-SNE plot

t-SNE plots are meant for visual quality control, not classification, and must be interpreted with care.

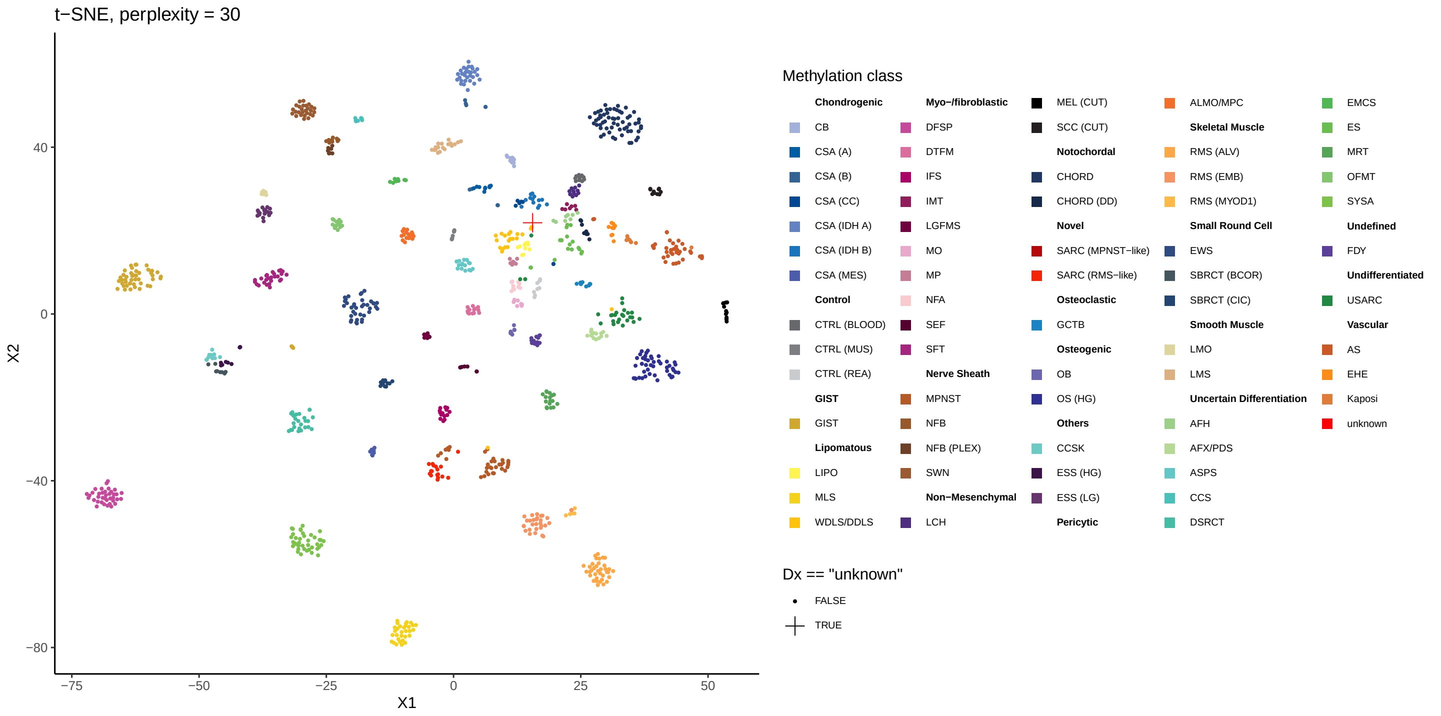

PATHOLOGY MICROSCOPIC

Abdominal mass, laparotomy:

Dedifferentiated liposarcoma, FNCLCC G3.
The tumor demonstrates relatively well differentiated areas adjacent to myxoid foci, and dedifferentiated pleomorphic areas.
There is extensive necrosis.
MDM2 immunostain was positive (performed on selected sections).
The tumor involves pericolic, peripancreatic and perirenal fat, as well as perisplenic tissue, with very focal superficial invasion of the pancreas and kidney. A small accessory spleen is identified, free of tumor.
Tumor is seen on the inked surgical margins.
A reactive lymph node is seen in the peripancreatic fat.

Nanopore sequencing report

SARC-07

Quality control metrics

Barcode statistics

```
Adapters detected in 96771 of 97940 reads
         RBK004  96771: |  ################### |  98.81 %
           none   1168: |                      |   1.19 %
Barcodes detected in 96771 of 97940 adapters
      barcode04  96771: |  ################### |  98.81 %
           none   1168: |                      |   1.19 %
1 reads were skipped due to the min. length filter.
Demultiplexing finished in 60.73s
```

Read statistics

```
General summary:
Average percent identity:          92.8
Fraction of bases aligned:          0.9
Mean read length:               2,830.3
Mean read quality:                 12.1
Median percent identity:           93.8
Median read length:               901.0
Median read quality:               12.2
Number of reads:               94,770.0
Read length N50:                8,466.0
STDEV read length:              4,890.2
Total bases:              268,228,073.0
Total bases aligned:      254,049,593.0
Number, percentage and megabases of reads above quality cutoffs
>Q5:    94765 (100.0%) 268.2Mb
```

Mean genome coverage is 0.08X.

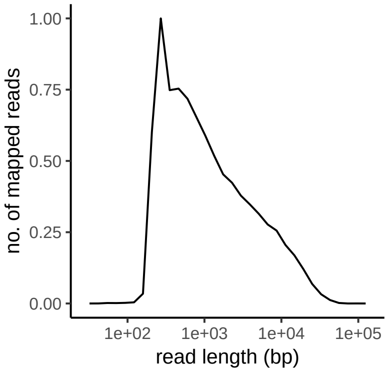

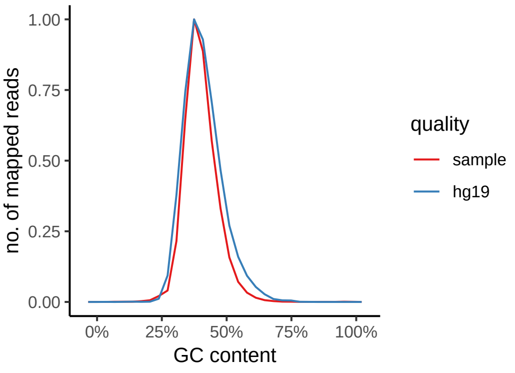

Copy number profile

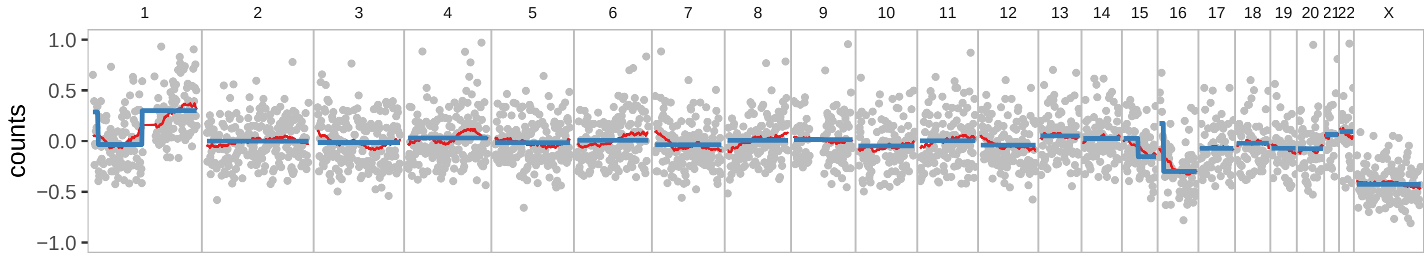

Methylation based classification

Methylation-based classification is based on **19304** CpG sites (overlap of sites covered in this sample and the training set). The out-of-bag error of this ad-hoc classifier is **2.3%**. Using this classifier, the sample has been classified as **Ewing´s sarcoma**. This prediction has a confidence score of **0.91**. The raw and calibrated score distribution for the Top 10 entities is given below.

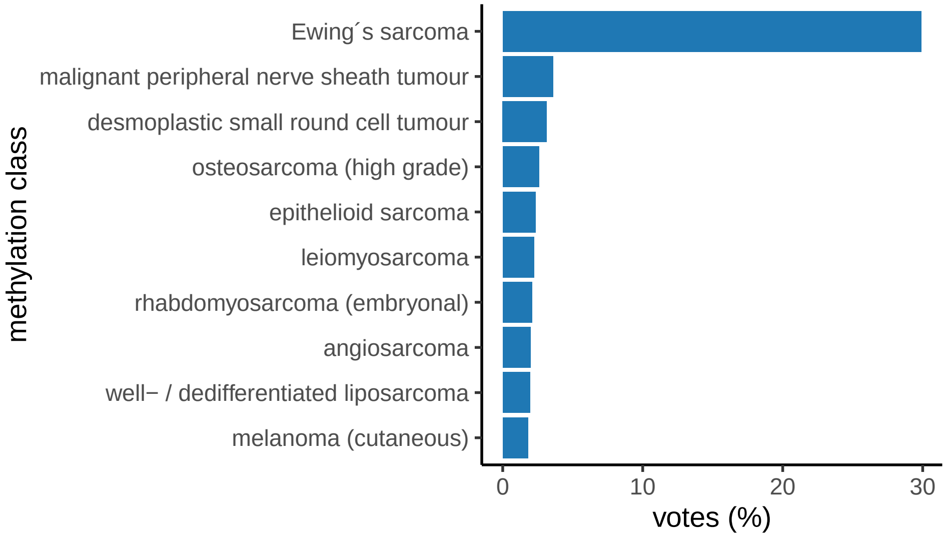

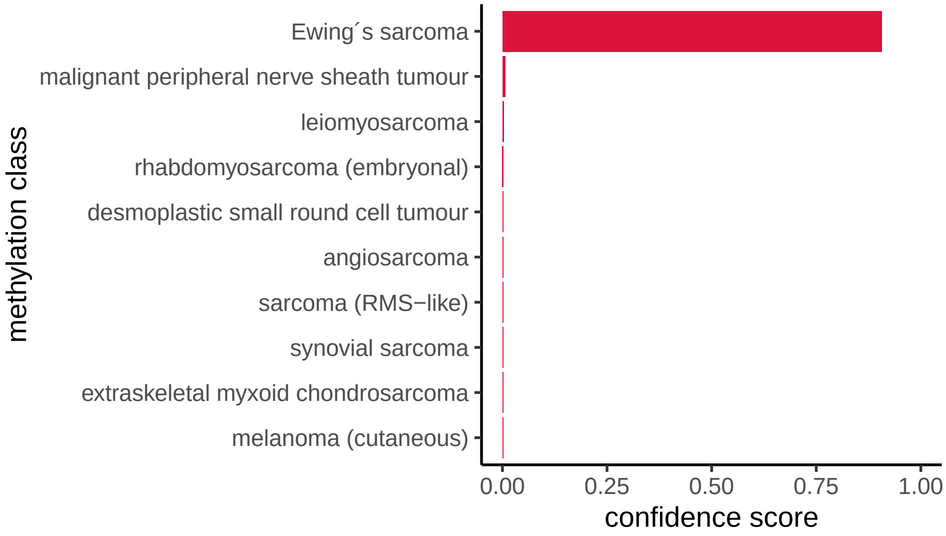

t-SNE plot

t-SNE plots are meant for visual quality control, not classification, and must be interpreted with care.

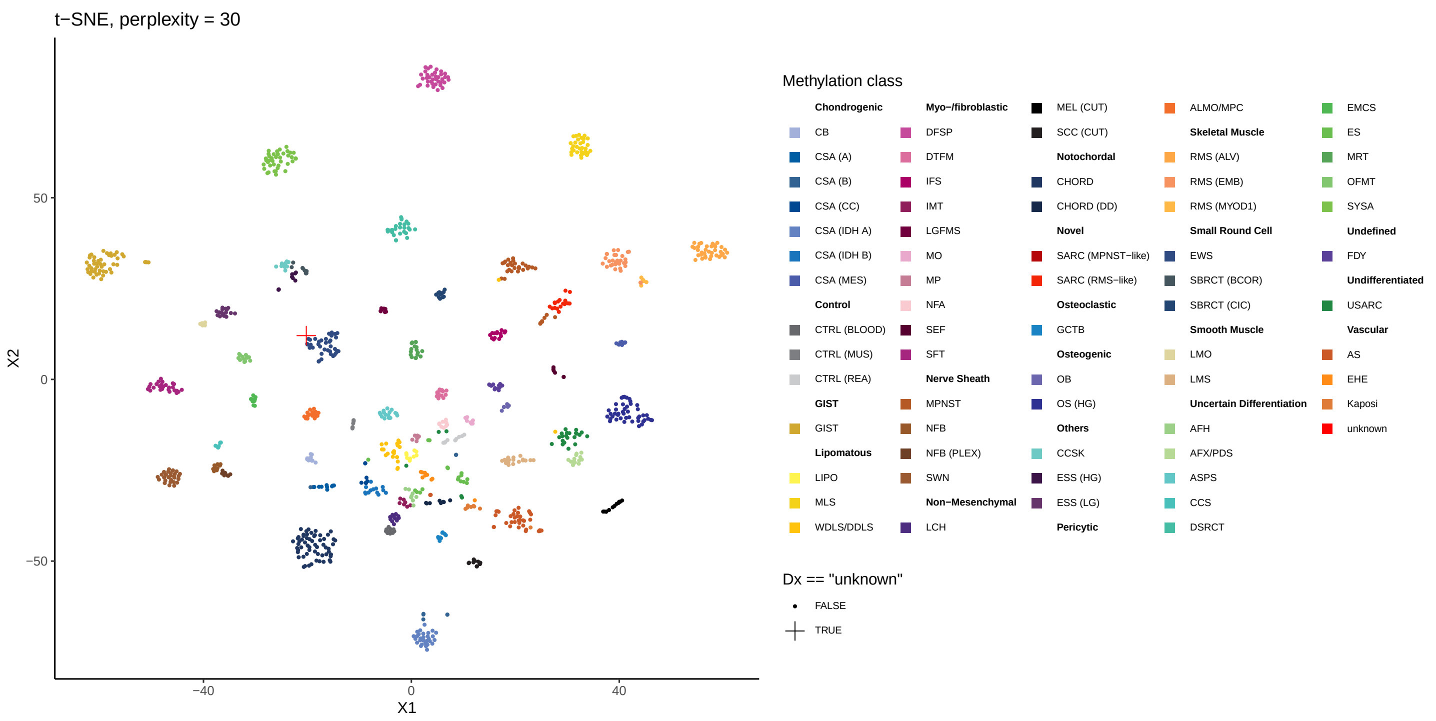

PATHOLOGY MICROSCOPIC

Small intestine, tumor resection, summary of findings: Unclassified spindle-round cell sarcoma.
See comment.

Comment:
Malignant hypercellular mesenchymal tumor composed of crowded spindle and small cells with indistinct cytoplasm and spindle to ovoid hyperchromatic nuclei. There are foci of fascicular arrangement, but most of the lesion does not demonstrate definite architecture. Numerous hemorrhagic lakes are present. Foci of necrosis comprising less than 5% of the lesion are seen. The mitotic rate is >50/50 HPF. Atypical mitoses are present.
The tumor is located in the peri-intestinal fat tissue, well circumscribed and almost completely surrounded by a fibrotic pseudocapsule; numerous foci of infiltration into the outer aspect of intestinal muscular wall are seen. Tumor measuring at least 6.5 cm; additional fragments of tumor tissue are also submitted.
Deposits of the tumor measuring 0.6 cm are noted in the additional samples and in the biopsy taken from omentum.
No vascular invasion seen.
Tumor present at the radial margin or resection.
The intestinal mucosa is free of tumor.
The resection margins through the intestine are free of tumor.

Immunohistochemical results:
Tumor cells demonstrate diffuse strong stain with CD99 and multifocal moderate to strong positive staining with TLE1, FLi1, NKX2.2, Bcl2 and c-kit. The immunostain with DOG1, CD34, Actin, Caldesmon, Calponin, Desmin, Myogenin, Calretinin, S100 protein, SOX10,AE1/AE3, CK19, CK7, EMA are negative.

Previous FISH examination for EWSR translocation was negative (201813164 EK)

The immunophenotype does not contribute to the diagnosis. See note.
Note:

- Rule out the possibility of high grade dedifferentiated gastrointestinal
- Poorly differentiated synovial sarcoma is also considered in a differential

Nanopore sequencing report

SARC-08

Quality control metrics

Barcode statistics

```
Adapters detected in 19519 of 19712 reads
         RBK004  19519: |  ################### |  99.02 %
           none    183: |                      |   0.93 %
Barcodes detected in 19519 of 19712 adapters
      barcode01  19519: |  ################### |  99.02 %
           none    183: |                      |   0.93 %
10 reads were skipped due to the min. length filter.
Demultiplexing finished in 13.94s
```

Read statistics

```
General summary:
Average percent identity:          92.0
Fraction of bases aligned:          1.0
Mean read length:                5,121.4
Mean read quality:                  12.2
Median percent identity:            93.4
Median read length:              2,723.5
Median read quality:                12.3
Number of reads:                17,300.0
Read length N50:                10,603.0
STDEV read length:               6,415.7
Total bases:                88,600,605.0
Total bases aligned:        85,129,026.0
Number, percentage and megabases of reads above quality cutoffs
>Q5:    17292 (100.0%) 88.6Mb
```

Mean genome coverage is 0.03X.

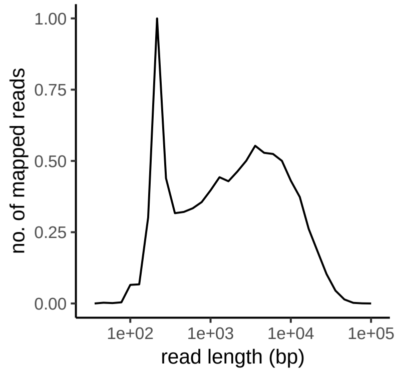

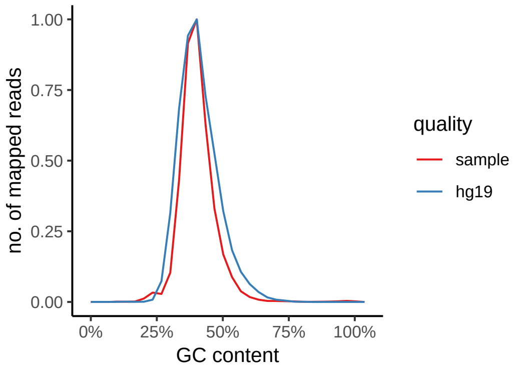

Copy number profile

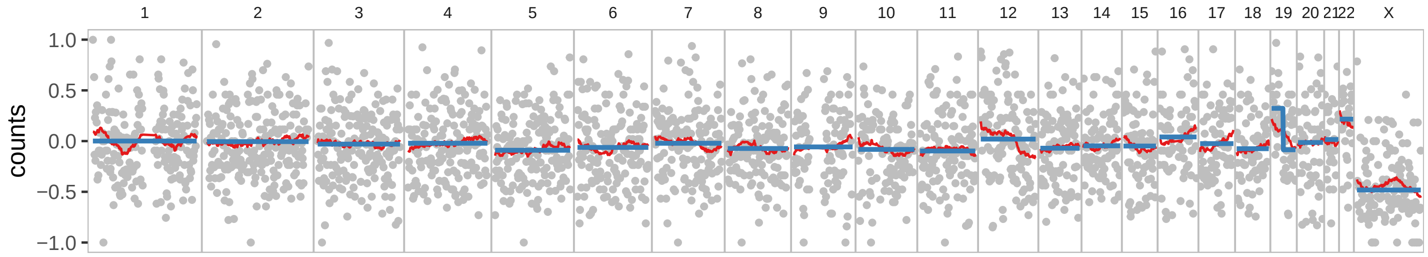

Methylation based classification

Methylation-based classification is based on **6539** CpG sites (overlap of sites covered in this sample and the training set). The out-of-bag error of this ad-hoc classifier is **2.4%**. Using this classifier, the sample has been classified as **well- / dedifferentiated liposarcoma**. This prediction has a confidence score of **0.1**. The raw and calibrated score distribution for the Top 10 entities is given below.

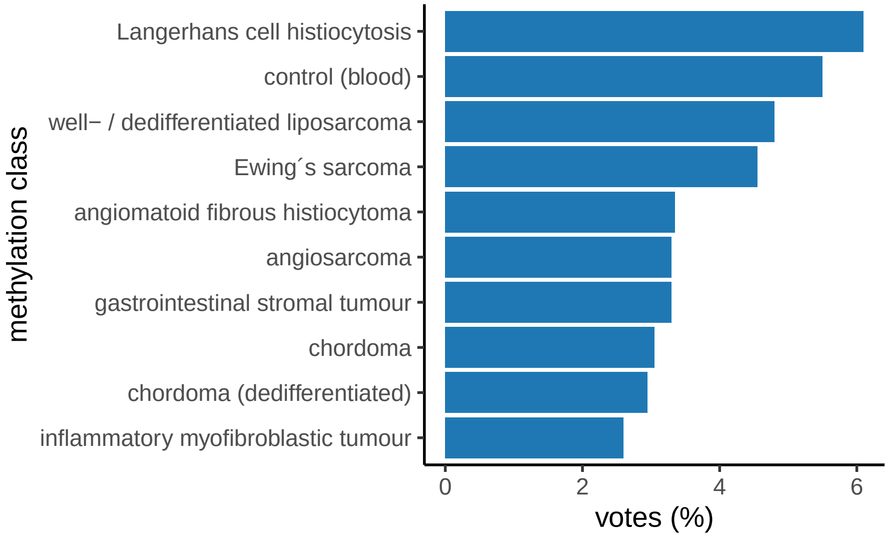

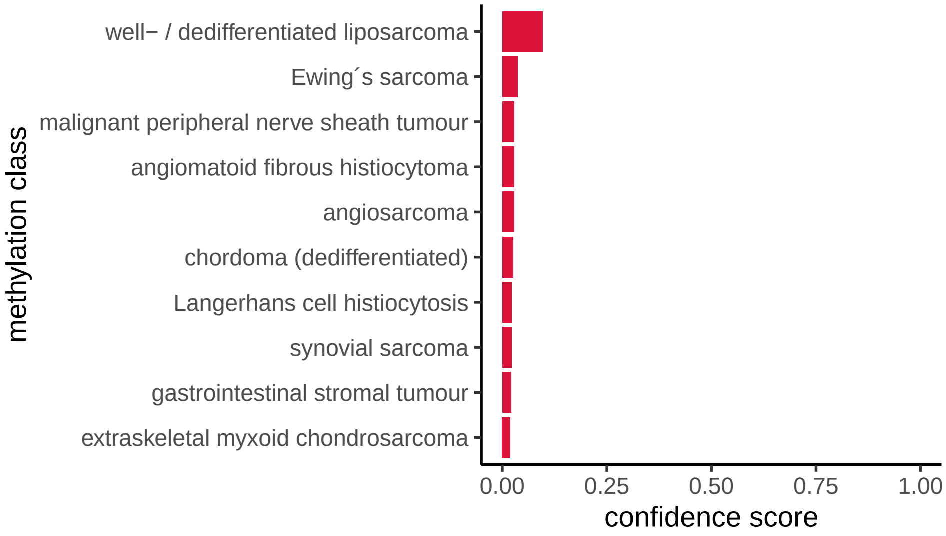

t-SNE plot

t-SNE plots are meant for visual quality control, not classification, and must be interpreted with care.

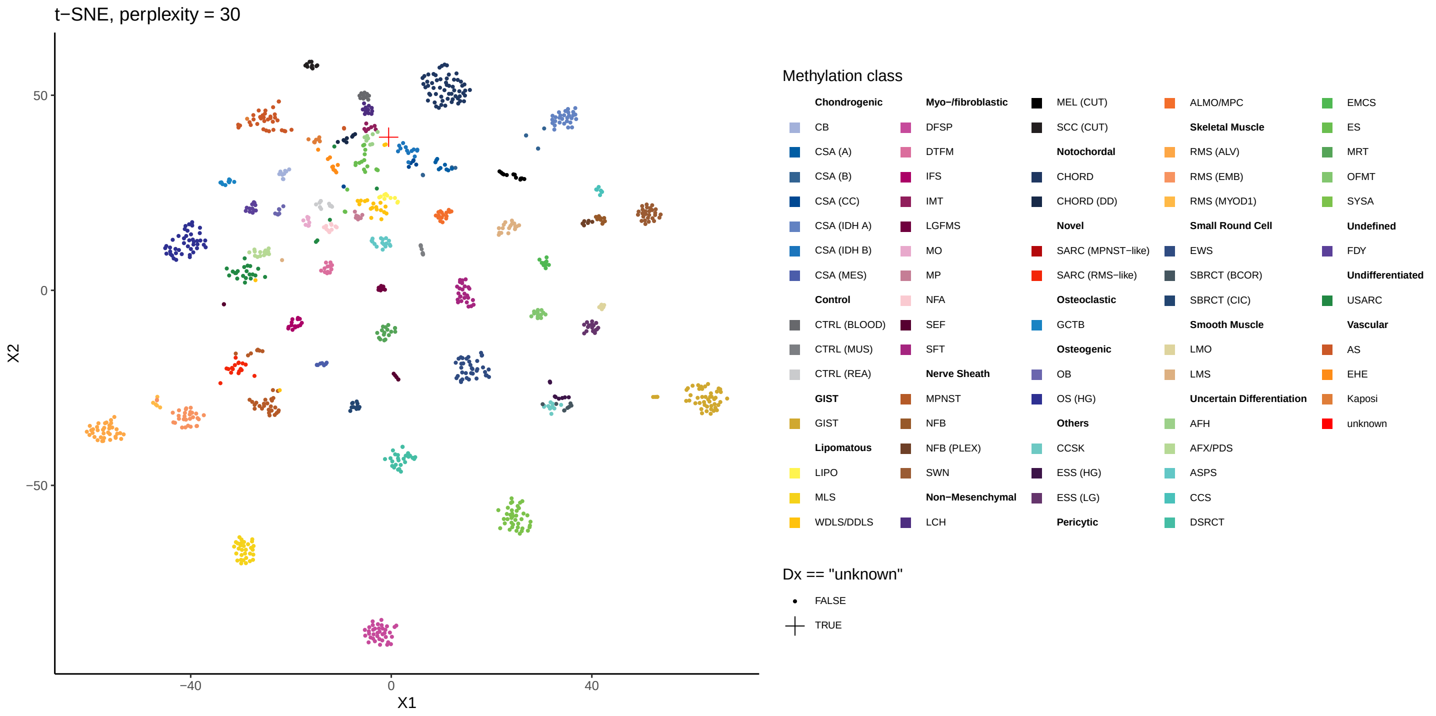

PATHOLOGY MICROSCOPIC

Well differentiated liposarcoma with lipoma like, sclerosing and inflammatory type components.
FNNLC: G1
Size:28cm in the retroperitoneum,
The tumor invades through the diaphragm
The radial margin is focally involved
The superior margin can't be accessed
Surgical margins of the colon and the small bowel unremarkable
Appendix unremarkable

Nanopore sequencing report

SARC-09

Quality control metrics

Barcode statistics

```
Adapters detected in 41804 of 42284 reads
         RBK004  41804: |  ################### |  98.86 %
           none    480: |                      |   1.14 %
Barcodes detected in 41804 of 42284 adapters
      barcode06  41804: |  ################### |  98.86 %
           none    480: |                      |   1.14 %
Demultiplexing finished in 26.26s
```

Read statistics

```
General summary:
Average percent identity:           92.3
Fraction of bases aligned:            1.0
Mean read length:                5,563.6
Mean read quality:                  12.0
Median percent identity:            93.4
Median read length:              1,715.0
Median read quality:                12.1
Number of reads:                39,969.0
Read length N50:                15,893.0
STDEV read length:               8,678.8
Total bases:               222,371,071.0
Total bases aligned:       214,167,739.0
Number, percentage and megabases of reads above quality cutoffs
>Q5:    39958 (100.0%) 222.4Mb
```

Mean genome coverage is 0.07X.

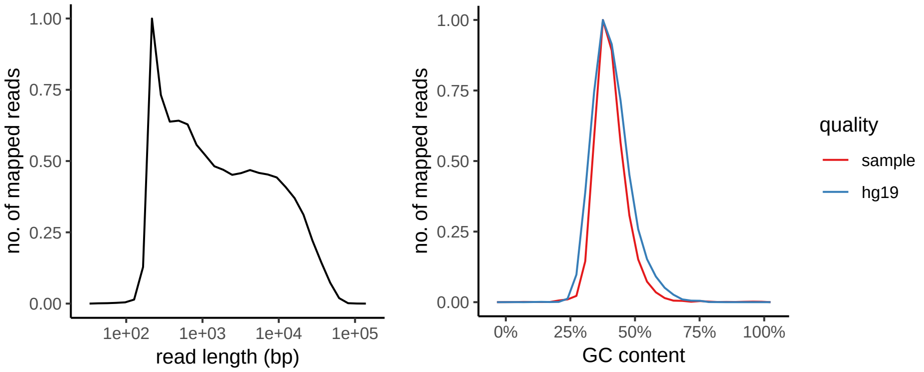

Copy number profile

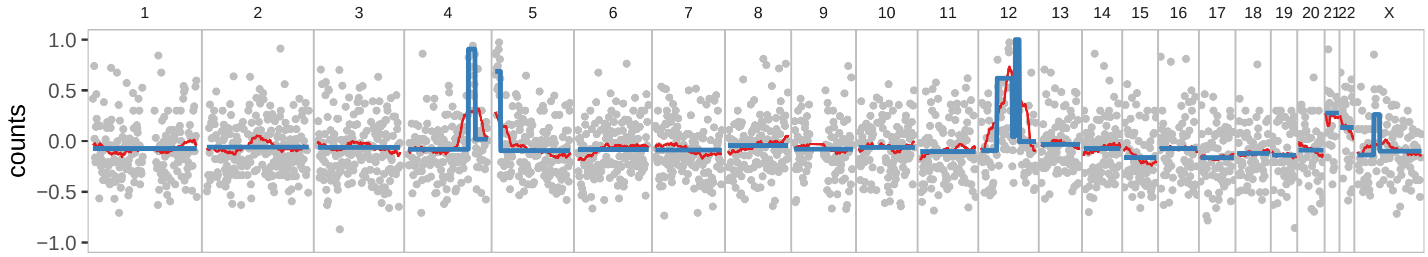

Methylation based classification

Methylation-based classification is based on **16427** CpG sites (overlap of sites covered in this sample and the training set). The out-of-bag error of this ad-hoc classifier is **2%**. Using this classifier, the sample has been classified as **well- / dedifferentiated liposarcoma**. This prediction has a confidence score of **0.45**. The raw and calibrated score distribution for the Top 10 entities is given below.

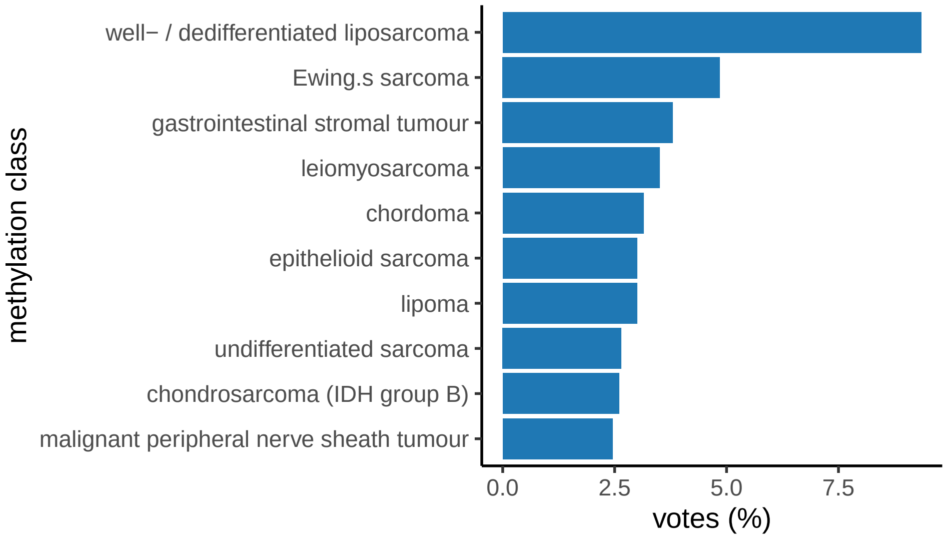

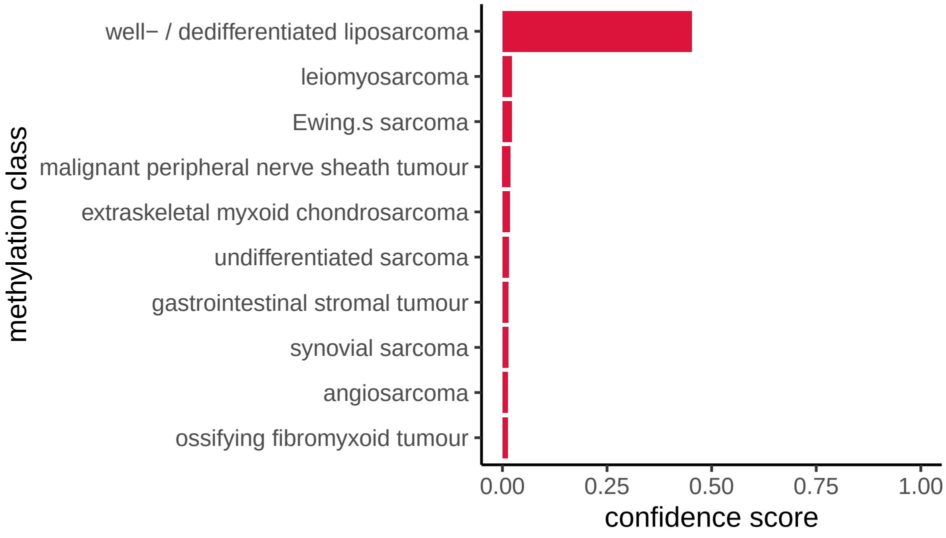

t-SNE plot

t-SNE plots are meant for visual quality control, not classification, and must be interpreted with care.

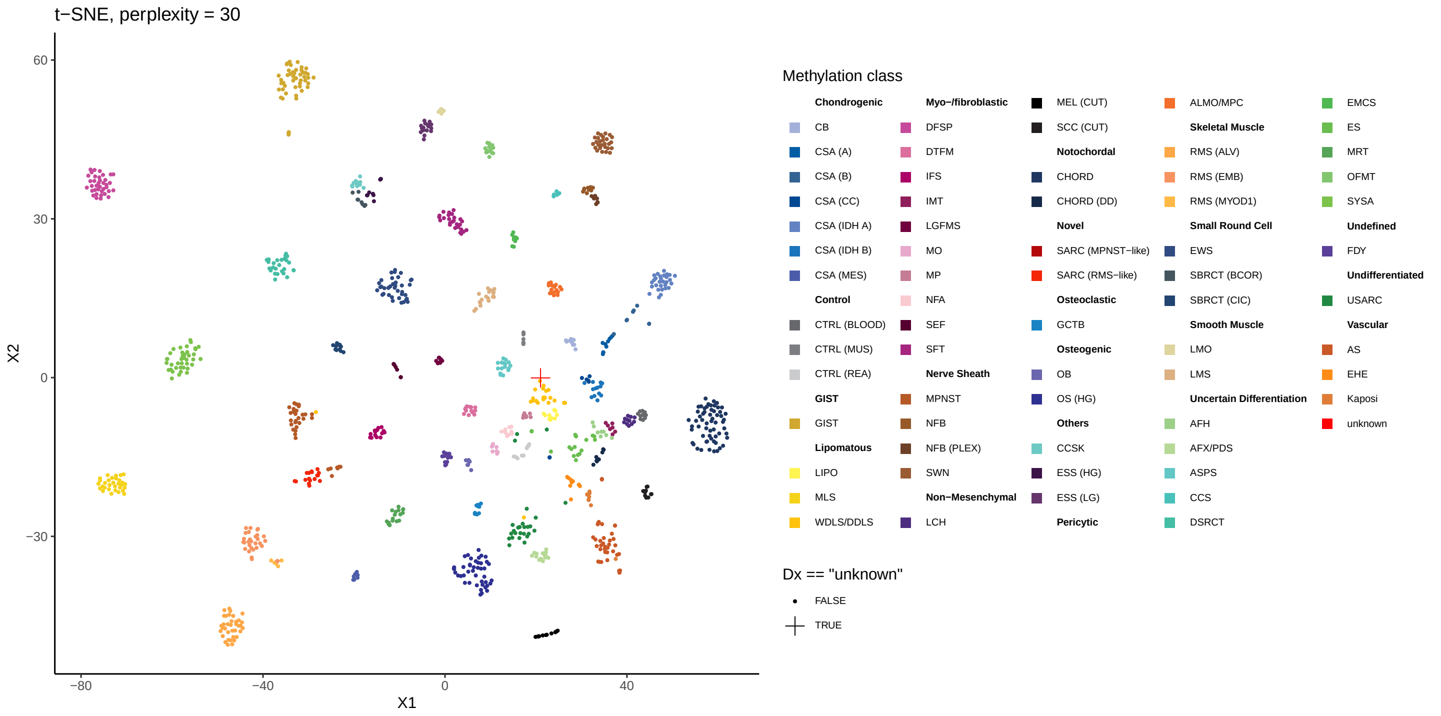

PATHOLOGY MICROSCOPIC

Soft tissue; lt retroperitoneum; resection:
Myxoid low grade tumor composed of small ovoidal cells with scant cytoplasm and minimal to no atypia, embedded in a highly myxoid stroma with numerous thin walled blood vessels and numerous small ropery collagen bundles. Small clusters of lymphocytes are present. Few small foci of fat tissue with lipoblasts, atypical nuclei and myxoid changes are present at the periphery of the lesion.
The lesion is mostly well circumscribed, with clear margins from the surrounding tissue and structures.
Necrosis is present, comprising less than 50% of the mass.
Isolated mitoses identified.
Margins of excision - the lateral and superior margins are involved. The remaining margins are free. Distance to margins:
anterior - 1 cm
posterior - 0.3 cm
inferior - 0.3 cm
medial - 0.2 cm
The spleen, pancreas and adrenal are not involved by the tumor.
Immunostains:
MDM2, CDK4, CD34, S100 - positive
H3K27me3 - almost complete loss, with few positive nuclei.
GFAP; SOX10; neurofilaments - negative.

Discussion:
This is a low grade myxoid sarcoma with small areas suggestive of well differentiated liposarcoma and extensive areas with neuronal appearance.

Conclusion:
Highly suspicious for well differentiated liposarcoma with extensive myxoid changes.
Confirmatory molecular assay such as FISH for MDM2 gene rearrangement is recommended.

Nanopore sequencing report

SARC-10

Quality control metrics

Barcode statistics

```
Adapters detected in 67417 of 68047 reads
         RBK004  67417: |  ################### |  99.07 %
           none    628: |                      |   0.92 %
Barcodes detected in 67417 of 68047 adapters
      barcode07  67417: |  ################### |  99.07 %
           none    628: |                      |   0.92 %
2 reads were skipped due to the min. length filter.
Demultiplexing finished in 41.38s
```

Read statistics

```
General summary:
Average percent identity:           92.6
Fraction of bases aligned:           1.0
Mean read length:                3,080.8
Mean read quality:                  12.1
Median percent identity:            93.6
Median read length:              1,295.0
Median read quality:                12.2
Number of reads:                66,443.0
Read length N50:                 7,188.0
STDEV read length:               4,339.5
Total bases:               204,696,320.0
Total bases aligned:       194,779,012.0
Number, percentage and megabases of reads above quality cutoffs
>Q5:    66439 (100.0%) 204.7Mb
```

Mean genome coverage is 0.06X.

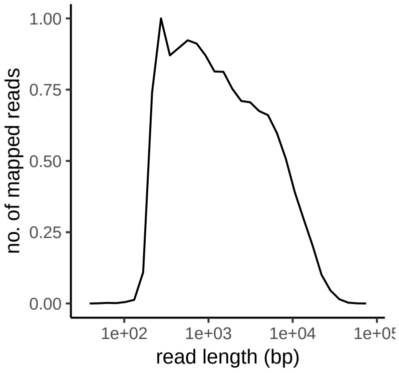

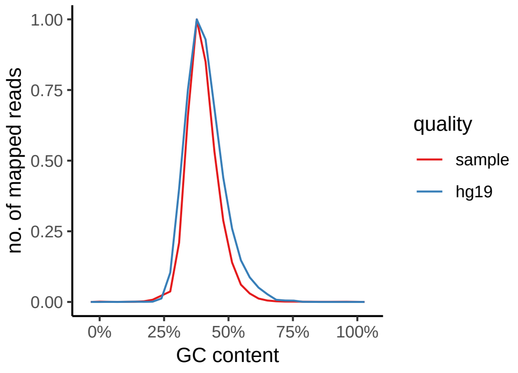

Copy number profile

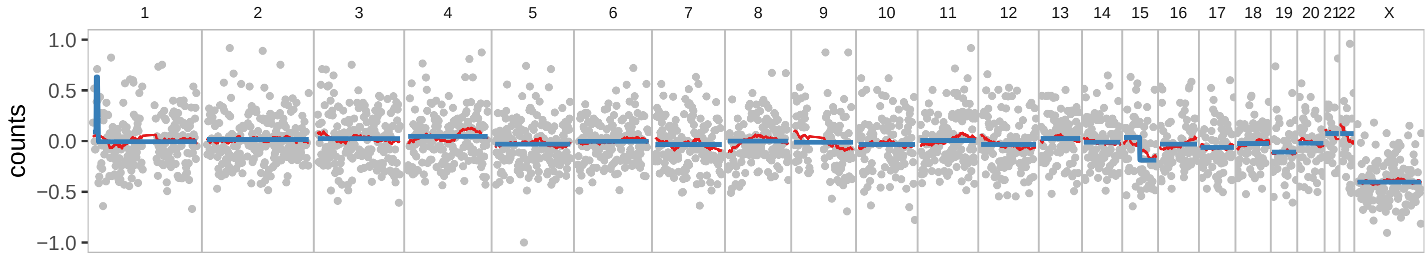

Methylation based classification

Methylation-based classification is based on **14329** CpG sites (overlap of sites covered in this sample and the training set). The out-of-bag error of this ad-hoc classifier is **2.9%**. Using this classifier, the sample has been classified as **Ewing´s sarcoma**. This prediction has a confidence score of **0.14**. The raw and calibrated score distribution for the Top 10 entities is given below.

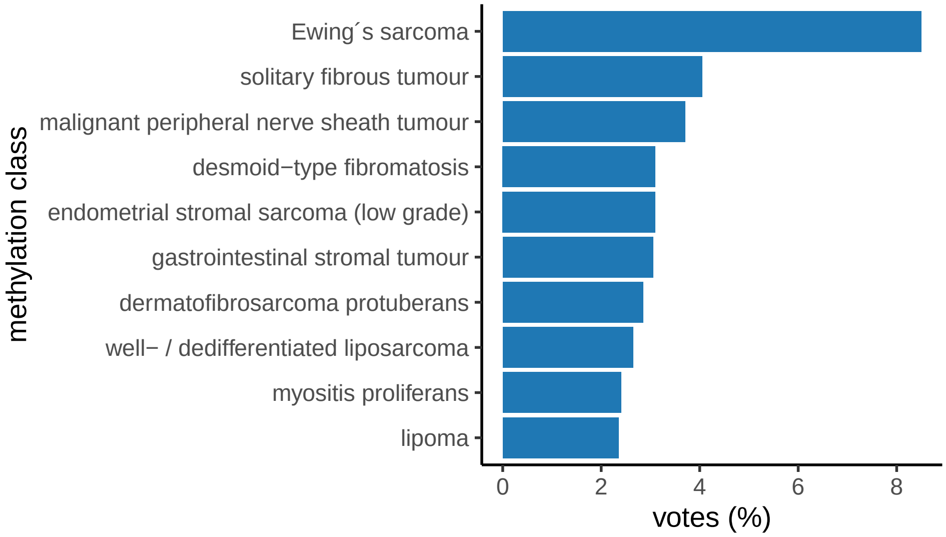

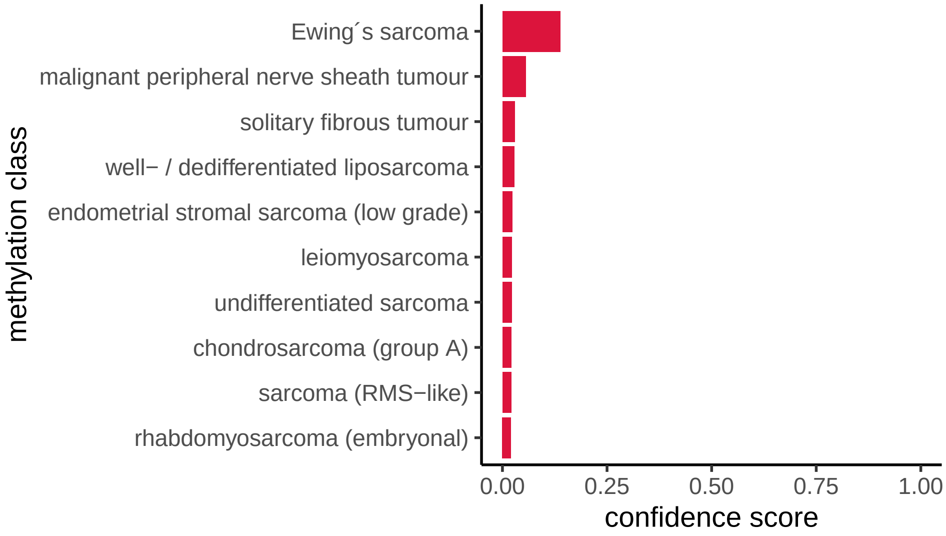

t-SNE plot

t-SNE plots are meant for visual quality control, not classification, and must be interpreted with care.

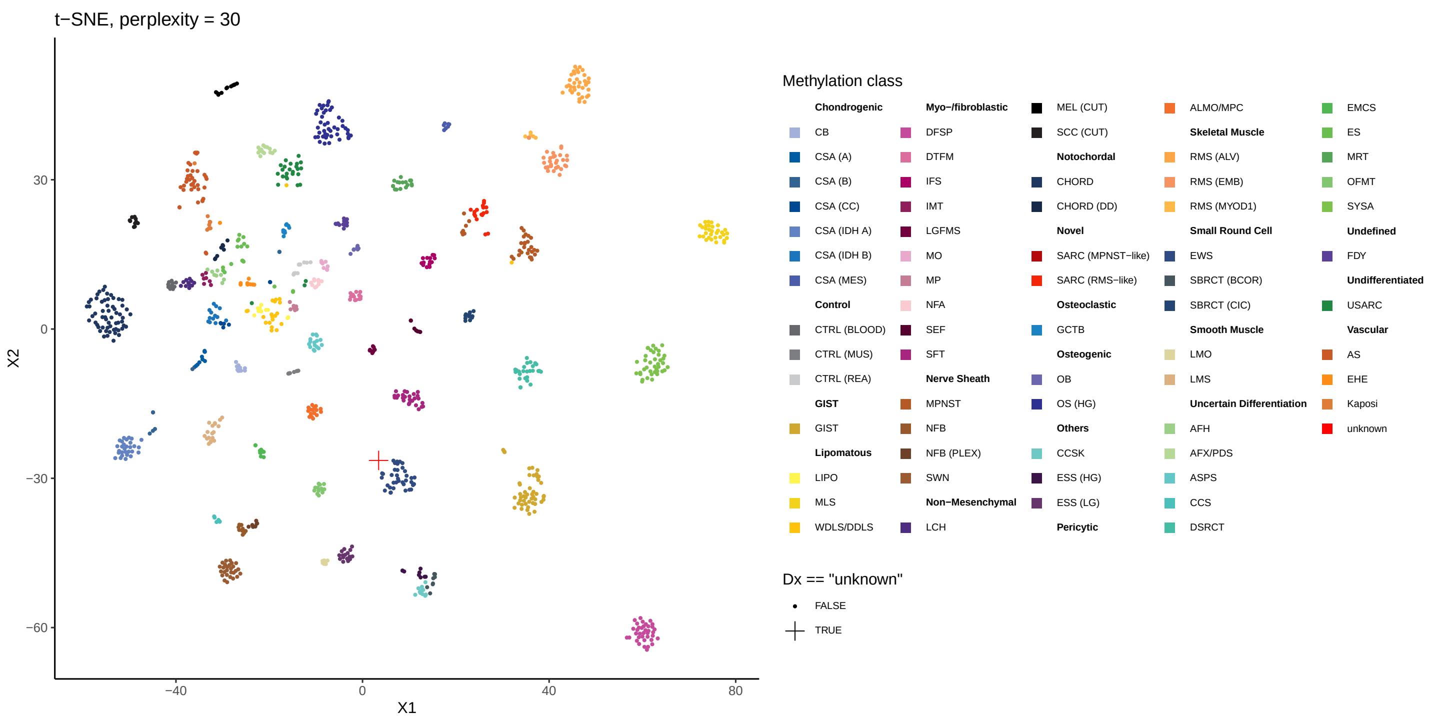

<u>**PATHOLOGY MICROSCOPIC**</u>

Soft tissue mass, shoulder, excision:

- Small round blue cell tumor highly suggestive of Ewing sarcoma (see biopsy number 11380/19 for immunophenotyping results)

- Most of the tumor is viable (a whole slice was examined)

- Surgical margins are free

Nanopore sequencing report

SARC-11

Quality control metrics

Barcode statistics

```
Adapters detected in 21395 of 21952 reads
         RBK004  21395: |  ################### |  97.46 %
           none    557: |                      |   2.54 %
Barcodes detected in 21395 of 21952 adapters
      barcode08  21395: |  ################### |  97.46 %
           none    557: |                      |   2.54 %
Demultiplexing finished in 14.90s
```

Read statistics

```
General summary:
Average percent identity:          90.7
Fraction of bases aligned:           1.0
Mean read length:               5,243.7
Mean read quality:                 11.3
Median percent identity:           91.7
Median read length:             2,330.0
Median read quality:               11.3
Number of reads:               21,717.0
Read length N50:               12,176.0
STDEV read length:              6,982.2
Total bases:              113,876,533.0
Total bases aligned:      109,971,296.0
Number, percentage and megabases of reads above quality cutoffs
>Q5:    21709 (100.0%) 113.9Mb
```

Mean genome coverage is 0.04X.

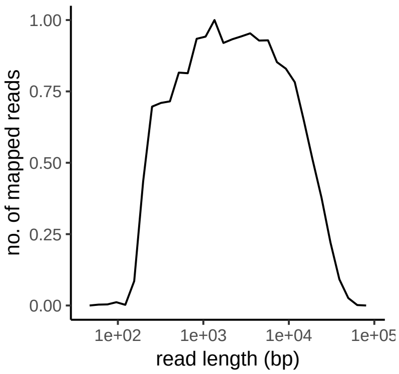

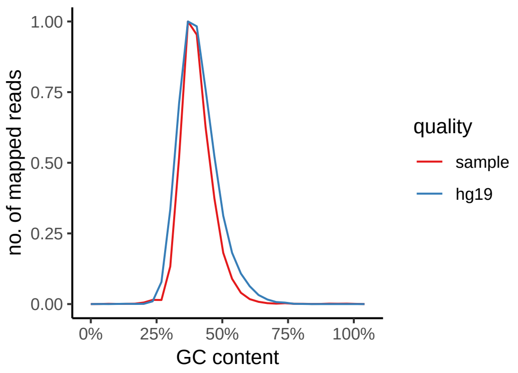

Copy number profile

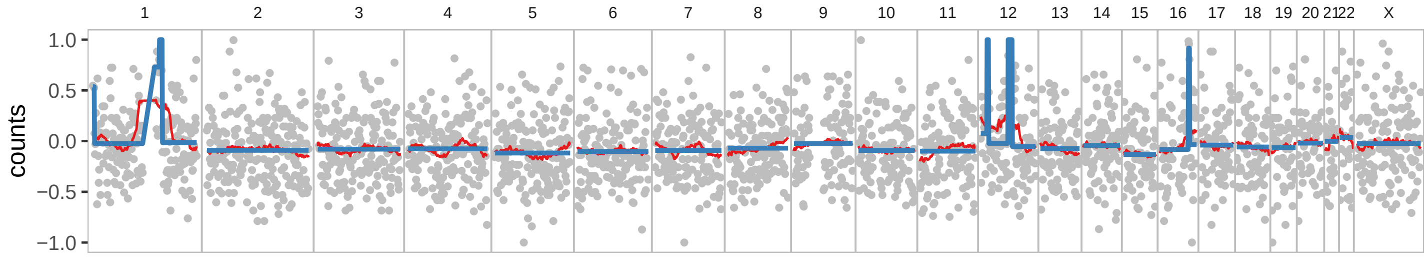

Methylation based classification

Methylation-based classification is based on **7545** CpG sites (overlap of sites covered in this sample and the training set). The out-of-bag error of this ad-hoc classifier is **2.6%**. Using this classifier, the sample has been classified as **well- / dedifferentiated liposarcoma**. This prediction has a confidence score of **0.15**. The raw and calibrated score distribution for the Top 10 entities is given below.

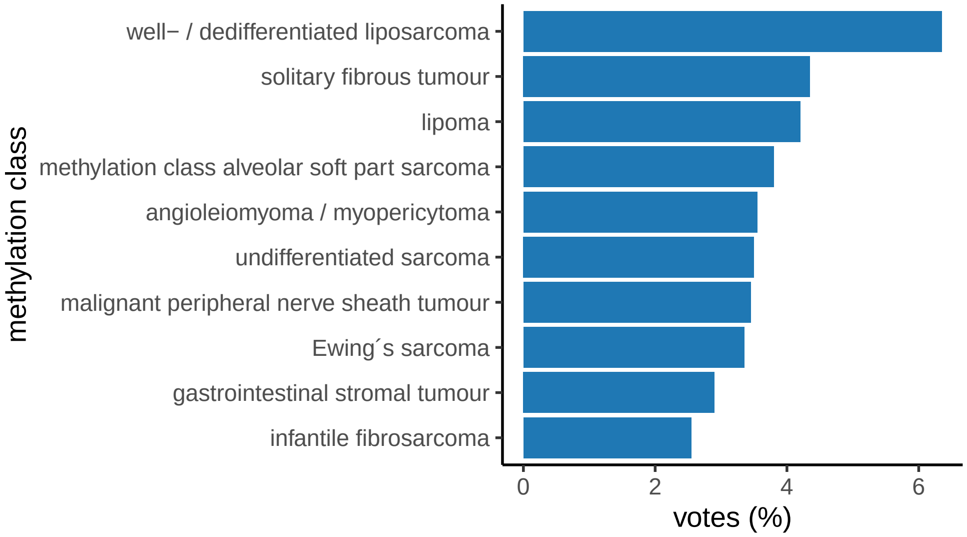

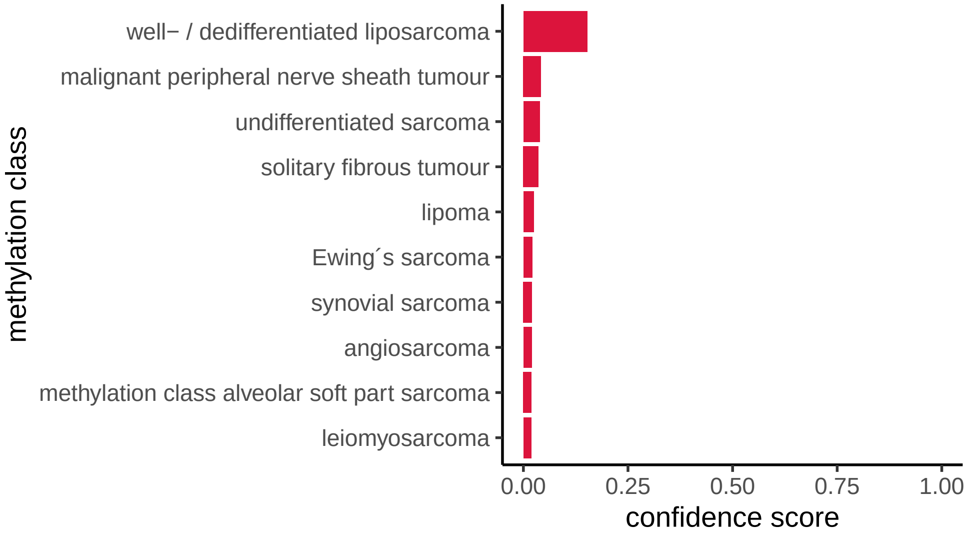

t-SNE plot

t-SNE plots are meant for visual quality control, not classification, and must be interpreted with care.

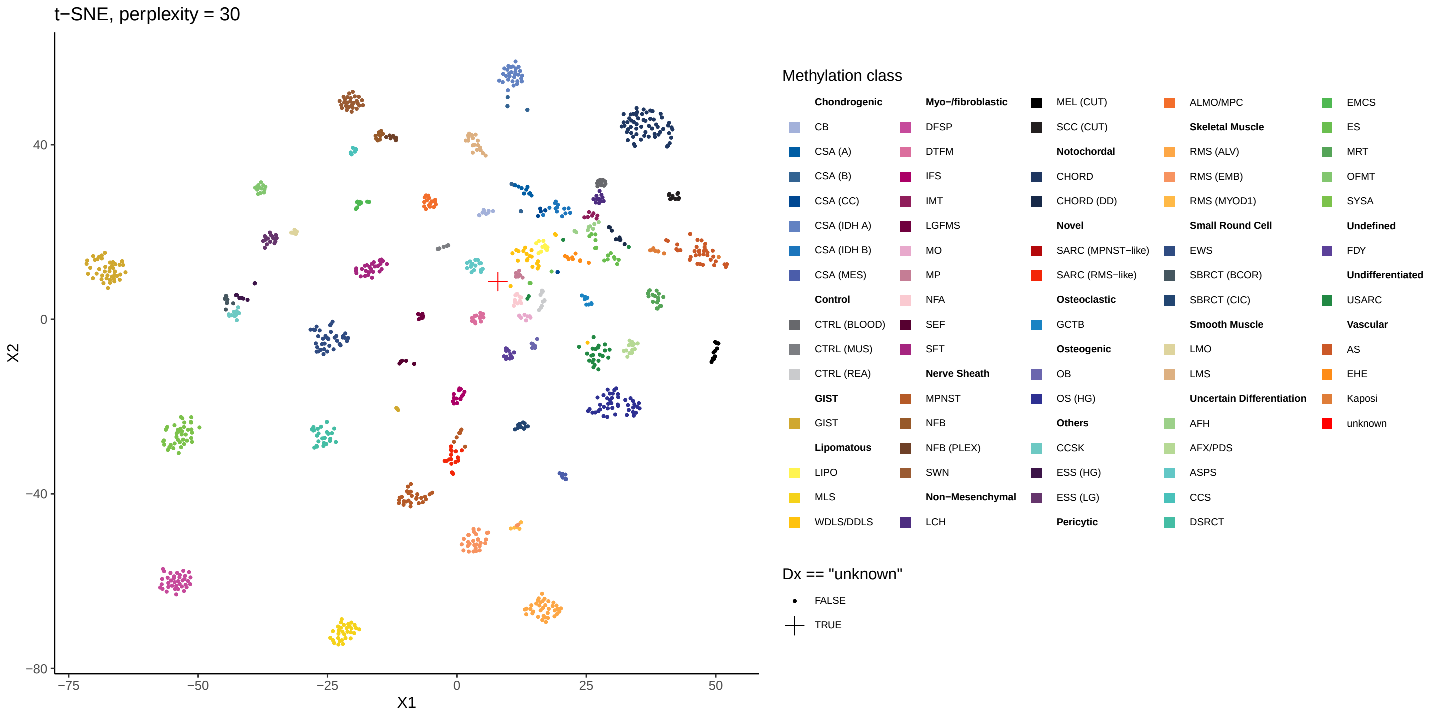

PATHOLOGY MICROSCOPIC

Well differentiated liposarcoma, lipoma-like. There is invasion to the striated muscle.
Surgical margins free of malignancy.
Closest margins:
Lateral margins: 5mm
Superior margin: 0.5mm (block 1-2)

Inferior margin: 2.2 mm
Posterior margin: 2.2 mm

Nanopore sequencing report

SARC-12

Quality control metrics

Barcode statistics

```
Adapters detected in 62293 of 63153 reads
         RBK004  62293: |  ################### |  98.64 %
           none    859: |                      |   1.36 %
Barcodes detected in 62293 of 63153 adapters
      barcode09  62293: |  ################### |  98.64 %
           none    859: |                      |   1.36 %
1 reads were skipped due to the min. length filter.
Demultiplexing finished in 35.89s
```

Read statistics

```
General summary:
Average percent identity:           92.4
Fraction of bases aligned:            0.9
Mean read length:                3,071.7
Mean read quality:                  11.9
Median percent identity:            93.3
Median read length:              1,069.0
Median read quality:                12.0
Number of reads:                61,029.0
Read length N50:                 8,849.0
STDEV read length:               5,117.5
Total bases:               187,463,516.0
Total bases aligned:       177,791,873.0
Number, percentage and megabases of reads above quality cutoffs
>Q5:    61007 (100.0%) 187.5Mb
```

Mean genome coverage is 0.06X.

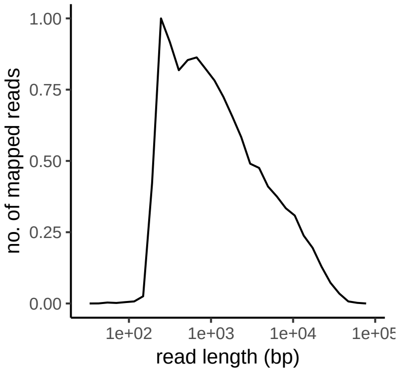

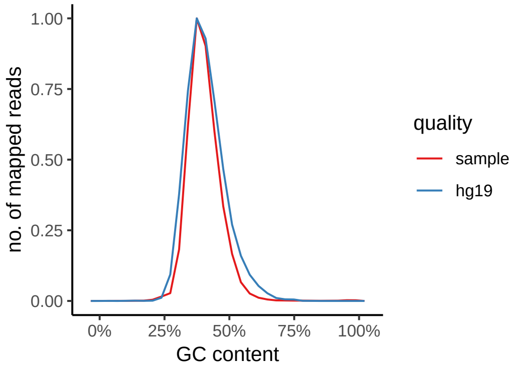

Copy number profile

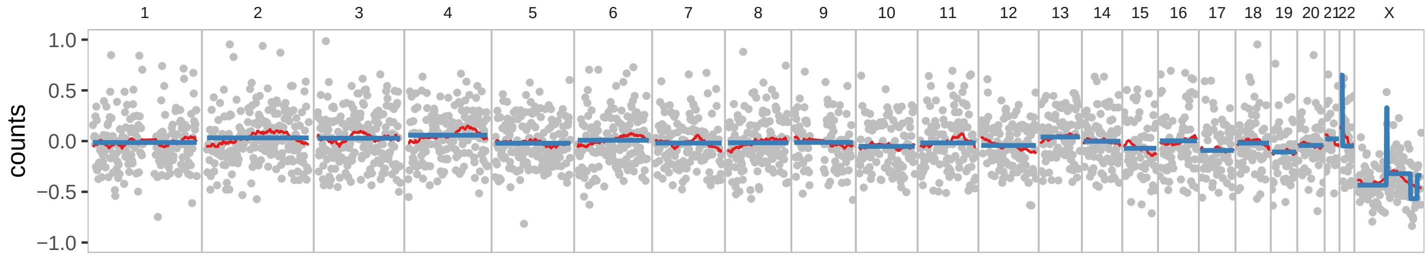

Methylation based classification

Methylation-based classification is based on **11745** CpG sites (overlap of sites covered in this sample and the training set). The out-of-bag error of this ad-hoc classifier is **2.1%**. Using this classifier, the sample has been classified as **well- / dedifferentiated liposarcoma**. This prediction has a confidence score of **0.07**. The raw and calibrated score distribution for the Top 10 entities is given below.

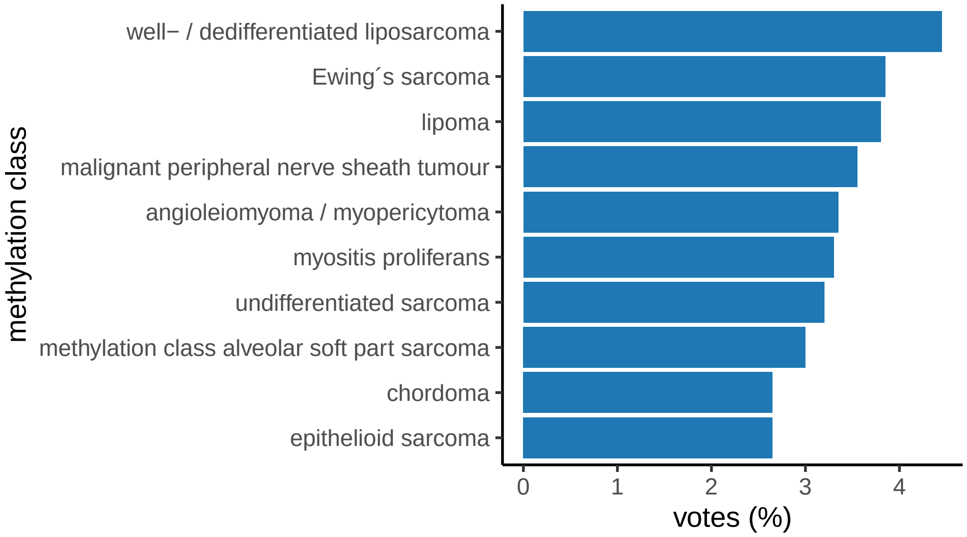

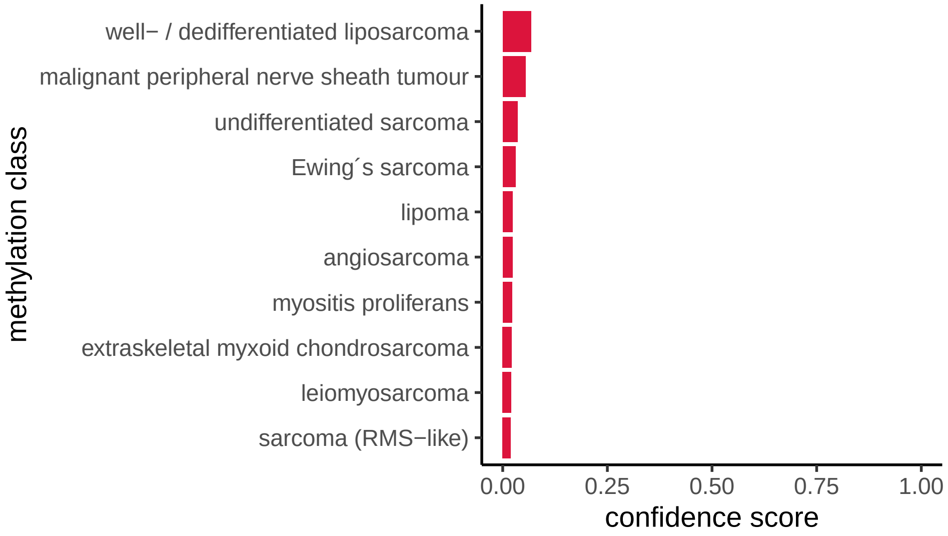

t-SNE plot

t-SNE plots are meant for visual quality control, not classification, and must be interpreted with care.

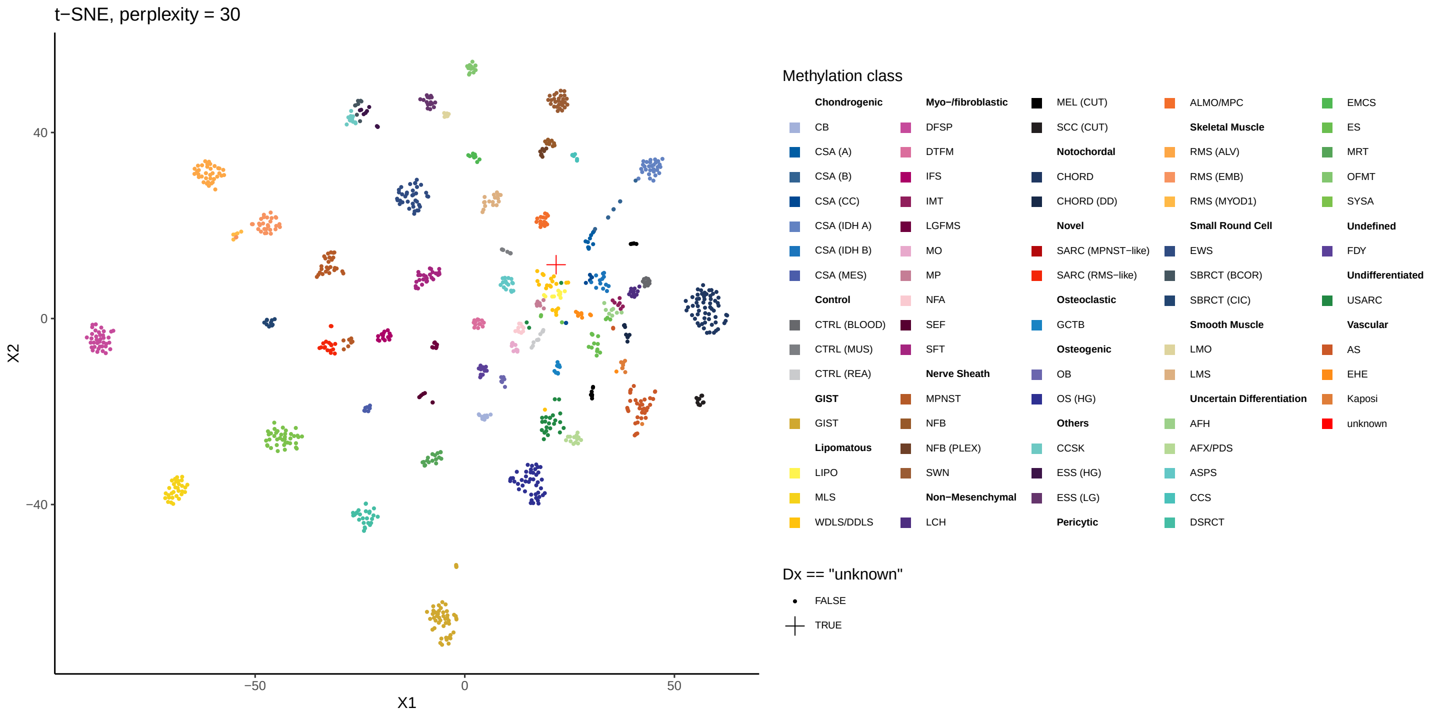

PATHOLOGY MICROSCOPIC

Soft tissue, rt. medial thigh, mass, wide excision:

Myxofibrosarcoma, low grade 7.7 cm in diameter.
Free resection margins.
Tumor is 0.6 cm from the inked posterior margin and 0.8 cm from the medial margin. The other margins are at least 2 cm away.
The fragment of femoral vein is free of tumor.
Treatment effects:
- No necrosis identified.
- Very scant sclerosis with focal liquefaction of the myxoid stroma
- The viable tumor cells comprises at least 40% of the measured lesion.

Nanopore sequencing report

SARC-13

Quality control metrics

Barcode statistics

```
Adapters detected in 108896 of 110789 reads
         RBK004 108896: |  ################### |  98.29 %
           none   1893: |                      |   1.71 %
Barcodes detected in 108896 of 110789 adapters
      barcode10 108896: |  ################### |  98.29 %
           none   1893: |                      |   1.71 %
Demultiplexing finished in 64.15s
```

Read statistics

```
General summary:
Average percent identity:          92.7
Fraction of bases aligned:           0.9
Mean read length:               1,774.0
Mean read quality:                 11.8
Median percent identity:           93.6
Median read length:               851.0
Median read quality:               11.9
Number of reads:              106,661.0
Read length N50:                3,621.0
STDEV read length:              2,488.8
Total bases:              189,214,283.0
Total bases aligned:      175,610,257.0
Number, percentage and megabases of reads above quality cutoffs
>Q5:    106656 (100.0%) 189.2Mb
```

Mean genome coverage is 0.06X.

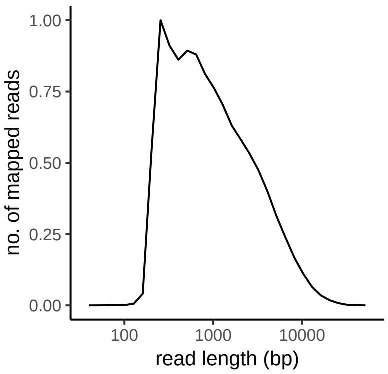

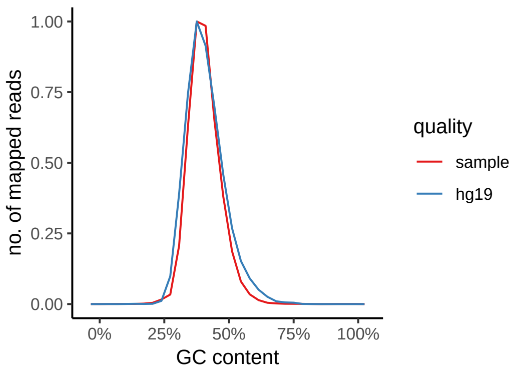

Copy number profile

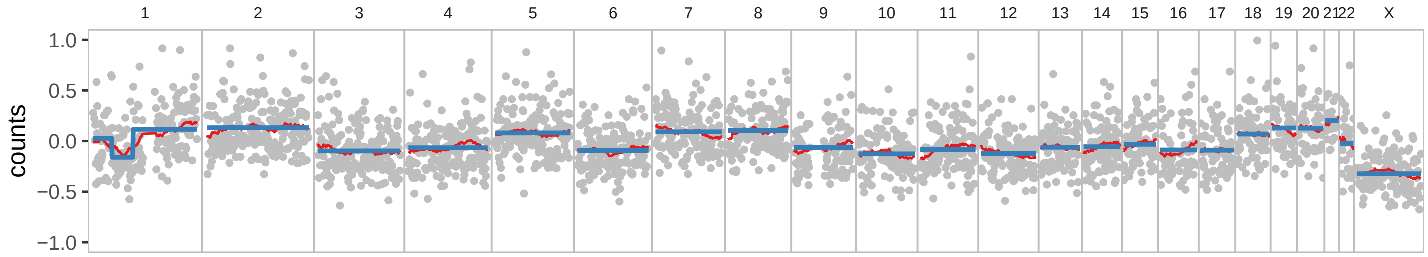

Methylation based classification

Methylation-based classification is based on **13983** CpG sites (overlap of sites covered in this sample and the training set). The out-of-bag error of this ad-hoc classifier is **2.8%**. Using this classifier, the sample has been classified as **malignant peripheral nerve sheath tumour**. This prediction has a confidence score of **0.04**. The raw and calibrated score distribution for the Top 10 entities is given below.

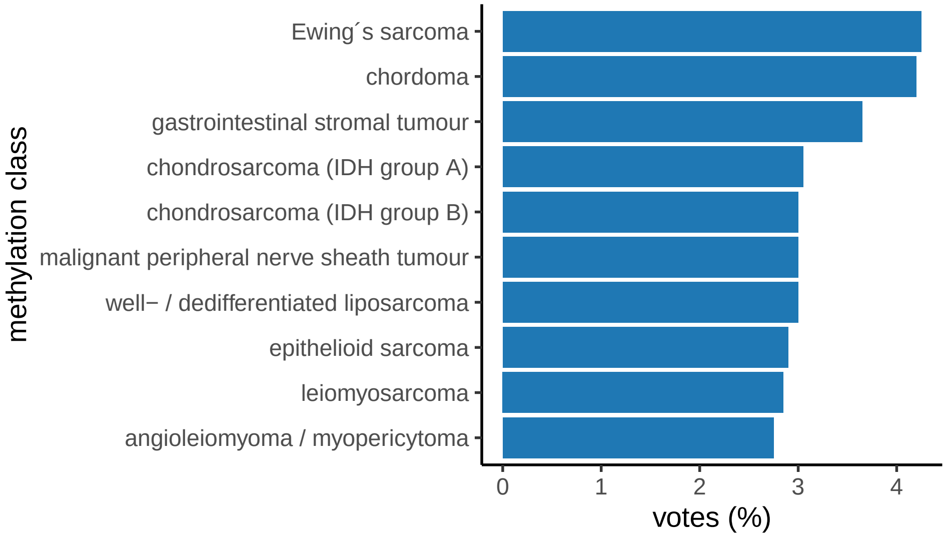

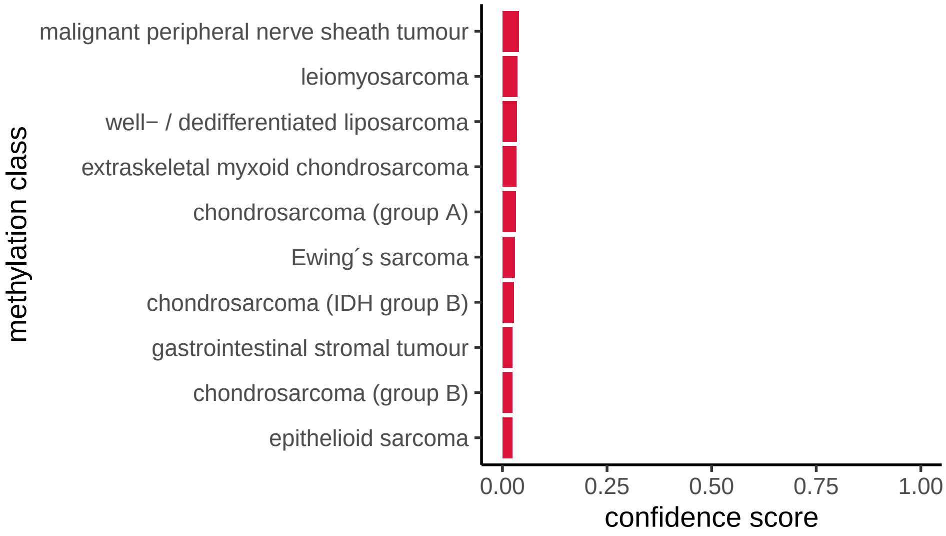

t-SNE plot

t-SNE plots are meant for visual quality control, not classification, and must be interpreted with care.

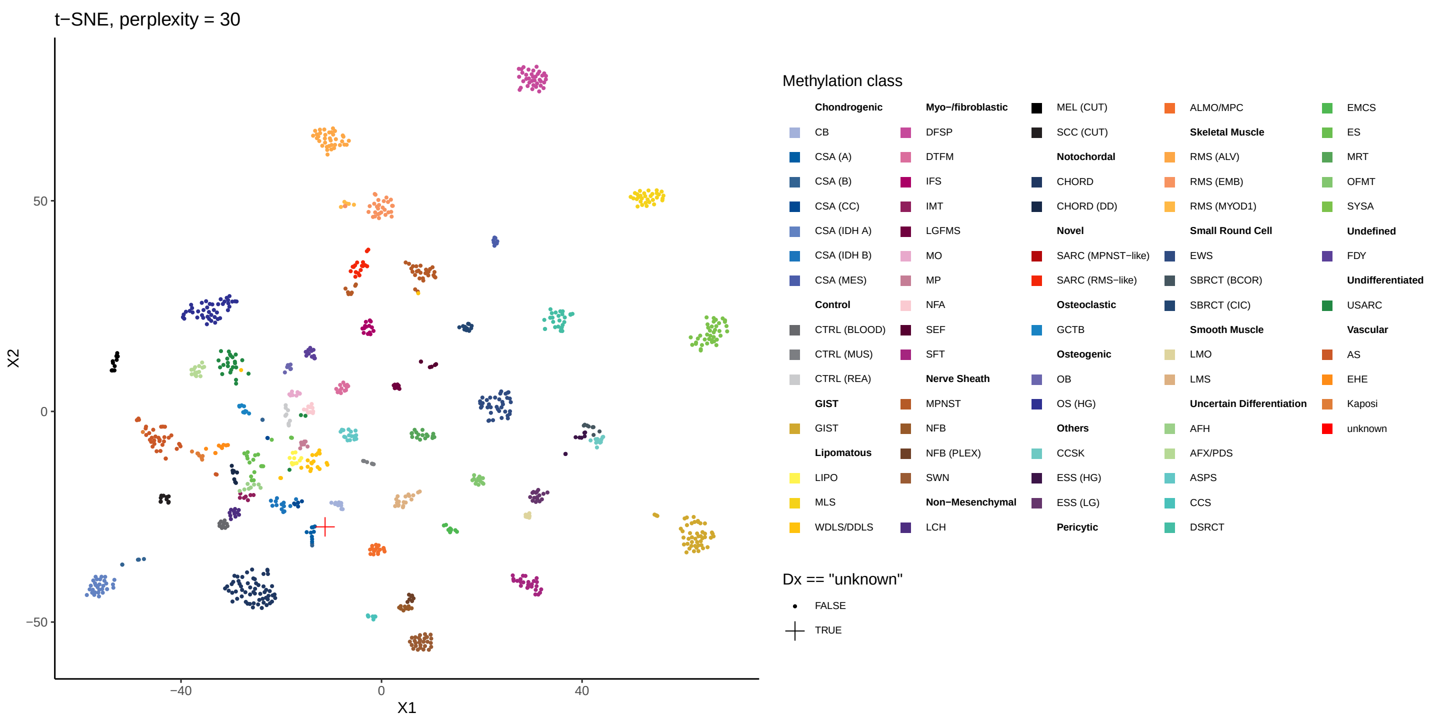

PATHOLOGY MICROSCOPIC

Bone and soft tissue; Rt. 8-10 ribs resection:

High grade chondrosarcoma (grade 2-3) with clear cell features.

Size 6.5 cm.
The surgical margins through the bone and soft tissue are free.
One focus suspicious for perineural invasion.
No vascular invasion identified.

Note: There is upgrading of the tumor in comparison with the previous resection (201712050).
Although there is a large component of the tumor showing clear cell features, with trabeculae of bone and lobules of well differentiated chondroid tumor, there are foci showing sheets of cells with high cellularity, loss of lacunae, myxoid change, spindle cells, 2 mitoses/10 HPF and nuclear atypia.

Nanopore sequencing report

SARC-15

Quality control metrics

Barcode statistics

```
Adapters detected in 101949 of 106827 reads
         RBK004 101949: |  ################### |  95.43 %
           none   4874: |                      |   4.56 %
Barcodes detected in 101949 of 106827 adapters
      barcode09      1: |                      |   0.00 %
      barcode12 101948: |  ################### |  95.43 %
           none   4874: |                      |   4.56 %
4 reads were skipped due to the min. length filter.
Demultiplexing finished in 64.13s
```

Read statistics

```
General summary:
Average percent identity:           92.6
Fraction of bases aligned:            0.9
Mean read length:                1,262.0
Mean read quality:                  11.5
Median percent identity:            93.4
Median read length:                641.0
Median read quality:                11.5
Number of reads:                97,419.0
Read length N50:                 2,397.0
STDEV read length:               1,559.6
Total bases:               122,940,793.0
Total bases aligned:       111,184,823.0
Number, percentage and megabases of reads above quality cutoffs
>Q5:    97414 (100.0%) 122.9Mb
```

Mean genome coverage is 0.04X.

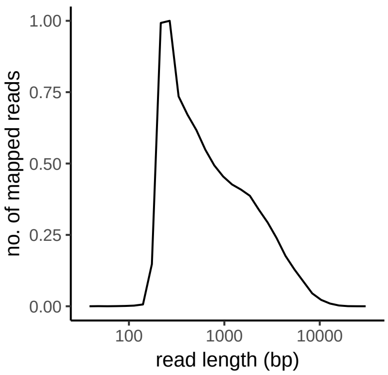

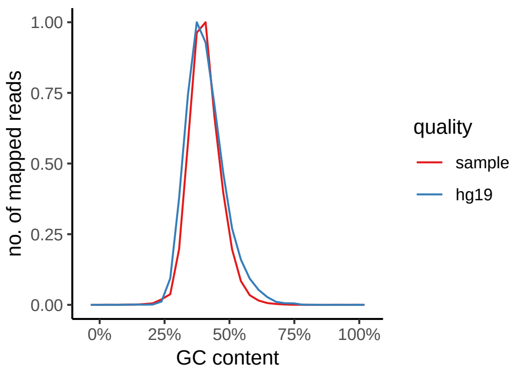

Copy number profile

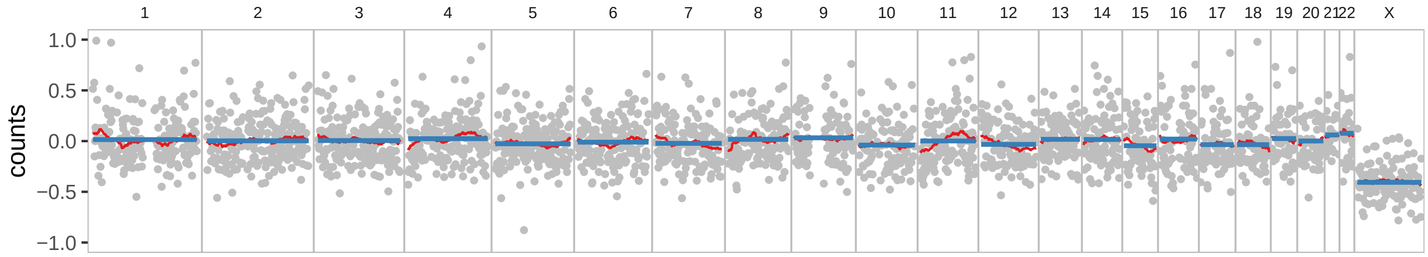

Methylation based classification

Methylation-based classification is based on **7436** CpG sites (overlap of sites covered in this sample and the training set). The out-of-bag error of this ad-hoc classifier is **3.2%**. Using this classifier, the sample has been classified as **undifferentiated sarcoma**. This prediction has a confidence score of **0.03**. The raw and calibrated score distribution for the Top 10 entities is given below.

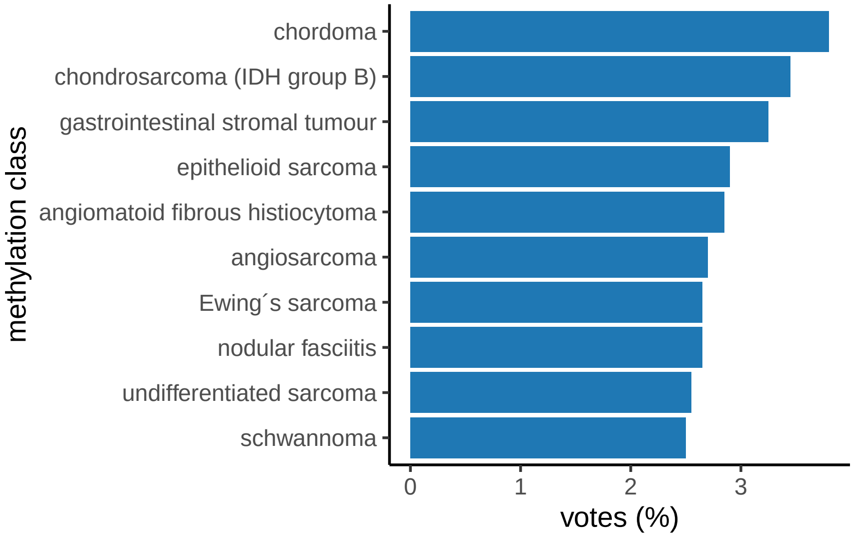

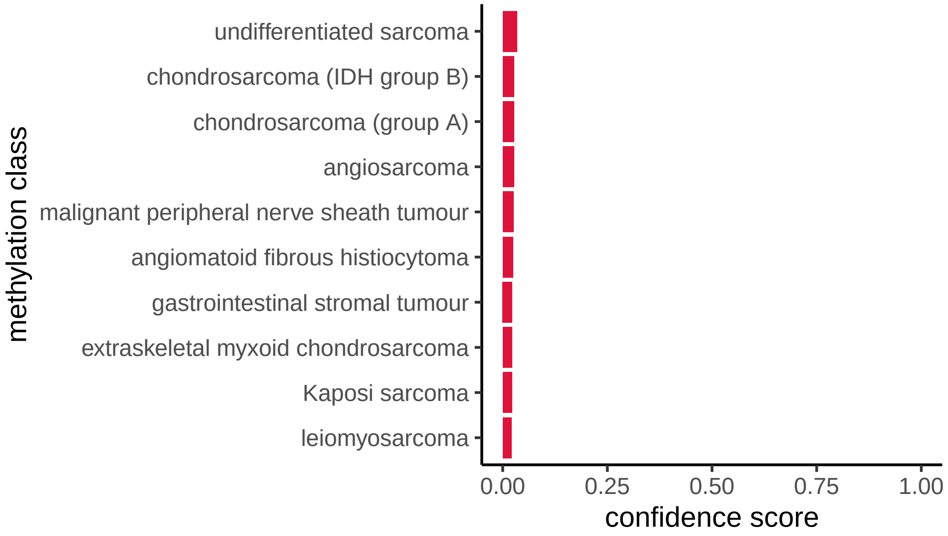

t-SNE plot

t-SNE plots are meant for visual quality control, not classification, and must be interpreted with care.

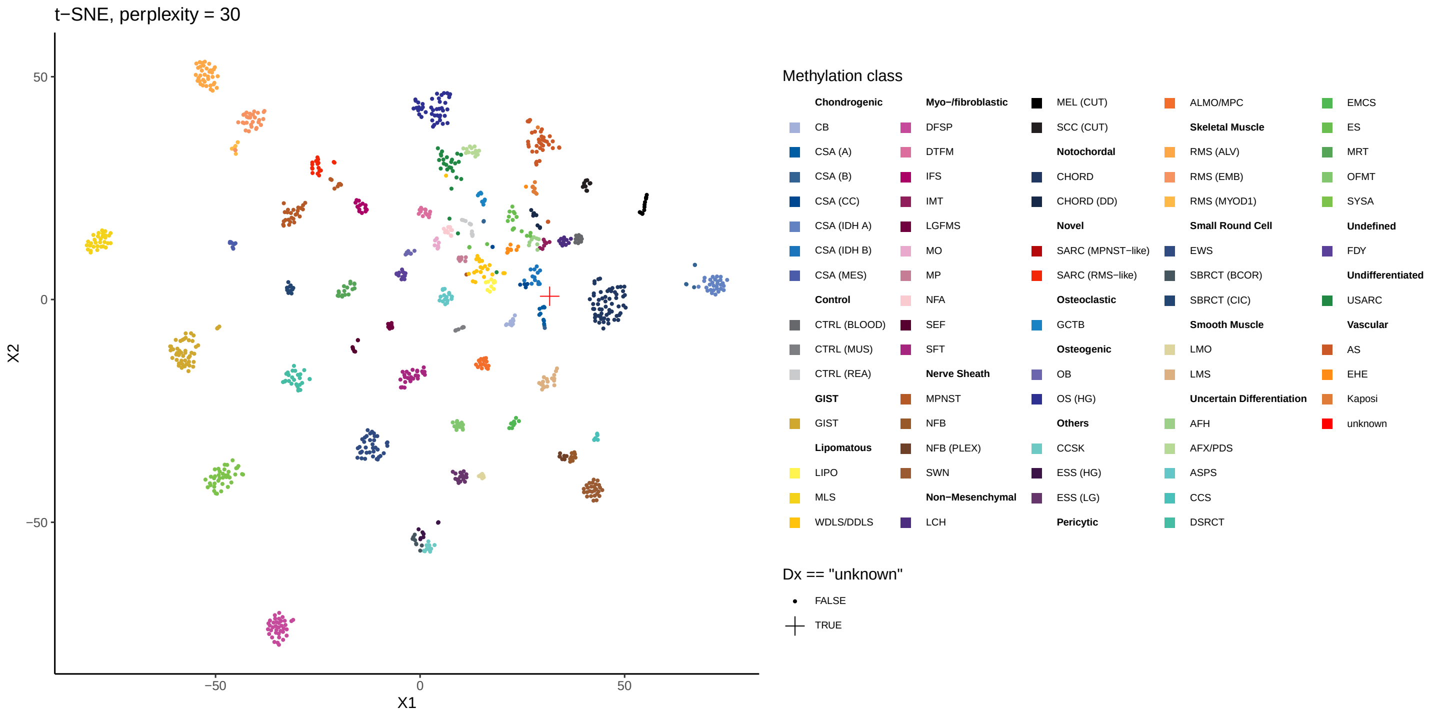

PATHOLOGY MICROSCOPIC

Mass, left thigh, post-treatment resection:
Negative for residual tumor.
In the tumoral bed there is paucicellular tissue with hyalinization, extensive necrosis, foamy macrophages with organizing tissue, chronic inflammation and entrapped skeletal muscle fibers. No viable tumor tissue identified.
The surgical margins are free.

Nanopore sequencing report

SARC-17

Quality control metrics

Barcode statistics

```
Adapters detected in 29983 of 30191 reads
         RBK004  29983: |  ################### |  99.31 %
           none    208: |                      |   0.69 %
Barcodes detected in 29983 of 30191 adapters
      barcode02  29983: |  ################### |  99.31 %
           none    208: |                      |   0.69 %
Demultiplexing finished in 28.18s
```

Read statistics

```
General summary:
Average percent identity:            92.7
Fraction of bases aligned:             1.0
Mean read length:                 5,899.5
Mean read quality:                   12.4
Median percent identity:             93.9
Median read length:               2,355.5
Median read quality:                 12.5
Number of reads:                 29,826.0
Read length N50:                 14,491.0
STDEV read length:                8,297.8
Total bases:                175,959,152.0
Total bases aligned:        169,877,172.0
Number, percentage and megabases of reads above quality cutoffs
>Q5:    29821 (100.0%) 176.0Mb
```

Mean genome coverage is 0.06X.

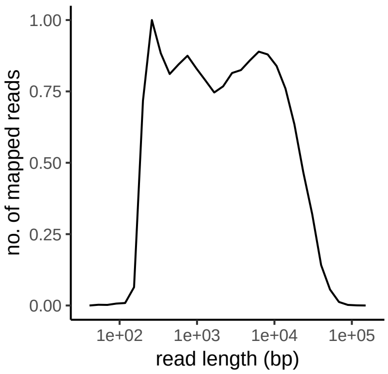

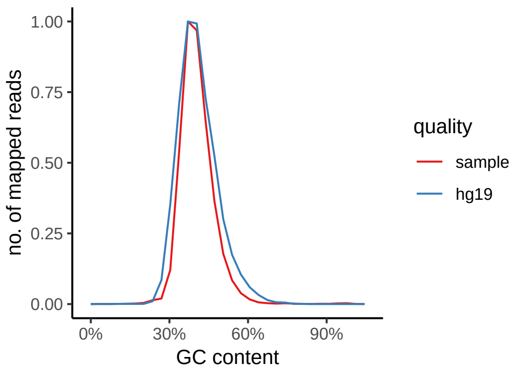

Copy number profile

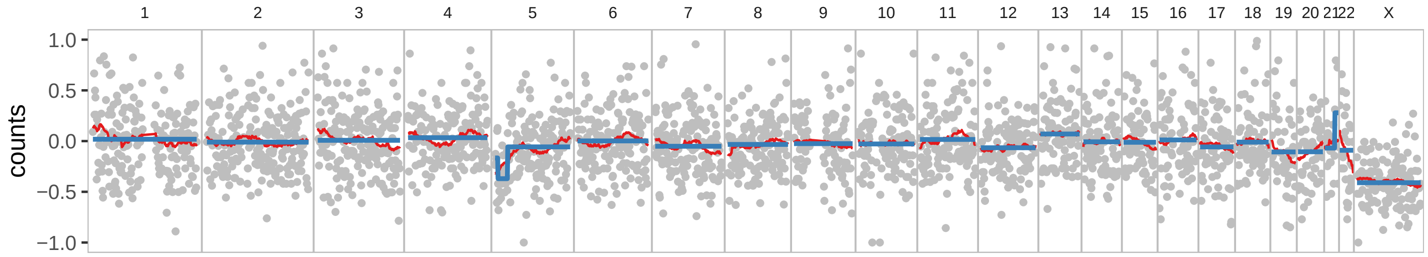

Methylation based classification

Methylation-based classification is based on **13342** CpG sites (overlap of sites covered in this sample and the training set). The out-of-bag error of this ad-hoc classifier is **2.4%**. Using this classifier, the sample has been classified as **myxoid liposarcoma**. This prediction has a confidence score of **0.36**. The raw and calibrated score distribution for the Top 10 entities is given below.

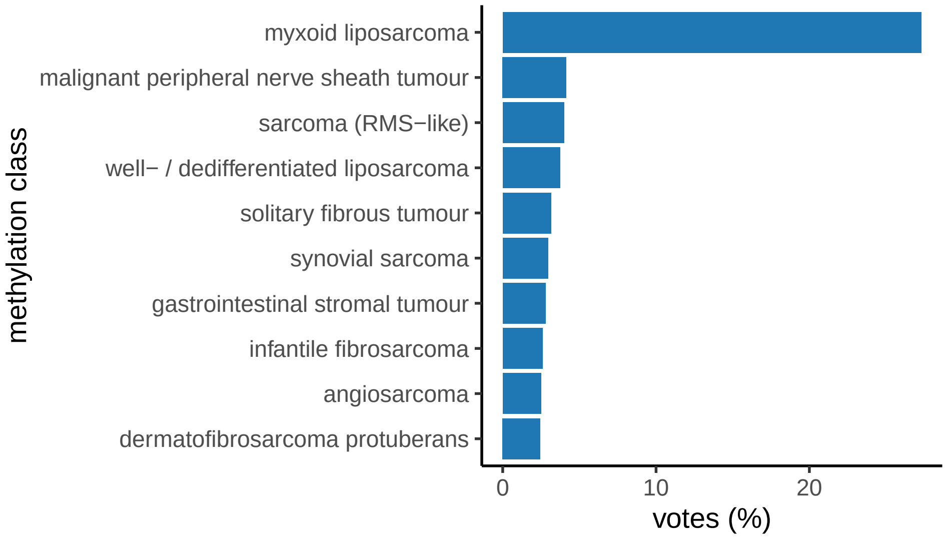

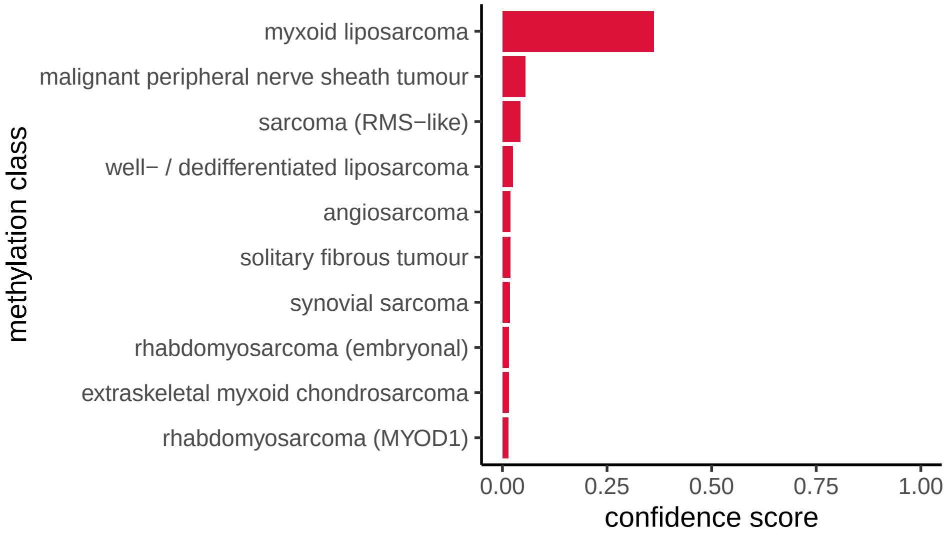

t-SNE plot

t-SNE plots are meant for visual quality control, not classification, and must be interpreted with care.

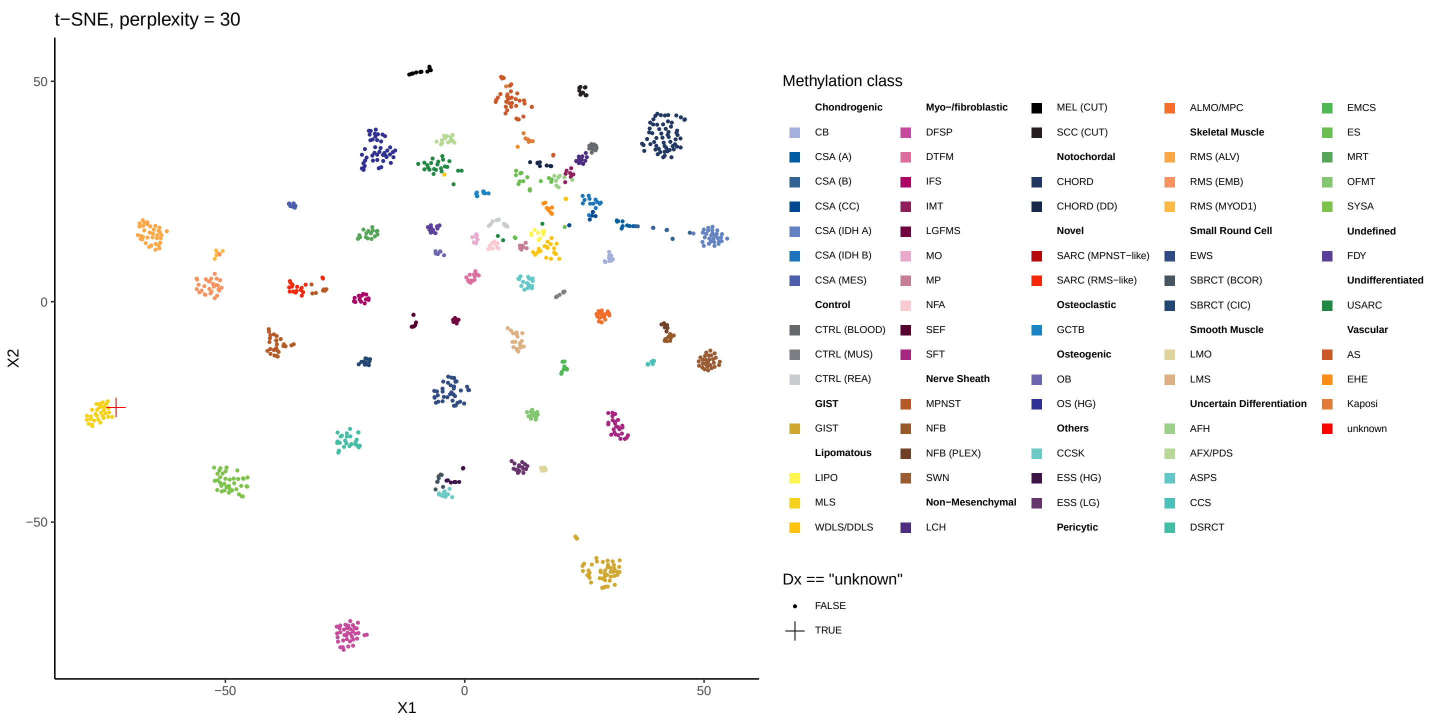

PATHOLOGY MICROSCOPIC

Soft tissue, Lt proximal thigh; tumor resection:
Myxoid liposarcoma, FNCLCC grade 2, size 6 cm.
The surgical margins are free.
Distance to margins:
Distal/lower - 2cm.
Superior/proximal 2cm
Medial - 1.5 cm
Lateral - 1 cm
Anterior - 0.9 cm
Posterior - 0.9 cm
Transitional areas are present but definite round cell componenet was identified.
Negative for necrosis.
P53 immunostain focal positive.

Nanopore sequencing report

SARC-18

Quality control metrics

Barcode statistics

```
Adapters detected in 58026 of 58628 reads
         RBK004  58026: |  ################### |  98.97 %
           none    600: |                      |   1.02 %
Barcodes detected in 58026 of 58628 adapters
      barcode03  58026: |  ################### |  98.97 %
           none    600: |                      |   1.02 %
2 reads were skipped due to the min. length filter.
Demultiplexing finished in 45.31s
```

Read statistics

```
General summary:
Average percent identity:           92.7
Fraction of bases aligned:            0.9
Mean read length:                2,638.0
Mean read quality:                  12.1
Median percent identity:            93.6
Median read length:                986.0
Median read quality:                12.2
Number of reads:                55,829.0
Read length N50:                 6,997.0
STDEV read length:               4,124.3
Total bases:               147,279,527.0
Total bases aligned:       139,137,834.0
Number, percentage and megabases of reads above quality cutoffs
>Q5:    55823 (100.0%) 147.3Mb
```

Mean genome coverage is 0.05X.

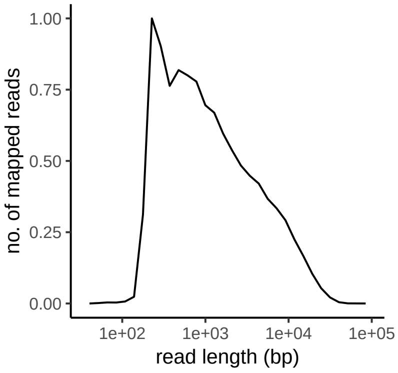

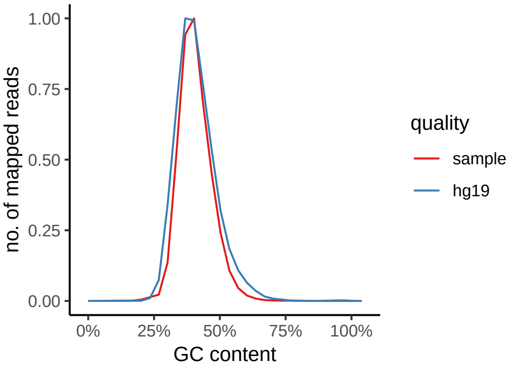

Copy number profile

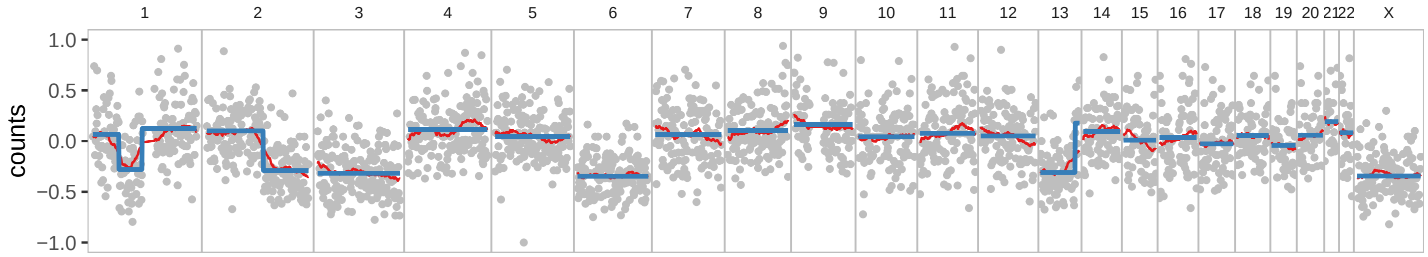

Methylation based classification

Methylation-based classification is based on **9745** CpG sites (overlap of sites covered in this sample and the training set). The out-of-bag error of this ad-hoc classifier is **2.4%**. Using this classifier, the sample has been classified as **chondrosarcoma (group A)**. This prediction has a confidence score of **0.53**. The raw and calibrated score distribution for the Top 10 entities is given below.

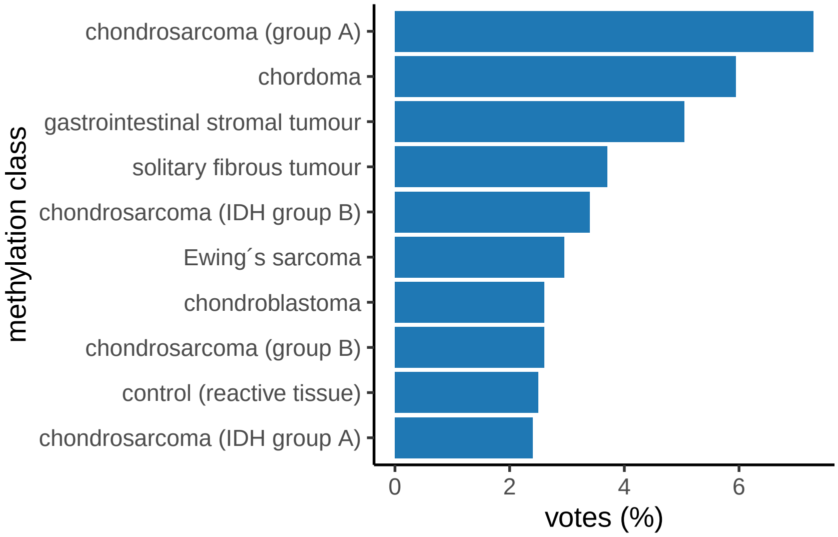

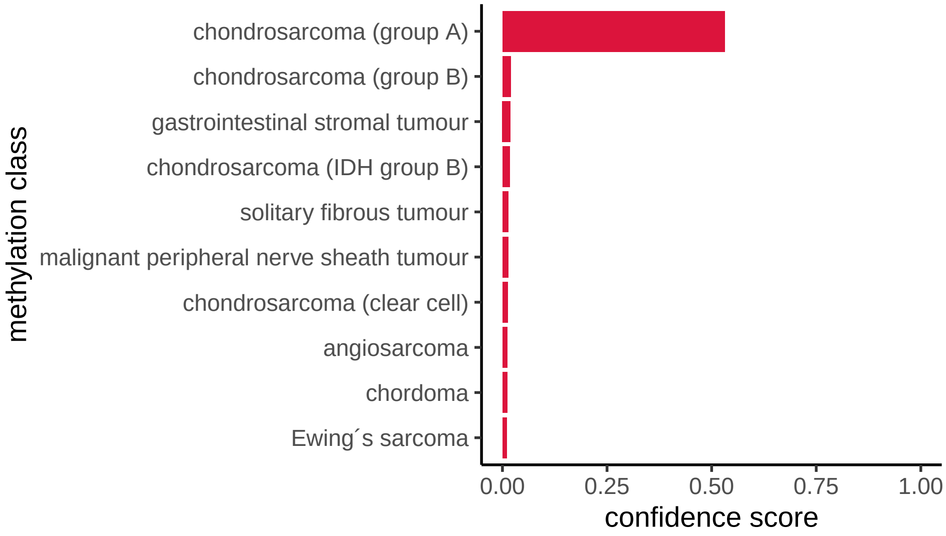

t-SNE plot

t-SNE plots are meant for visual quality control, not classification, and must be interpreted with care.

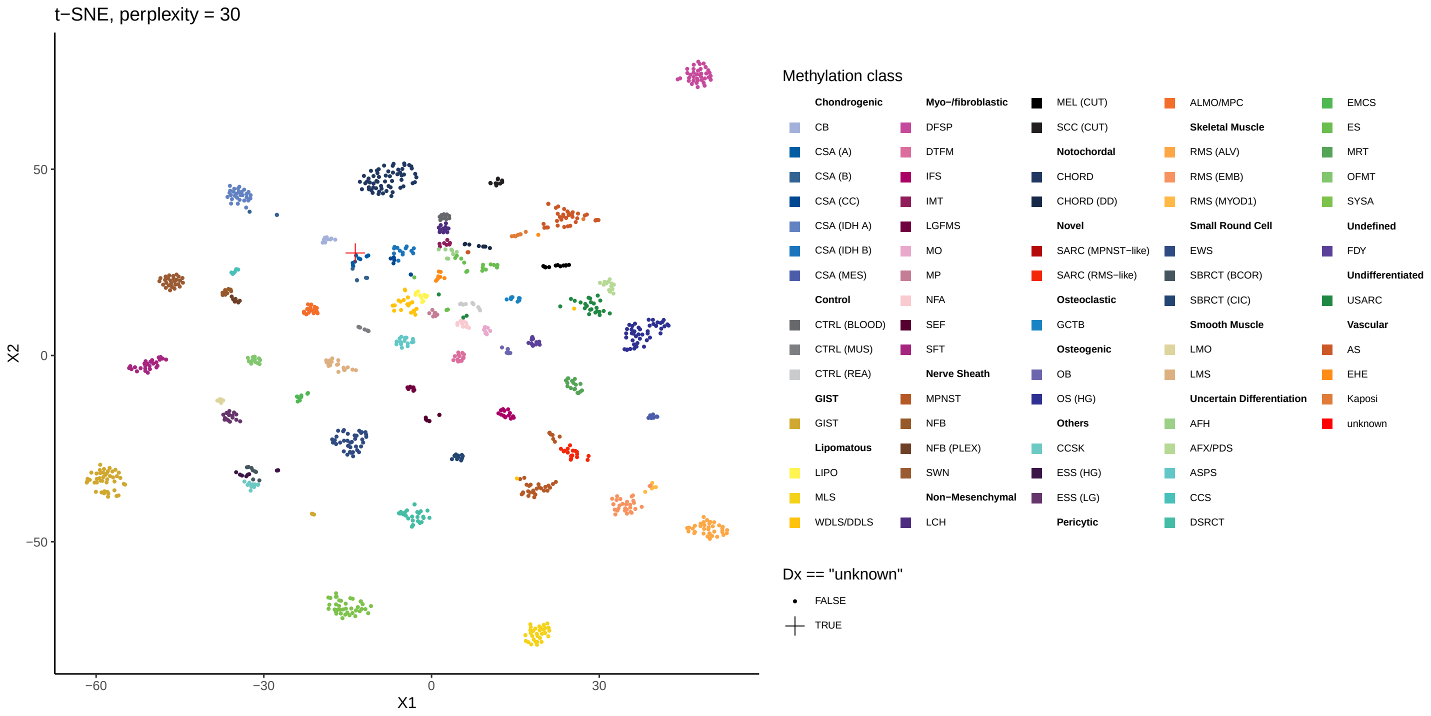

PATHOLOGY MICROSCOPIC

Paraspinal lesion, mid back, excision:

Chondrosarcoma, moderately differentiated, grade 2, completely excised

The tumor is very close to the posterior surgical margin (<1mm)

Nanopore sequencing report

SARC-19

Quality control metrics

Barcode statistics

```
Adapters detected in 23356 of 23637 reads
         RBK004  23356: |  ################### |  98.81 %
           none    279: |                      |   1.18 %
Barcodes detected in 23356 of 23637 adapters
      barcode02      1: |                      |   0.00 %
      barcode04  23355: |  ################### |  98.81 %
           none    279: |                      |   1.18 %
2 reads were skipped due to the min. length filter.
Demultiplexing finished in 27.81s
```

Read statistics

```
General summary:
Average percent identity:           92.3
Fraction of bases aligned:           1.0
Mean read length:                7,078.3
Mean read quality:                  12.4
Median percent identity:            93.7
Median read length:              3,455.0
Median read quality:                12.5
Number of reads:                22,415.0
Read length N50:                15,816.0
STDEV read length:               9,030.3
Total bases:               158,661,138.0
Total bases aligned:       153,130,277.0
Number, percentage and megabases of reads above quality cutoffs
>Q5:    22406 (100.0%) 158.7Mb
```

Mean genome coverage is 0.05X.

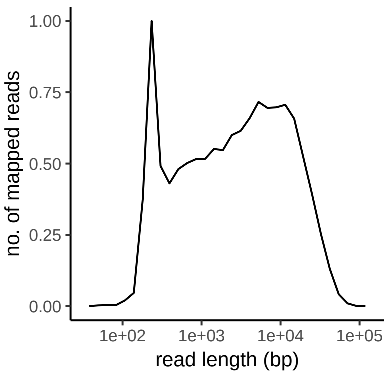

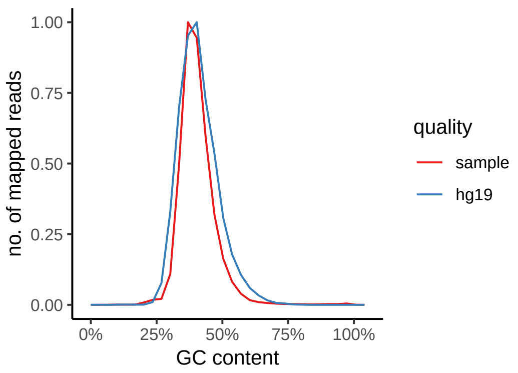

Copy number profile

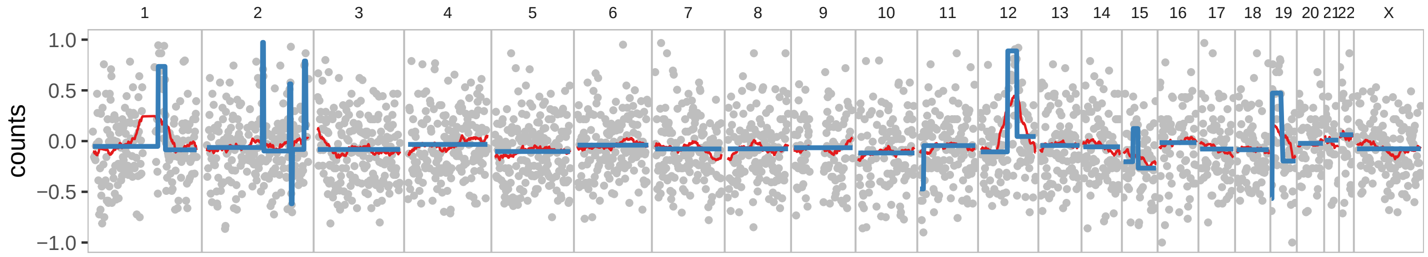

Methylation based classification

Methylation-based classification is based on **11948** CpG sites (overlap of sites covered in this sample and the training set). The out-of-bag error of this ad-hoc classifier is **1.9%**. Using this classifier, the sample has been classified as **well- / dedifferentiated liposarcoma**. This prediction has a confidence score of **0.14**. The raw and calibrated score distribution for the Top 10 entities is given below.

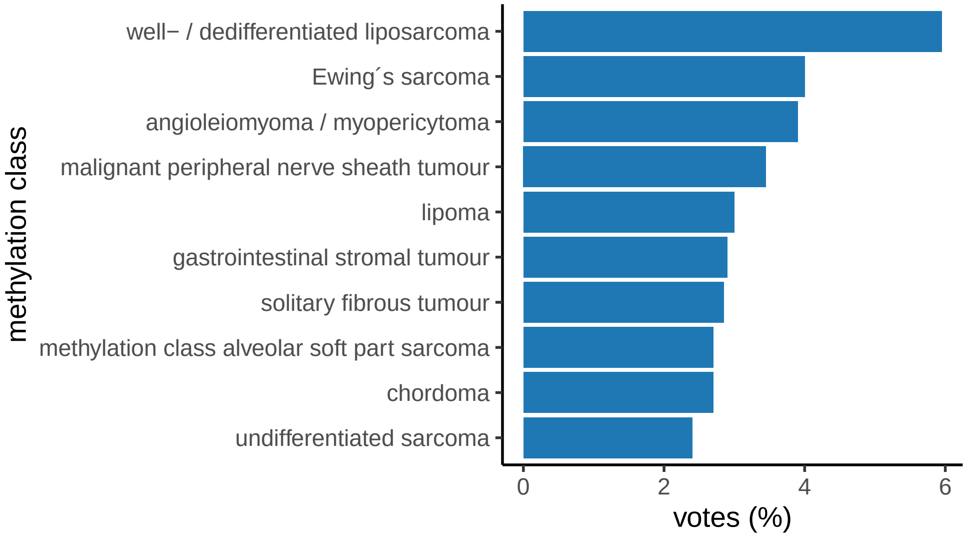

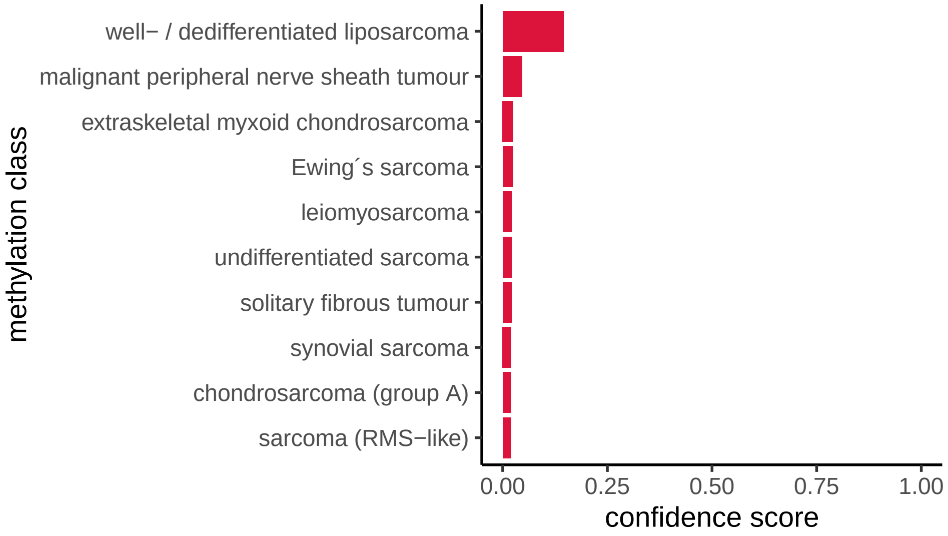

t-SNE plot

t-SNE plots are meant for visual quality control, not classification, and must be interpreted with care.

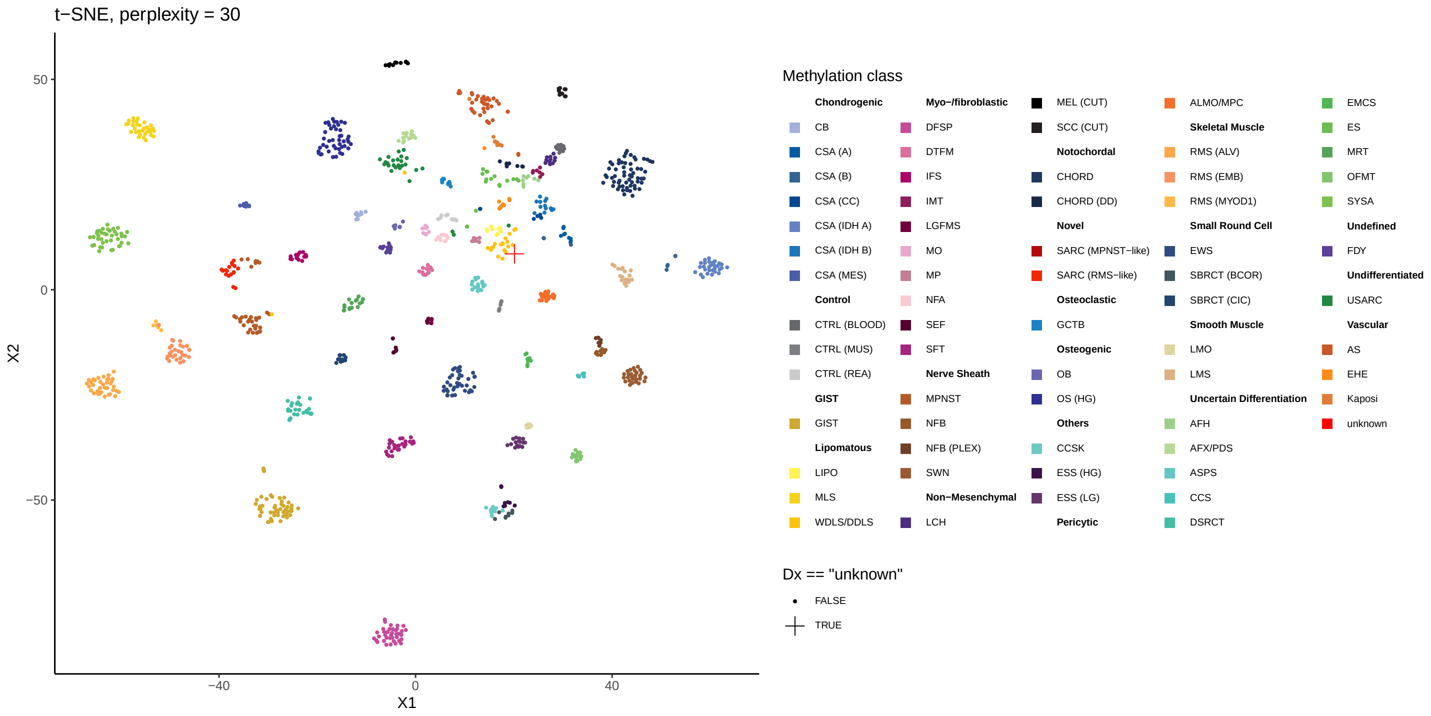

<u>PATHOLOGY MICROSCOPIC</u>

Soft tissue, Rt. thigh excision;
Well differentiated liposarcoma, with extensive mixoid areas
Free surgical margins.
Minimal distance:
anterior 0.8mm
lateral 2mm
medial 2.18mm.
Other margins are more than 3mm away.
A large nerve is seen embedded in the tumor mass.

Nanopore sequencing report

SARC-21 - SARC-21 NAME

Quality control metrics

Barcode statistics

```
Adapters detected in 50030 of 50611 reads
         RBK004  50030: |  ################### |  98.85 %
           none    581: |                      |   1.15 %
Barcodes detected in 50030 of 50611 adapters
      barcode06  50030: |  ################### |  98.85 %
           none    581: |                      |   1.15 %
Demultiplexing finished in 26.43s
```

Read statistics

```
General summary:
Average percent identity:           93.0
Fraction of bases aligned:            0.9
Mean read length:                2,622.5
Mean read quality:                  12.2
Median percent identity:            94.0
Median read length:                947.0
Median read quality:                12.2
Number of reads:                48,714.0
Read length N50:                 6,954.0
STDEV read length:               4,078.8
Total bases:                127,752,872.0
Total bases aligned:        121,057,179.0
Number, percentage and megabases of reads above quality cutoffs
>Q5:    48713 (100.0%) 127.8Mb
```

Mean genome coverage is 0.04X.

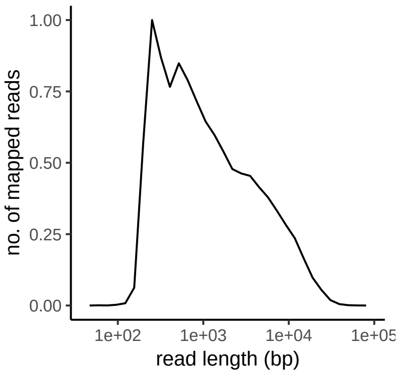

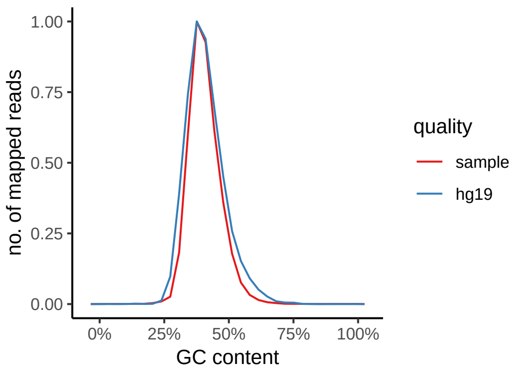

Copy number profile

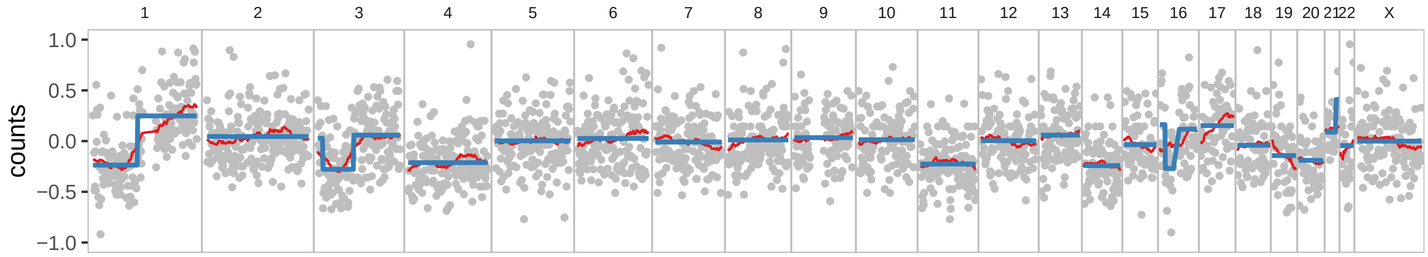

Methylation based classification

Methylation-based classification is based on **9934** CpG sites (overlap of sites covered in this sample and the training set). The out-of-bag error of this ad-hoc classifier is **2.5%**. Using this classifier, the sample has been classified as **synovial sarcoma**. This prediction has a confidence score of **0.23**. The raw and calibrated score distribution for the Top 10 entities is given below.

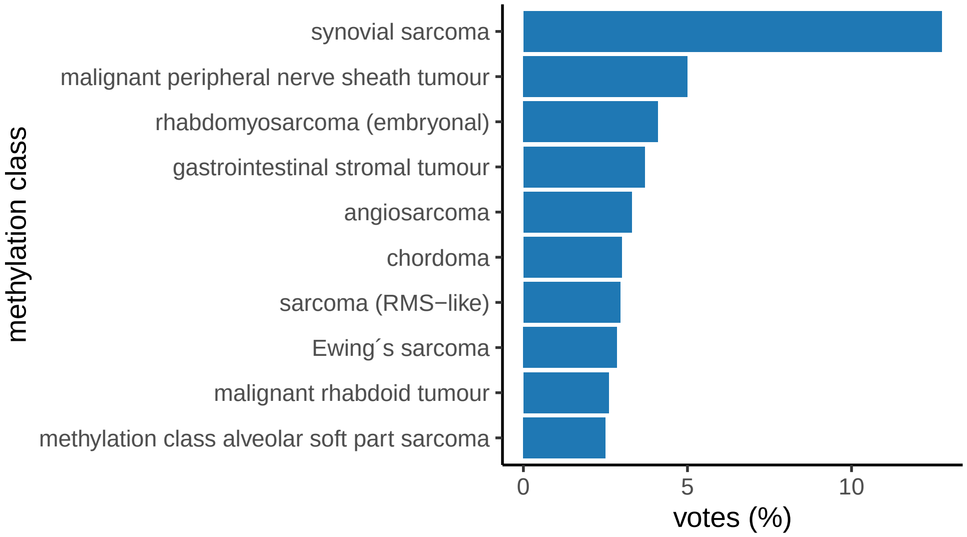

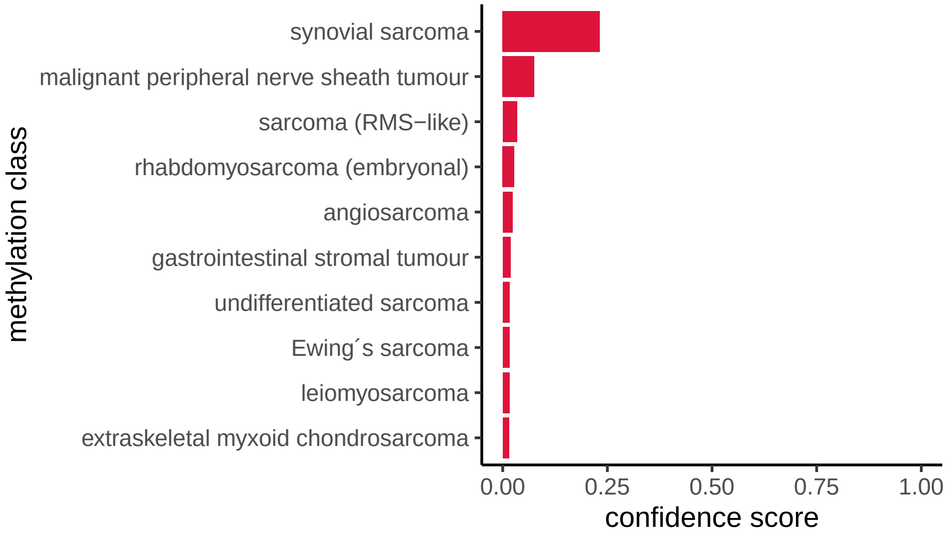

t-SNE plot

t-SNE plots are meant for visual quality control, not classification, and must be interpreted with care.

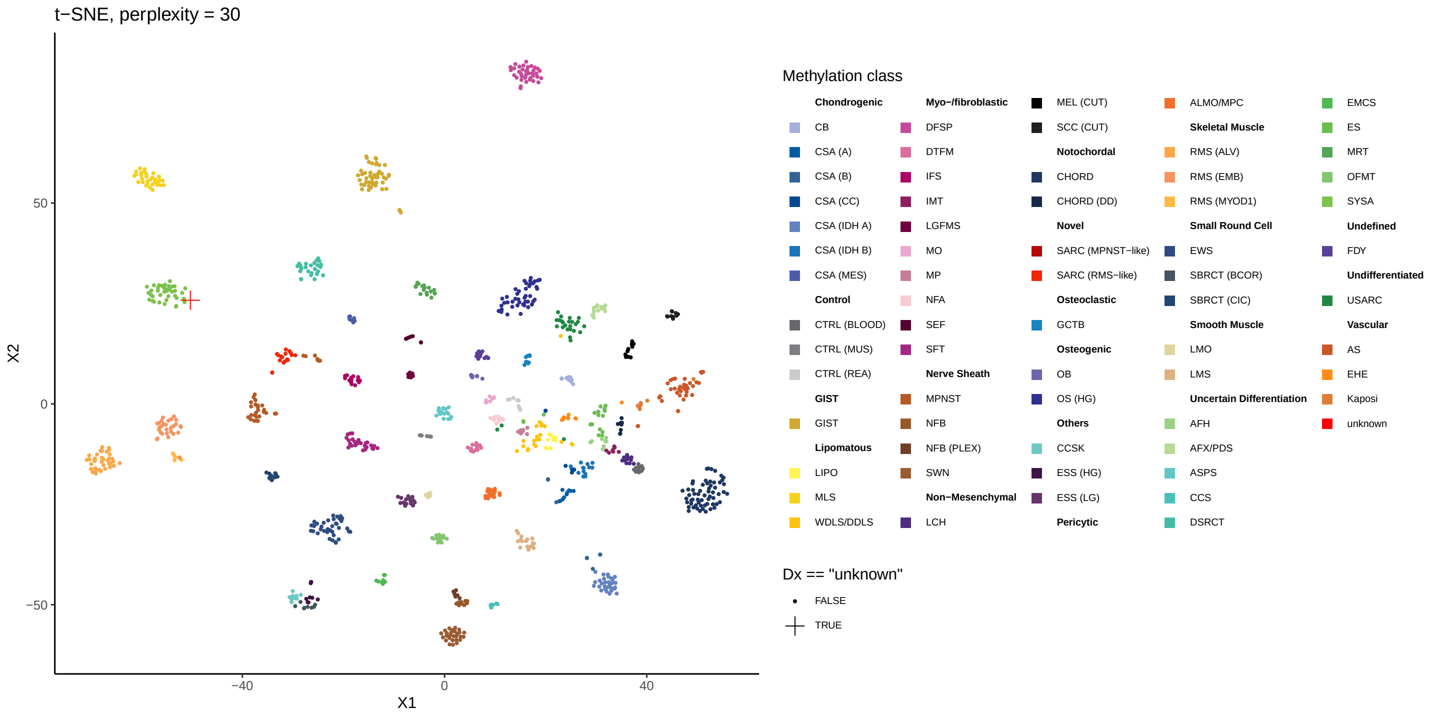

PATHOLOGY MICROSCOPIC

Total laryngectomy:
Metastatic synovial sarcoma involving the whole right glottic wall.
The tumor bulges to the lumen and causes mucosal ulceration.
The radial and distal surgical margins are free.

Nanopore sequencing report

SARC-22

Quality control metrics

Barcode statistics

```
Adapters detected in 49540 of 49987 reads
         RBK004  49540: |  ################### |  99.11 %
           none    443: |                      |   0.89 %
Barcodes detected in 49540 of 49987 adapters
      barcode07  49540: |  ################### |  99.11 %
           none    443: |                      |   0.89 %
4 reads were skipped due to the min. length filter.
Demultiplexing finished in 27.26s
```

Read statistics

```
General summary:
Average percent identity:           92.2
Fraction of bases aligned:            0.9
Mean read length:                2,748.7
Mean read quality:                  11.9
Median percent identity:            93.1
Median read length:              1,367.0
Median read quality:                12.0
Number of reads:                47,450.0
Read length N50:                 5,751.0
STDEV read length:               3,494.8
Total bases:               130,424,712.0
Total bases aligned:       123,706,537.0
Number, percentage and megabases of reads above quality cutoffs
>Q5:    47442 (100.0%) 130.4Mb
```

Mean genome coverage is 0.04X.

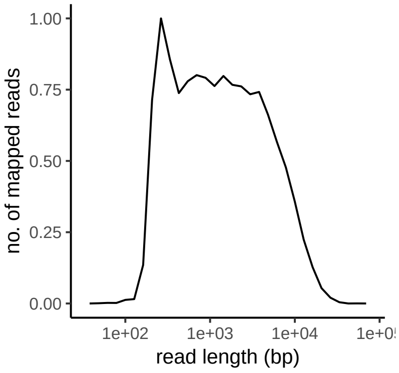

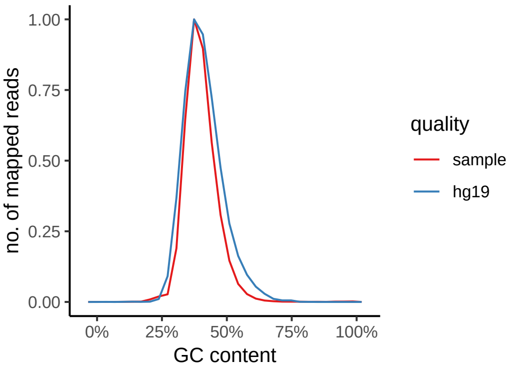

Copy number profile

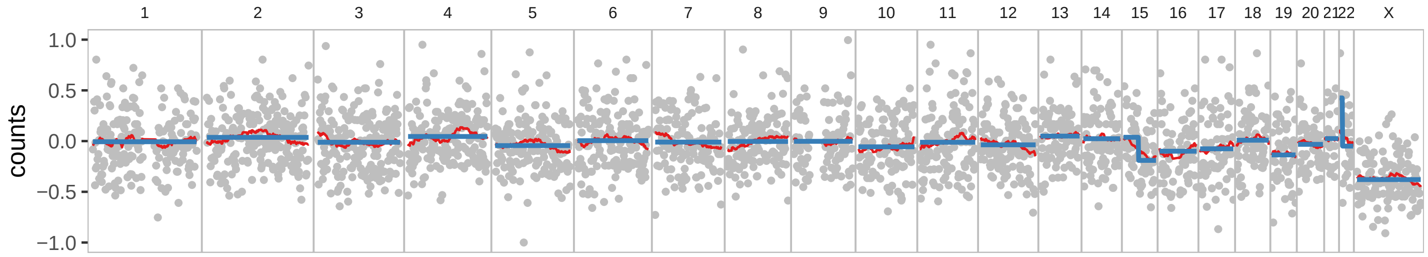

Methylation based classification

Methylation-based classification is based on **8132** CpG sites (overlap of sites covered in this sample and the training set). The out-of-bag error of this ad-hoc classifier is **2.5%**. Using this classifier, the sample has been classified as **angiomatoid fibrous histiocytoma**. This prediction has a confidence score of **0.04**. The raw and calibrated score distribution for the Top 10 entities is given below.

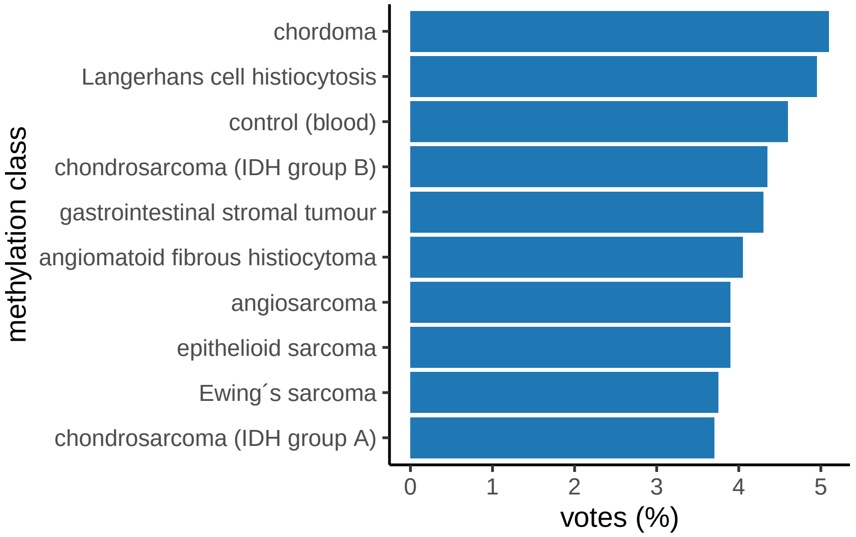

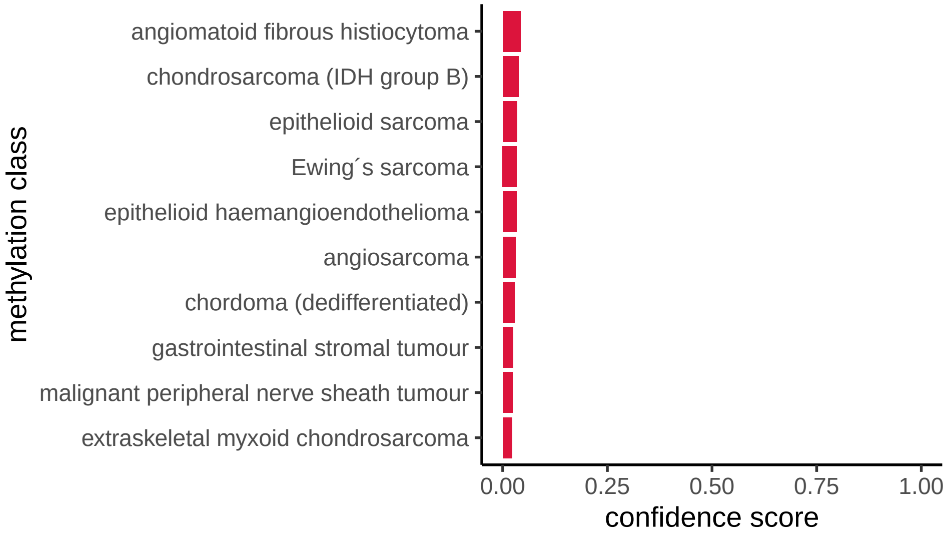

t-SNE plot

t-SNE plots are meant for visual quality control, not classification, and must be interpreted with care.

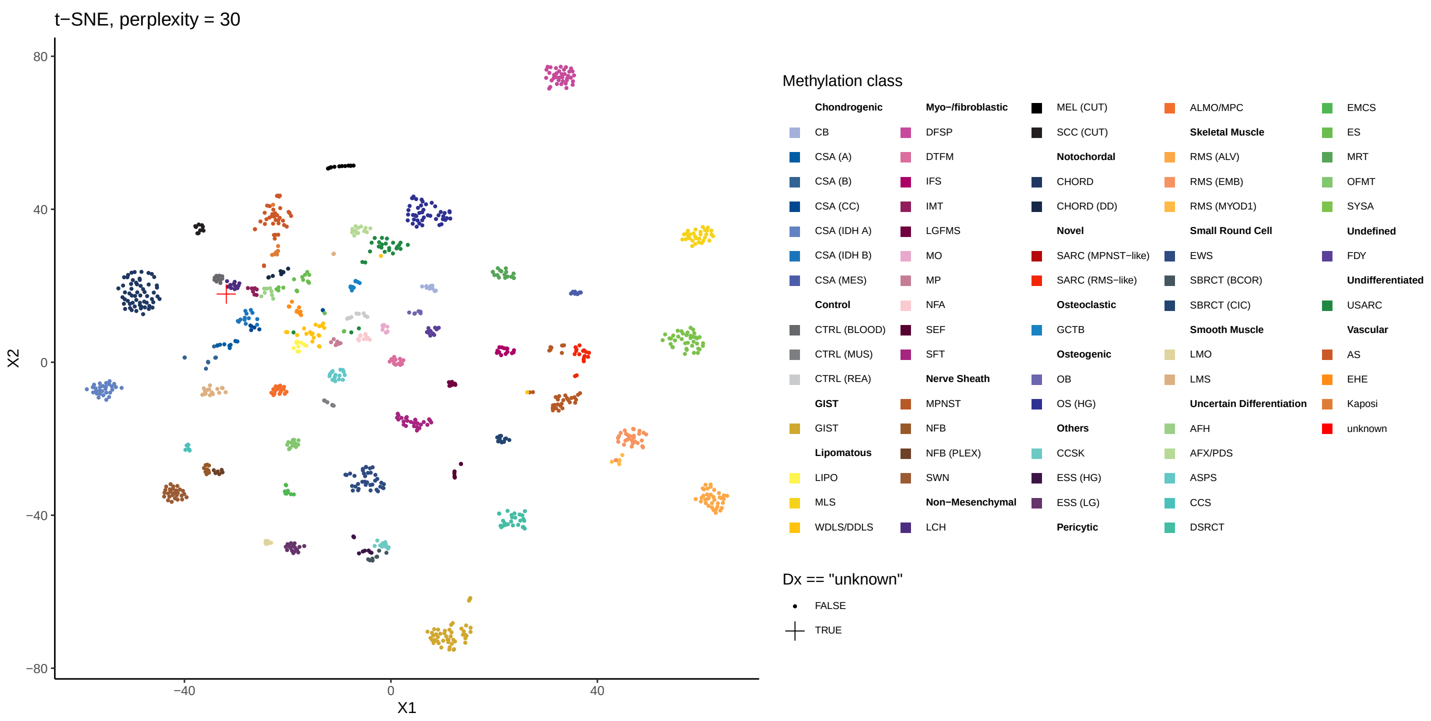

PATHOLOGY MICROSCOPIC

Subcutaneous lesion, thigh, side not specified, excision:

Myxofibrosarcoma, high grade.
Surgical margins - negative for tumor (~4 mm from the closest margins [posterior] and at least 2.5 cm away from all other margins).

Nanopore sequencing report

SARC-23

Quality control metrics

Barcode statistics

```
Adapters detected in 46155 of 46642 reads
         RBK004  46155: |  ################### |  98.96 %
           none    484: |                      |   1.04 %
Barcodes detected in 46155 of 46642 adapters
      barcode03  46155: |  ################### |  98.96 %
           none    484: |                      |   1.04 %
3 reads were skipped due to the min. length filter.
Demultiplexing finished in 28.87s
```

Read statistics

```
General summary:
Average percent identity:           92.1
Fraction of bases aligned:            1.0
Mean read length:                5,768.8
Mean read quality:                  12.0
Median percent identity:            93.3
Median read length:              2,598.0
Median read quality:                12.1
Number of reads:                44,474.0
Read length N50:                13,514.0
STDEV read length:               7,895.2
Total bases:               256,560,622.0
Total bases aligned:       247,580,012.0
Number, percentage and megabases of reads above quality cutoffs
>Q5:    44454 (100.0%) 256.6Mb
```

Mean genome coverage is 0.08X.

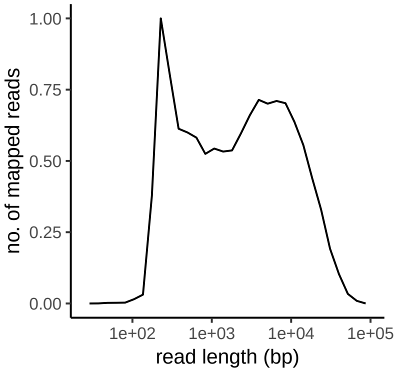

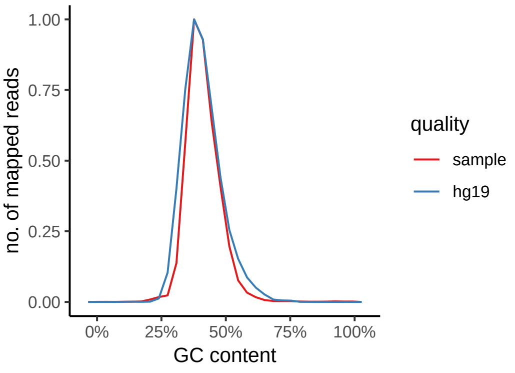

Copy number profile

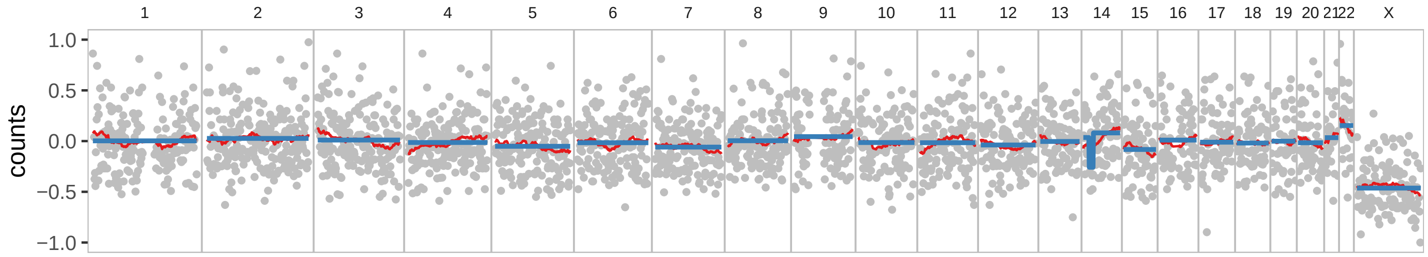

Methylation based classification

Methylation-based classification is based on **20895** CpG sites (overlap of sites covered in this sample and the training set). The out-of-bag error of this ad-hoc classifier is **1.9%**. Using this classifier, the sample has been classified as **malignant peripheral nerve sheath tumour**. This prediction has a confidence score of **0.05**. The raw and calibrated score distribution for the Top 10 entities is given below.

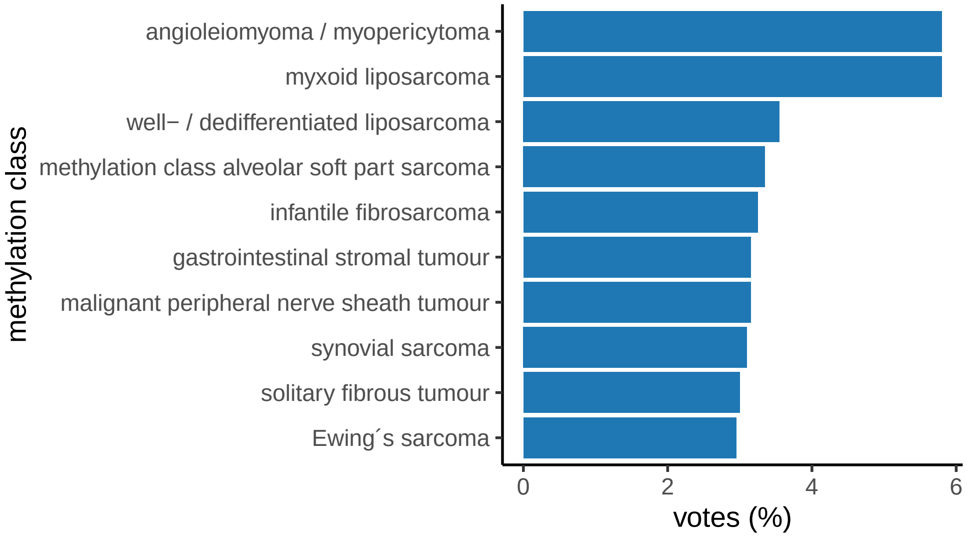

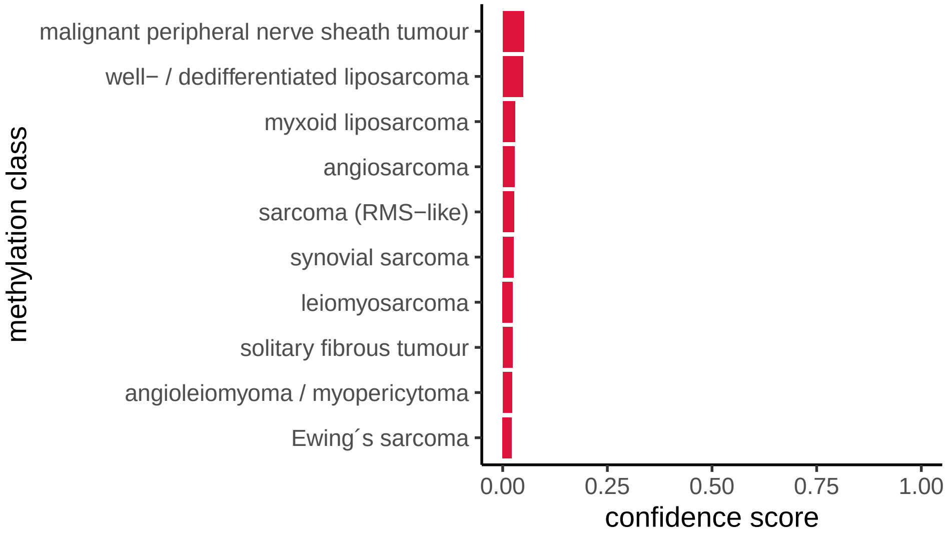

t-SNE plot

t-SNE plots are meant for visual quality control, not classification, and must be interpreted with care.

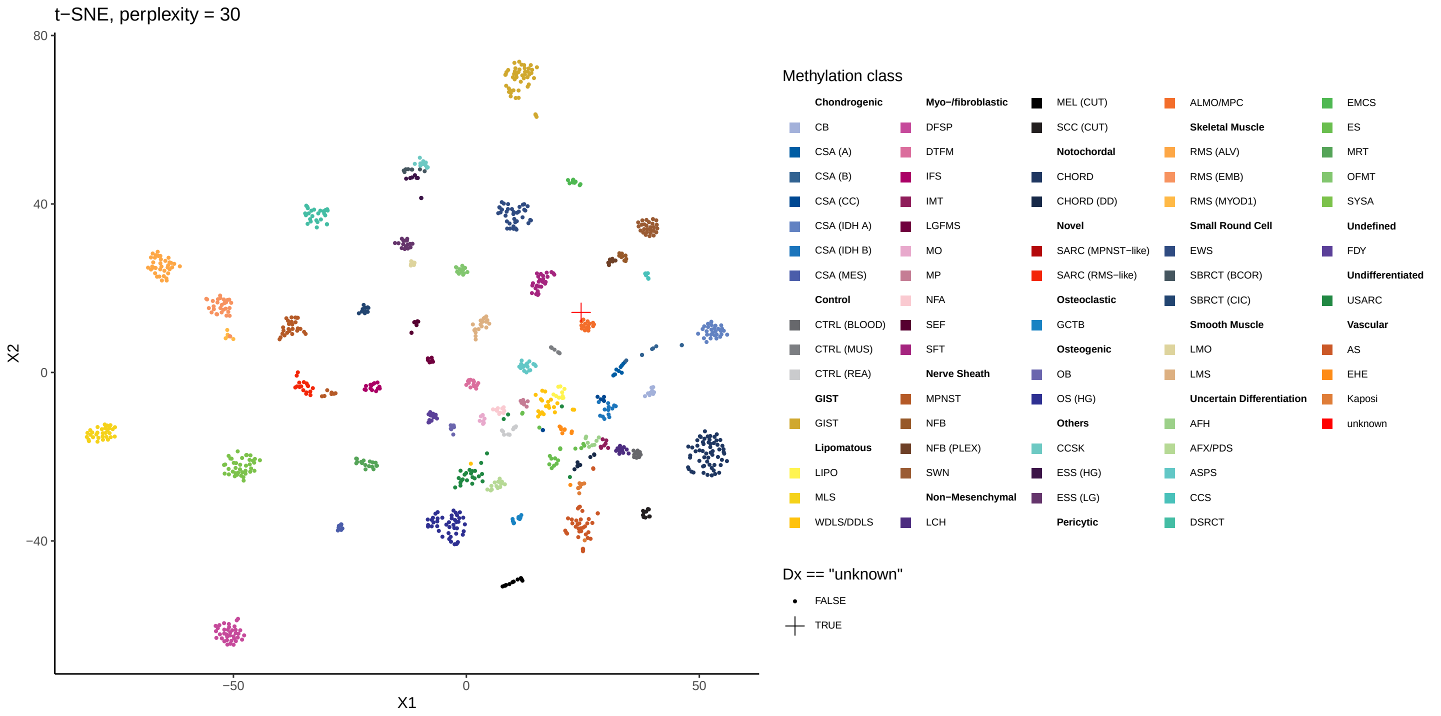

PATHOLOGY MICROSCOPIC

Residual post treatment myxoid liposarcoma
